# An irregular hourglass pattern describes the tempo of phenotypic development in placental mammal evolution

**DOI:** 10.1098/rsbl.2020.0087

**Published:** 2020-05-13

**Authors:** Gerardo A. Cordero, Marcelo R. Sánchez-Villagra, Ingmar Werneburg

**Affiliations:** 1 Fachbereich Geowissenschaften, Eberhard Karls Universität Tübingen, Germany; 2 Senckenberg Centre for Human Evolution and Palaeoenvironment at Eberhard Karls Universität Tübingen, Germany; 3 Palaeontological Institute and Museum, University of Zurich, Switzerland

**Keywords:** hourglass, phylotypic period, mammalian development, comparative embryology

## Abstract

Organismal development is defined by progressive transformations that ultimately give rise to distinct tissues and organs. Thus, temporal shifts in ontogeny often reflect key phenotypic differences in phylogeny. Classical theory predicts that interspecific morphological divergence originates towards the end of embryonic or fetal life stages, i.e. the early conservation model. By contrast, the hourglass model predicts interspecific variation early and late in prenatal ontogeny, though with a phylogenetically similar mid-developmental period. This phylotypic period, however, remains challenging to define within large clades such as mammals. Thus, molecular and morphological tests on a mammalian hourglass have not been entirely congruent. Here, we report an hourglass-like pattern for mammalian developmental evolution. By comparing published data on the timing of 74 homologous characters across 51 placental species, we demonstrated that variation in the timing of development decreased late in embryogenesis––when organ formation is highly active. Evolutionary rates of characters related to this timeframe were lowest, coinciding with a phylotypic period that persisted well beyond the pharyngula ‘stage’. The trajectory culminated with elevated variation in a handful of fetal and perinatal characters, yielding an irregular hourglass pattern. Our study invites further quantification of ontogeny across diverse amniotes and thus challenges current ideas on the universality of developmental patterns.

## 1. Introduction

Organismal development is a continuum characterized by progressive cellular-scale changes and growth, i.e. ontogeny [1]. In embryos, such developmental processes are temporally dynamic and necessary for the formation of tissues, organs and traits that define the adult condition. Thus, temporal alterations in ontogeny are expected to explain phenotypic differences across lineages that have descended from a common ancestor, i.e. phylogeny [1,2]. In the nineteenth century, Karl Ernst von Baer proposed that embryos of phylogenetically distant species share a common set of morphological traits at the onset of ontogeny. Subsequently, he observed that such evolutionarily conserved embryonic traits underwent a series of transformations that gave rise to species-specific morphotypes, i.e. the early conservation model [3]. Alternatively, the hourglass model predicts early and late phases of ontogenetic divergence linked by a highly morphologically similar mid-developmental period, known as the phylotypic period [1,4].

The hourglass model was initially motivated by observations of evolutionarily conserved gene expression patterns (e.g. *Hox* gene colinearity) during mid-embryogenesis [5], as well as comparative embryological studies that hinted at the existence of a phylotypic period in both vertebrate and invertebrate animals [1,4]. Recently, the exploration of genome-wide transcriptional activity (i.e. gene expression) across diverse animal and plant taxa revealed temporal trends that were somewhat consistent with the hourglass model [6–9]. This is perhaps best exemplified by landmark studies that traced gene expression during organogenesis (embryogenesis) in placental mammals [6,8]. Crucially, molecular hourglass hypotheses are predicated upon the existence of high interspecific phenotypic similarity during the phylotypic period [7], though this is rarely cross-validated via comparisons of embryo morphology across phylogenetically divergent lineages (but see [9]).

Placental mammals have long been of interest to comparative embryologists [2,3,10]. Indeed, observations made beginning during the golden age of comparative embryology later enabled the first quantitative test of the hourglass model by Bininda-Emonds *et al*. [11]. Their thorough examination of developmental sequences across 14 representative mammals suggested that the phylotypic period is rather characterized by high interspecific variation [11]. Here, we build upon this foundational work by demonstrating that variation decreases towards the end embryogenesis. Subsequently, elevated variation in fetal and perinatal characters resulted in an irregular hourglass-like pattern for the overall ontogenetic trajectory of placental mammals.

## 2. Material and methods

**Figure 1.**
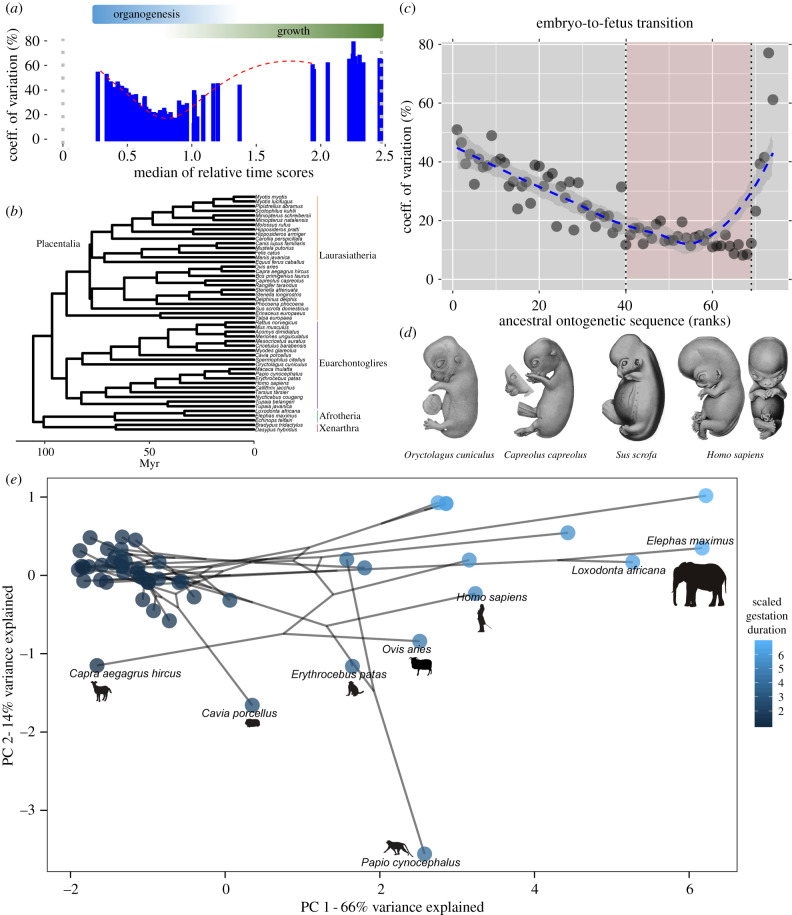
The relative timing of 112 characters spanning organogenesis (embryogenesis) and growth (fetal period) in 62 placental mammals; data extracted by Werneburg *et al*. [12] (*a*). After filtering, the coefficient of variation for the relative timing of 74 characters was regressed (Loess fit) against the hypothetical ancestral sequence of 51 species (*b*,*c*). In (*d*), the embryo-to-fetus transition of reduced variation (red box in *c*) is depicted in representative placentals (modified from Keibel's *Normentafeln*, e.g. [10,14–16]; see electronic supplementary material, figure S13). A pPCA on relative timing indicates a directional trend along the first principal component axis (PC 1) that corresponds to gestation duration in large-bodied species (blue gradient in (*e*); see electronic supplementary material, figure S11 for mapped transitions).

Analyses were based on a dataset of mammalian developmental characters compiled mainly from the primary literature by Werneburg *et al*. [12]. Homologous characters were ranked by their order of appearance [12,13] (figure 1*a*; electronic supplementary material, figure S1–3). Data were transformed such that their chronological order was on a continuous scale from 0 (fertilization) to 1 (end of embryogenesis) (see [12,13]). This relative timing scale facilitated interspecific comparisons, while controlling for gestation duration [12,17] (figure 1*a*). After data filtering, 74 characters were selected across 51 species (electronic supplementary material, figure S1–10; supplementary file 1). Missing relative timing scores were phylogenetically imputed and ancestral states were reconstructed, via maximum likelihood, using the *Rphylopars* R package [18]. Reconstructed values were rank-ordered to generate a hypothetical ancestral ontogenetic sequence.

Coefficients of variation (i.e. the mean-standardized variation) for relative timing were regressed against the ancestral sequence using local regression (Loess), which does not require *a priori* assumptions on the mean response. Using *Phytools* [19], variation was explored with a phylogenetic principal component analysis (pPCA). Rates of evolution for relative timing were computed and the likelihood of featuring distinct rates to the likelihood of a common rate was tested [20]. Comparative phylogenetic analyses, if applicable, assumed a Brownian motion model of evolution. Further details on data processing and analyses are listed in the electronic supplementary material.

## 3. Results

**Figure 2.**
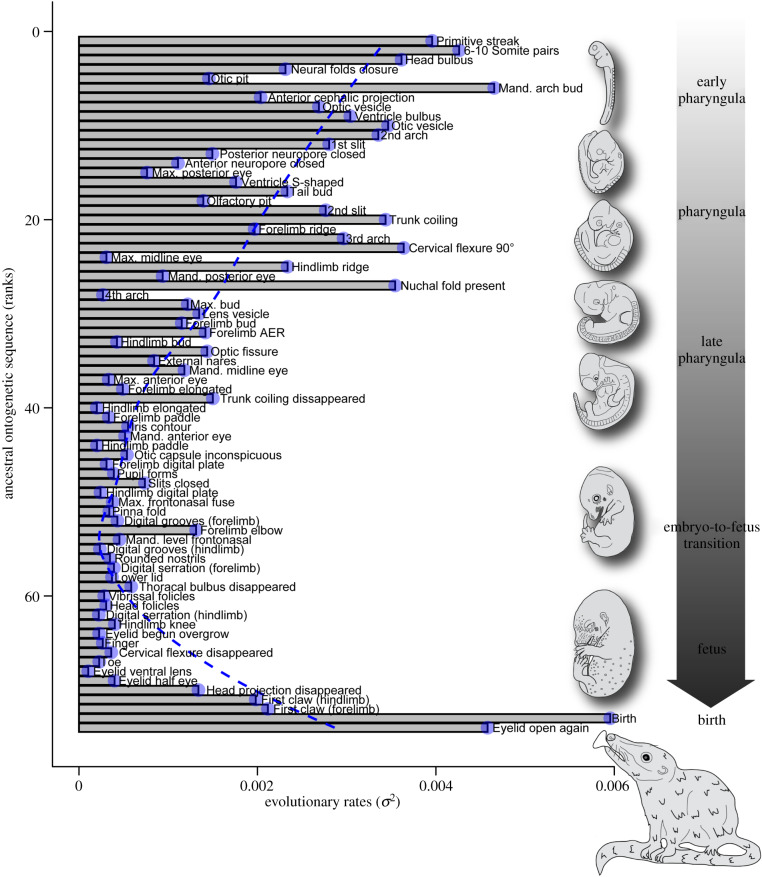
Evolutionary rates for the relative timing of characters are listed following the hypothetical ancestral sequence for placentals. A Loess regression fit was applied to the data to visualize the mean trend (blue dashed line) across ontogeny. Characters associated with the embryo-to-fetus transition (ranks 40–68) featured the lowest rates of evolution.

Variation in the relative timing of characters was initially high and reached a minimum near the end of primary organogenesis, i.e. the embryo-to-fetus transition (figure 1; electronic supplementary material, figures S2–3, S9). Subsequent variation was mainly driven by integumental (hair and claw) characters in fetuses, as well as perinatal characters. Thus, the pPCA on relative timing revealed a directional trend associated with gestation duration, with PC 1 dominated by characters ‘birth’, ‘eyelid open again’ and ‘primitive streak’. PC 2 loadings tracked characters ‘slits closed’, ‘maxillary and frontonasal fuse’, ‘first claw (hindlimb)’ and ‘first claw (forelimb)’ (figure 1*e*; electronic supplementary material, figures S11–12; tables S1–2). Rates of evolution for the relative timing of characters associated with the embryo-to-fetus transition were lowest (sequence ranks 40–68) (figure 2, electronic supplementary material, figures S13–15; table S2). The likelihood that these rates differed was supported (likelihood [L] ratio: 6273.1; L_observed_ = 2450.5, L_constrained_ = −686.1, *p* < 0.0001); see electronic supplementary material, figures S14–15 for non-overlapping 95% confidence intervals.

## 4. Discussion

Whether variation in the timing of developmental processes resembles an hourglass continues to intrigue evolutionary, developmental and molecular biologists [1,4–7,9,21]. Given the complexity of developmental processes and the vagaries of atomizing developmental transformations into features coded in a comparative series of many species, it is not surprising that our findings do not reflect a ‘perfect’ hourglass. We do, however, report an *hourglass*-like pattern in placental mammals. In fact, our study is one of few to have quantified differences in the relative timing of developmental processes across an extensive timeframe in a large clade and it is, as such, a *bona fide* test of the shape of variation in development. Our work exemplifies how fundamental it is to consider the nature of the data (e.g. genetic versus phenotypic) and its temporal scope before reaching conclusions about the universality of a developmental pattern. An overview of the comparative embryological literature, e.g. [11,22,23], leads us to predict that future studies will uncover similar hourglass-like patterns in amniotes, though possibly with key differences. It is also important to highlight that the hourglass model, though useful and productive as a metaphor [24], is an oversimplification of a complex biological reality [25].

The relative timing of characters was most variable early in embryogenesis of placental mammals, possibly owing to interspecific differences in the timing and activation of early developmental processes. Placental mammals exhibit temporal differences in zygote implantation, as well as in the establishment of germ layers (blastula and gastrula formation) and nervous system (neurulation) [26]. Moreover, the differentiation of mesodermal derivatives, such as somites, may vary within and among species [4]. Much of this variation likely incurs negligible phenotypic consequences, i.e. developmental burden [21], as many primordial embryo components are transient and somewhat independent from one another [22]. In agreement, we showed that evolutionary change in the timing of development is likely to occur early in ontogeny. Thereafter, the relative timing of characters associated with the embryo-to-fetus transition exhibited the lowest rates of evolution. During this time, variation in the relative timing of characters may reach a minimum because high organogenic activity reinforces tissue or organ developmental interdependence [22]. Thus, evolution may favour stability during this critical window of inductive interactions (e.g. gene co-expression) that precedes the growth-driven fetal period. We propose that the phylotypic period encompasses this timeframe and is shifted well beyond the pharyngula ‘stage’ in placentals. This corroborates that the phylotypic period should occur later in ontogeny when examining less inclusive clades [27].

We primarily compared homologous characters that describe universally shared changes during organogenesis [12]. Consequently, our temporal coverage during which many species-specific morphotypes (apomorphies) emerge in fetuses was necessarily sparse. Although a few characters drove variation in fetuses, it is unequivocal that interspecific divergence is augmented during this growth-dominated period [3,7,21]. Also, fetal and perinatal variation is intrinsically linked to gestation duration in mammals with diverse life histories [28], as accounted for in our analyses. Altogether, our results differed to those of the ‘inverted’ hourglass model of Bininda-Emonds *et al*. [11], probably owing to differences in character definition, methodology and taxonomic breadth. Bininda-Emonds *et al*. [11] compared the presence/absence of characters in 14 mammals across multiple temporal windows, whereas we quantified temporal variation by treating chronological character sequences of 51 species as continuous variables. Still, our study agrees with their assertion that the patterns of variation in mid-embryogenesis are critical to phenotypic evolution in mammals [11]. For example, craniofacial morphology in humans and rodents is most similar during the phylotypic period [23]. Furthermore, consistent with our phenotypic comparisons, genomic regulatory dynamics in late pharyngula embryos exhibit conservative and highly interrelated trends that later become phylogenetically divergent during the fetal period of placental mammals [6,9]. However, whether variation in the relative timing of early development mirrors interspecific differences in gene expression remains to be explored.

Whether the phylotypic period influences evolvability is a relevant hourglass hypothesis worthy of further investigation [21,25]. This challenging endeavour requires integration of classical approaches with genomic techniques that characterize inductive interactions in development [6,8,9]. The hourglass model is an effective theoretical tool to address the tempo of organismal development within a macroevolutionary framework [1,7,21], though it is a ‘fuzzy’ concept (as proposed by Richardson) that must be empirically tested and interpreted with caution [11,25]. By overcoming challenges to comparing a standardized metric for variation across ontogeny and phylogeny (see [24]), we succeeded in quantitatively testing the hourglass. Our comparative approach provides impetus to further examine diverse species while advancing theory on the variational properties of developing organisms.

## Supplementary Material

Supplementary Methods and Results

## Supplementary Material

Data

## Data accessibility

Data are available as electronic supplementary material.
