# Supplementary material for: An irregular hourglass pattern describes the tempo of phenotypic development in placental mammal evolution
Source: Biol Lett. 2020 May 13;16(5):20200087. doi: 10.1098/rsbl.2020.0087 (PMC7280037; doi:10.1098/rsbl.2020.0087)
Supplement: Supplementary Methods and Results [file rsbl20200087supp1.pdf]

Electronic Supplementary Material

An irregular hourglass pattern describes the tempo of phenotypic development in placental mammal evolution

G.A. Cordero, M.R. Sánchez-Villagra, and I. Werneburg

1. Supplementary methods

(a) Character data

Progressive developmental changes in embryos of placental mammals were evaluated using the Standard Event System (SES) to Study Vertebrate Embryos [6]. An updated list of characters that can be potentially scored guided by the SES approach is available online (see "SES-characters" section in [8]). Data on the timing of developmental events were referenced from previously published studies that employed SES guidelines to describe mammalian embryos [1, 9] (Fig. S1-3). Following Germain and Laurin [10], species-specific sequences of developmental events (i.e. SES characters) were ordered on a continuous scale, such that 0 corresponded to fertilization and 1 to the end of embryogenesis (see Fig. S2 and Supplementary File 1). We chose the character "mandible at occlusion point" as a homologous temporal marker that could be reliably used to define the end of embryogenesis (i.e. primary organogenesis) in all species examined (Fig. S2; Supplementary File 1) [1]. Thus, values that scored > 1 were primarily associated with fetal stages, birth, and perinatal changes (Fig. S2) [1]. Transforming developmental sequences to a continuous scale permits analyses of data points that would otherwise have to be excluded, particularly when estimates for absolute embryo age and size are unavailable or ambiguous [1, 9, 11]. Furthermore, these relative timing scores can be treated as continuous variables in multivariate and comparative phylogenetic analyses [10].

Embryological descriptions available in the literature differed among studies (see appendices in Werneburg et al. [1] and Supplementary File 1). Consequently, our original data matrix exhibited a 41% missing data rate (Figs. S4-5). To optimize the number of species and characters that could be retained in our analyses, we excluded species with more than 70 missing characters, as well as characters that could not be scored in more than 40 species. This reduced the missing data rate to 21% and resulted in a final data set of 74 characters for 51 species (Figs. S6-7; Supplementary File 1). Assuming that missing data points were randomly distributed across our data matrix, a 21% missing data rate is reasonable for multivariate missing data imputation [12]. We initially performed multivariate missing data imputation using the iterative principal component analysis (PCA) algorithm of the *missMDA* R package [13]. We then compared these imputed values to a phylogenetically imputed data set (see below). Local (Loess) and linear regression fitting was performed via the *geom_smooth* function of *ggplot2* R package [14].

(b) Comparative phylogenetic analyses

All comparative phylogenetic analyses were based on a pruned topology (time-calibrated) for placental mammals, following Werneburg et al. [1] (Fig. S1). Comparative phylogenetic analyses, if applicable, assumed a Brownian motion (BM) model of evolution as a general overall measure of how traits change across a phylogeny [15]. Alternative Ornstein-Uhlenbeck and early burst models, fitted to multivariate data matrices using the *phylocurve* R package [16], were equally supported as the BM model (all log-likelihoods = 181284.4). However, we chose the simpler BM model with fewer parameters, similarly to previous comparisons for models of developmental character evolution in mammals, e.g. [1]. Using the *phylopars* function of the *Rphylopars* package [17], we repeated imputation analyses while taking phylogenetic interdependence (assuming a BM model) into account

[18]. The *phylopars* function also simultaneously estimated maximum likelihood ancestral state states for each character [17].

The rank-ordered ancestral character sequence was highly concordant with a character sequence ranked by means for relative timing in extant species (using phylogenetically and non-phylogenetically imputed data sets) (Fig. S8). Data sets imputed with *missMDA* and with *Rphylopars* were highly congruent (Fig. S8). Subsequent analyses on interspecific character variation were performed using the phylogenetically imputed data set and the rank-ordered ancestral character (developmental) sequence, though character variation was also plotted against relative timing for comparison (Fig. S9). Reconstructed ancestral states ultimately depend on the availability of trait data, as well as the taxa of choice [19]. To explore whether the chosen taxa and characters introduced biases in ancestral state reconstructions, a taxonomically and phenotypically restricted data set (N = 44 species; N = 66 characters), though with a lower missing data rate of 15%, was analyzed and compared to the analyses based on 51 species and 74 characters (21% missing data rate). Reconstructed ancestral means for the relative timing of characters and their sequence ranks were highly similar between restricted and full data sets, indicating that no major biases were introduced by performing ancestral state reconstructions on the phenotypically broader and more taxonomically inclusive data set of 51 species (Fig. S10).

To test for phylogenetic signal in our data set, we computed the multivariate version of Blomberg's K statistic (K_{mult}; [20]) using the *geomorph* R package [21]. As our data set exhibited phylogenetic signal ($K_{\mathrm{mult}} = 0.72$; $P < 0.0001$, permutations = 10,000), we explored and compared interspecific differences in the relative timing of characters by performing a phylogenetic PCA in *Phytools* [22]. Graphical inspection of the phylogenetic PCA was performed via *ggplot2* using the custom *ggphylomorpho* function (https://github.com/wabarr/ggphylomorpho). Principal components (PCs) 1 and 2 explained 80% of variation in the data set (Figs. S11-12; Table S1). PCs 1 and 2 were reconstructed for internal nodes and mapped onto the phylogeny of placental mammals using the *contMap* function of *Phytools* [23]. With the exception of Fig. S13, graphs were created using *ggplot2*, *Phytools* or base R functions.

Evolutionary rates (BM rate parameter: σ^2) for each character were estimated, using *Phytools*, by computing phylogenetic independent contrasts on log-transformed data (Table S2). To ascertain the extent to which character evolutionary rates differed among each other, we compared the likelihood of a model in which characters featured distinct evolutionary rates to the likelihood of a model in which all characters shared a common rate [15]. This test assumed a BM model of evolution and was performed using the likelihood-based methodology (including R code) developed by D.C. Adams [see 15]. Illustrated specimens exemplifying the mid-developmental period during which interspecific variation decreases and rates of evolution were lowest, see Table S2, are depicted in Fig. S13. Rates of evolution for all characters and their 95% confidence intervals are depicted in Figs. S14-15.

2. Supplementary Figures and Tables

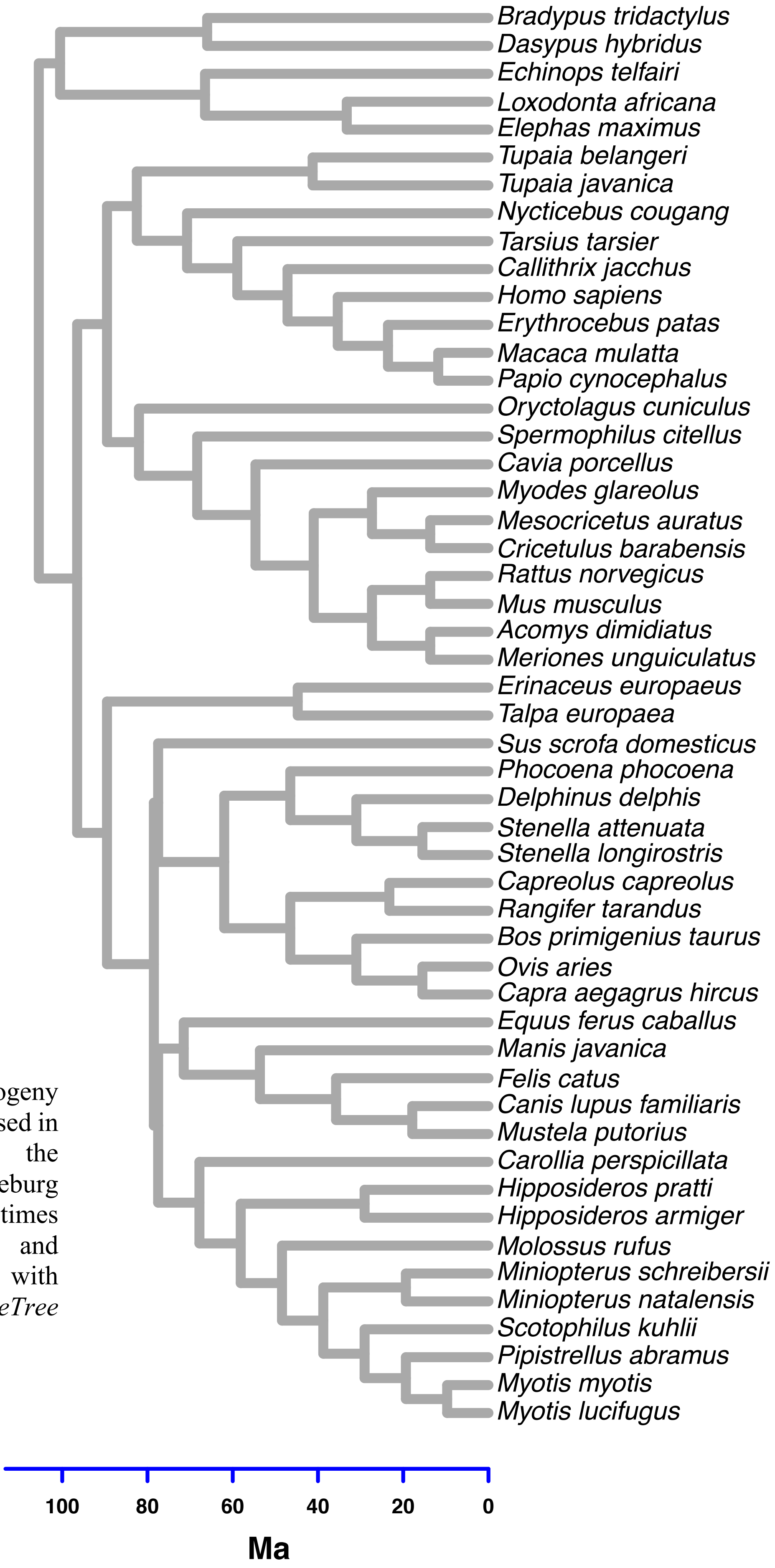

Figure S1. Pruned phylogeny of placental mammals used in analyses, based on the topology used in Werneburg et al.[1]. Divergence times were cross-referenced, and adjusted if needed, with updated data on the *TimeTree* web server [7].

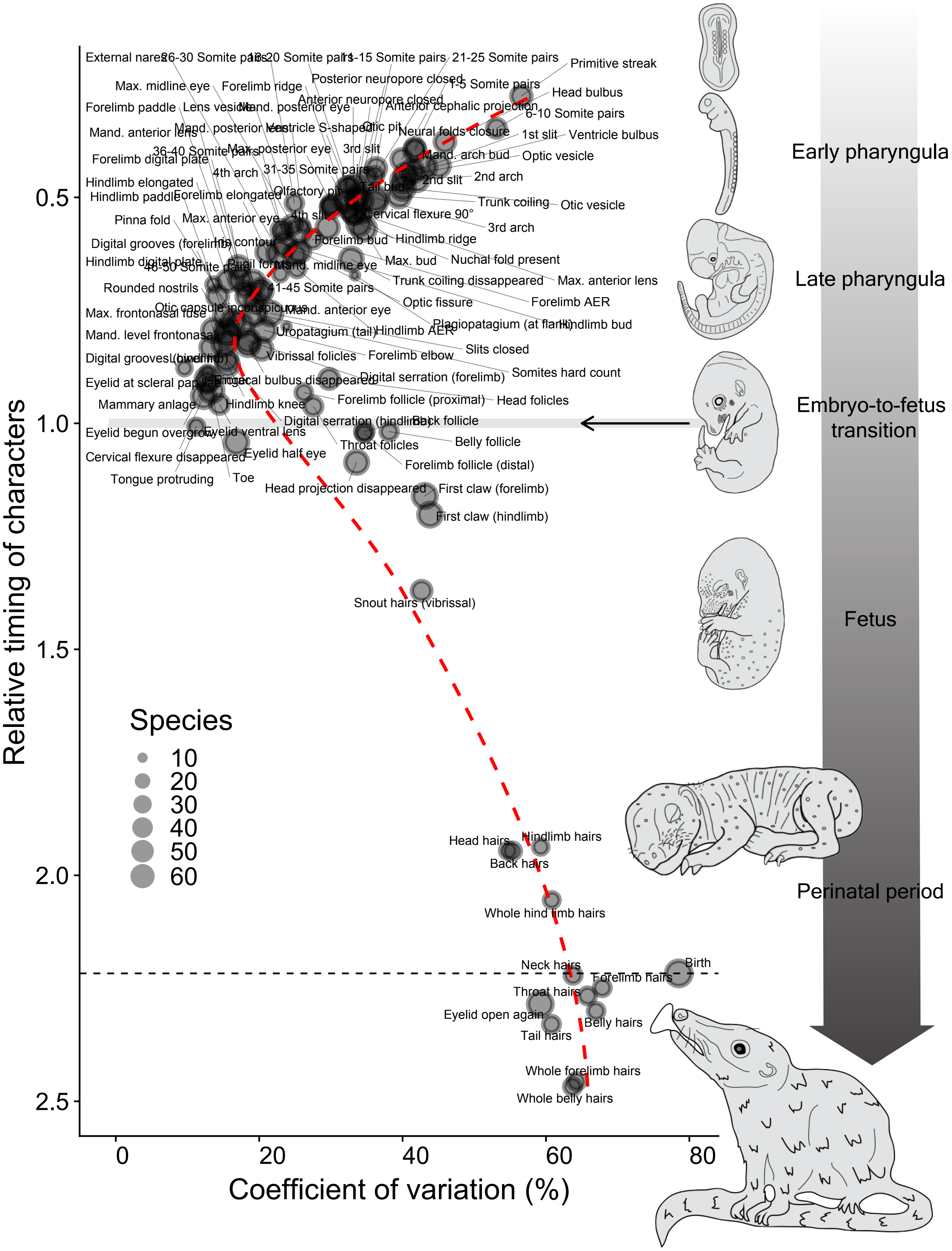

Figure S2. To enable comparisons of developmental variation in the initial data set (without imputation), standard deviations (SD) were divided by means (x̄) to yield mean-standardized coefficients of variation (CV) for each character. The CV ([SD/x̄ * 100] based on 62 species) for 112 characters is plotted against relative timing of characters (0 = fertilization; 1 = end of organogenesis or embryogenesis [see arrow] as defined in Werneburg et al. [1]). Circles are proportional to the number of species sampled per character and the mean trend line (red) is based on locally estimated scatterplot smoothing. Embryo images are modified from [24].

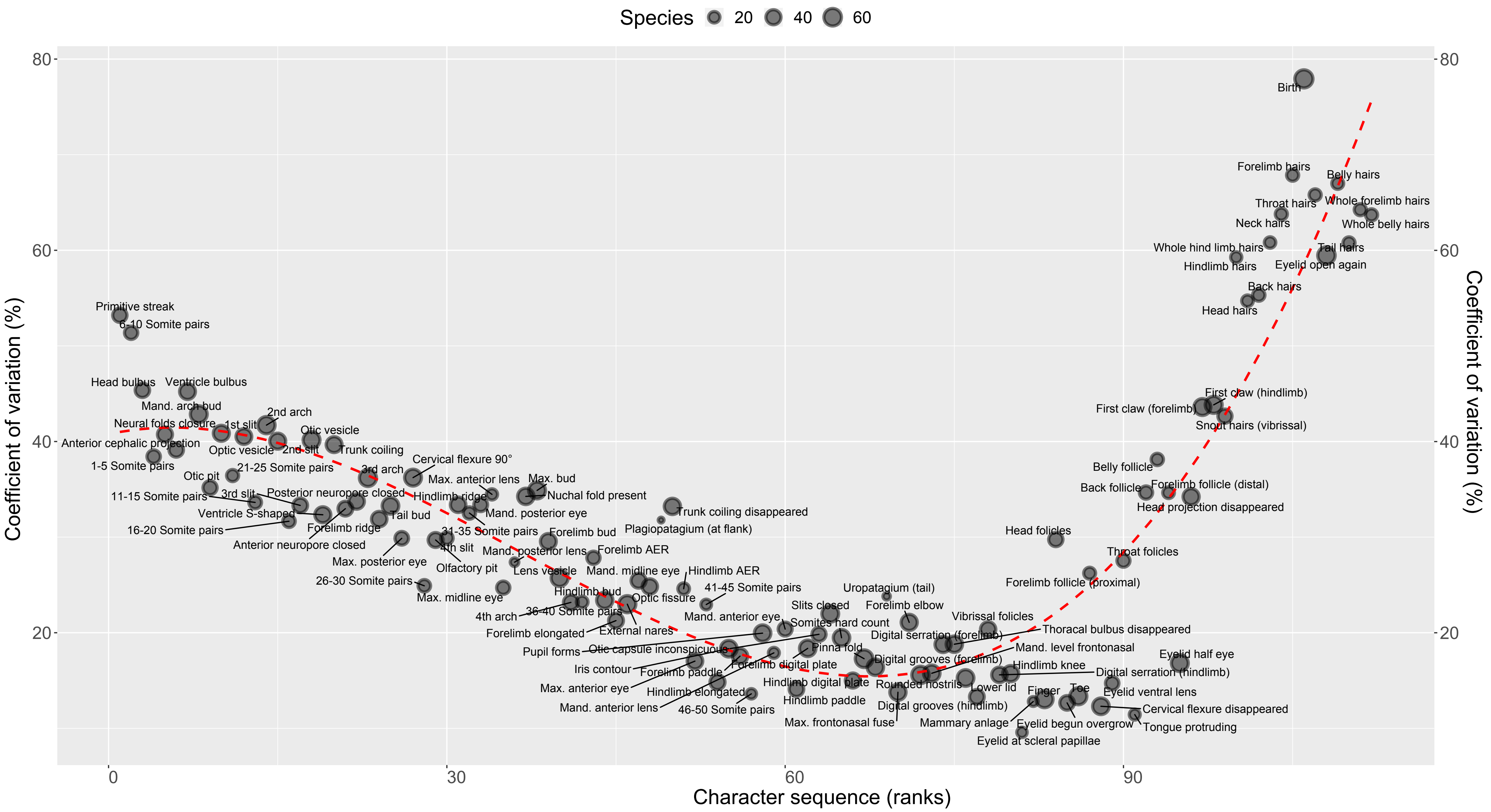

Figure S3. The coefficient of variation of 112 characters in 62 placental mammal species (data set without imputation extracted by Werneburg et al. [1]) is plotted against the rank-ordered character sequence (locally estimated scatterplot smoothing [Loess regression] was applied to the data; red dashed line). Characters were ranked according to the mean relative timing for each character. Circles are proportional to specie samples.

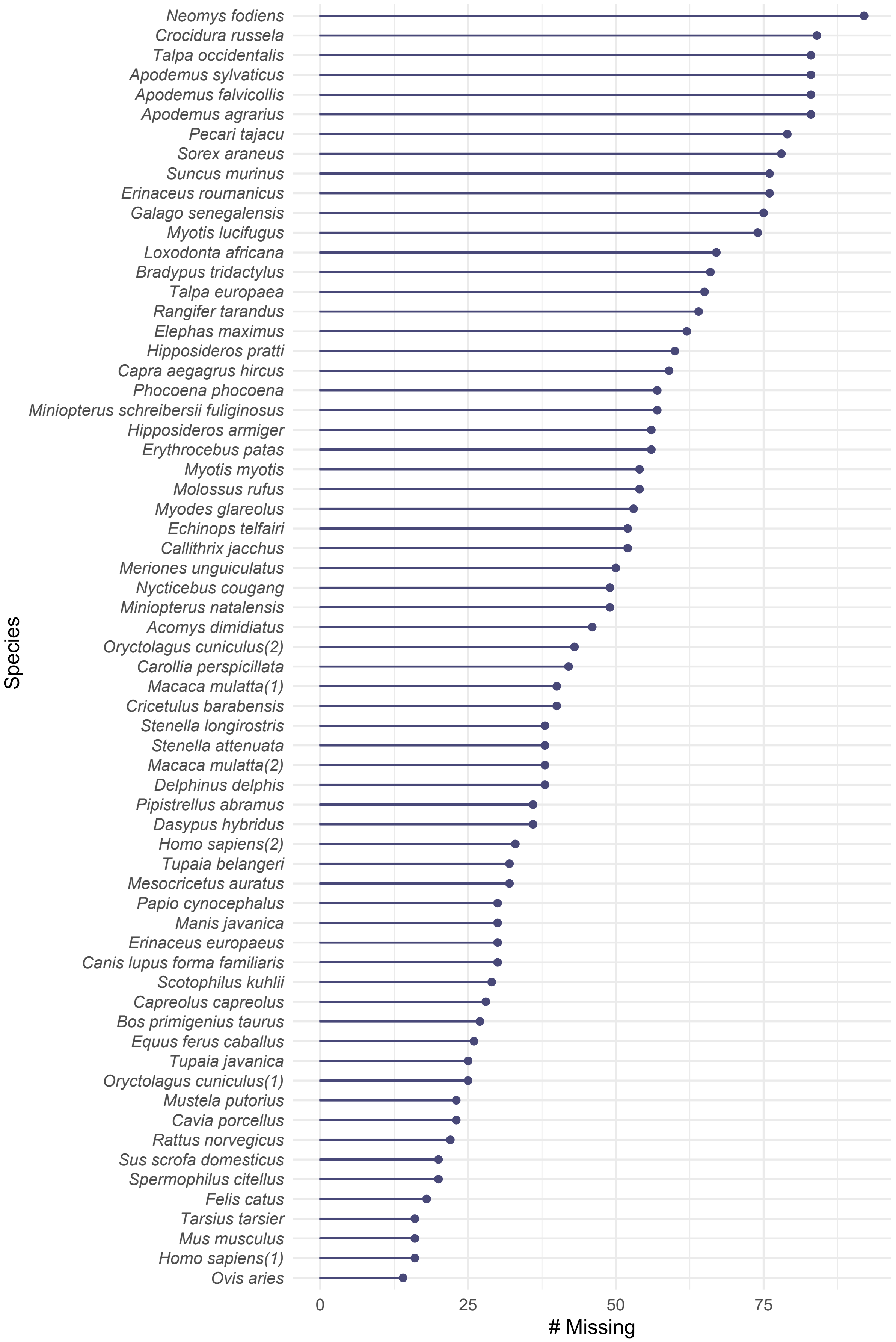

Figure S4. Missing character values per species, based on an initial survey of 112 characters in 62 placental mammal species examined in Werneburg et al. [1]. Note that some taxa were represented twice if character descriptions were extracted from two sources. We subsequently excluded species with more than 70 missing characters (see Figs. S6-7).

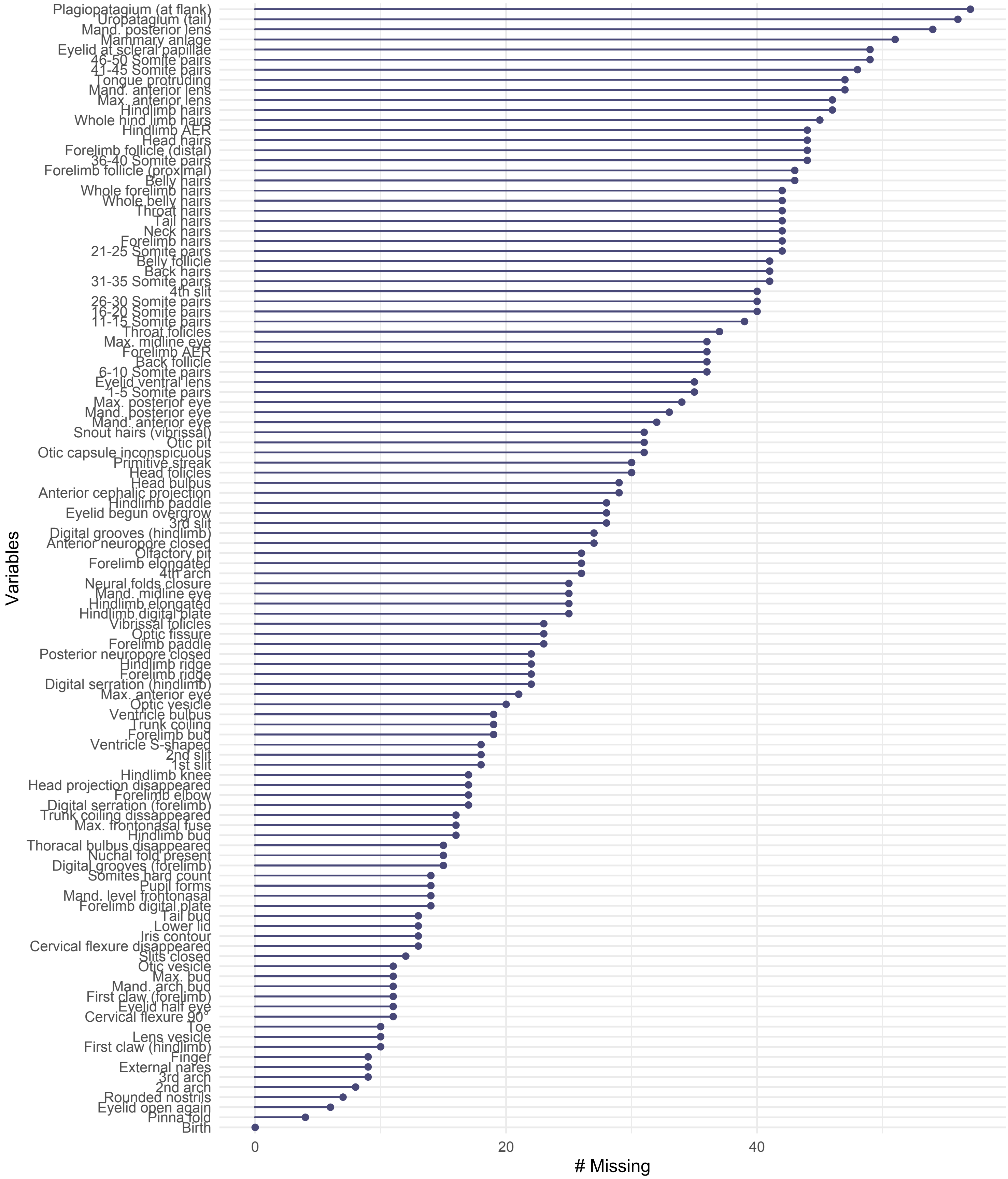

Figure S5. Missing character values, based on an initial survey of 112 characters in 62 placental mammal species. The overall missing data rate for this data set was 41%. Characters that could not be scored in more than 40 species were excluded (see Figs. S6-7).

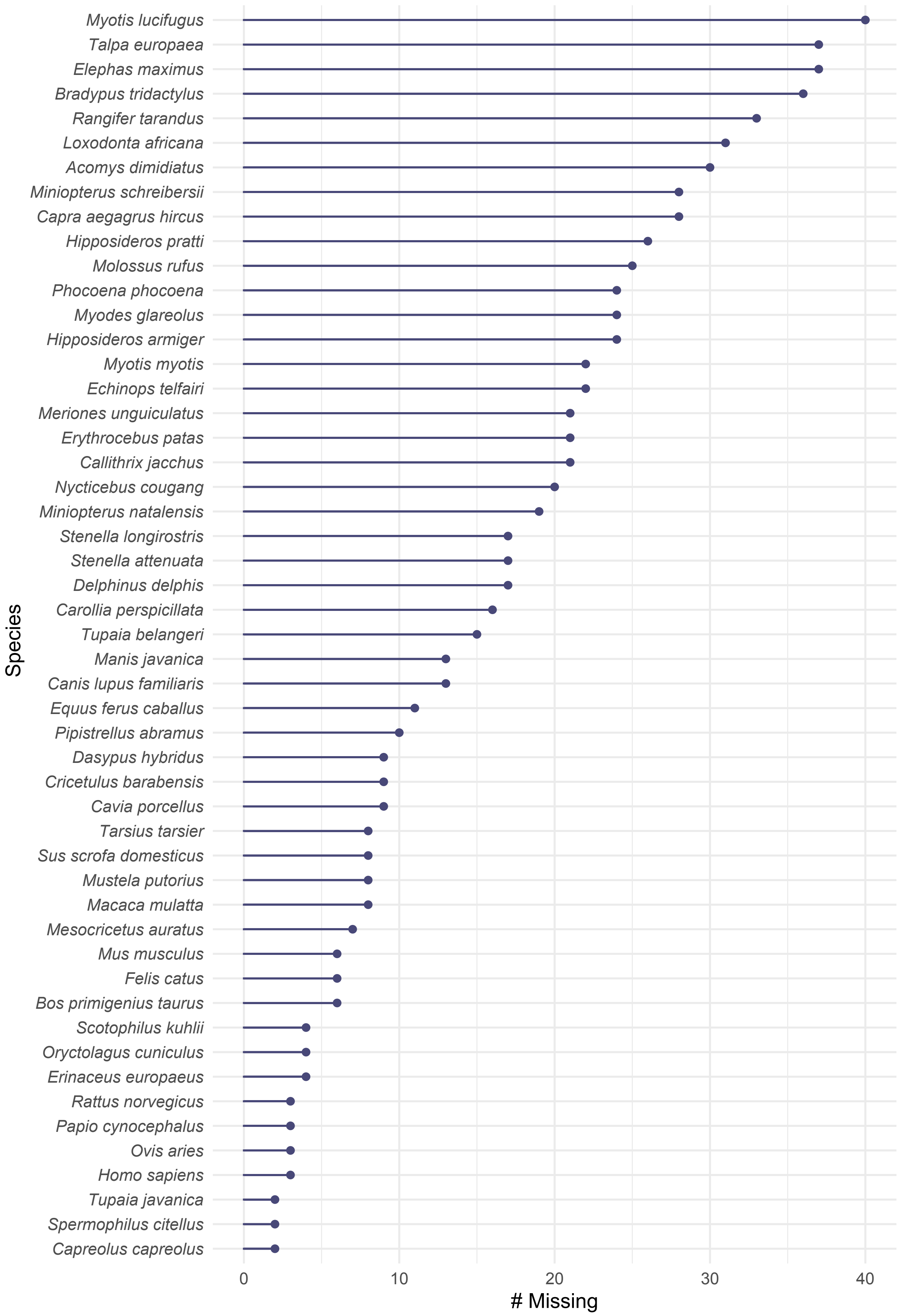

Figure S6. Missing character values per species, based on a reduced data set of 74 characters across 51 placental mammal species. If two sets of character descriptions were available per species (i.e. *Homo sapiens*, *Oryctolagus cuniculus*, *Macaca mulatta*), the one with the greatest number of missing data points was excluded.

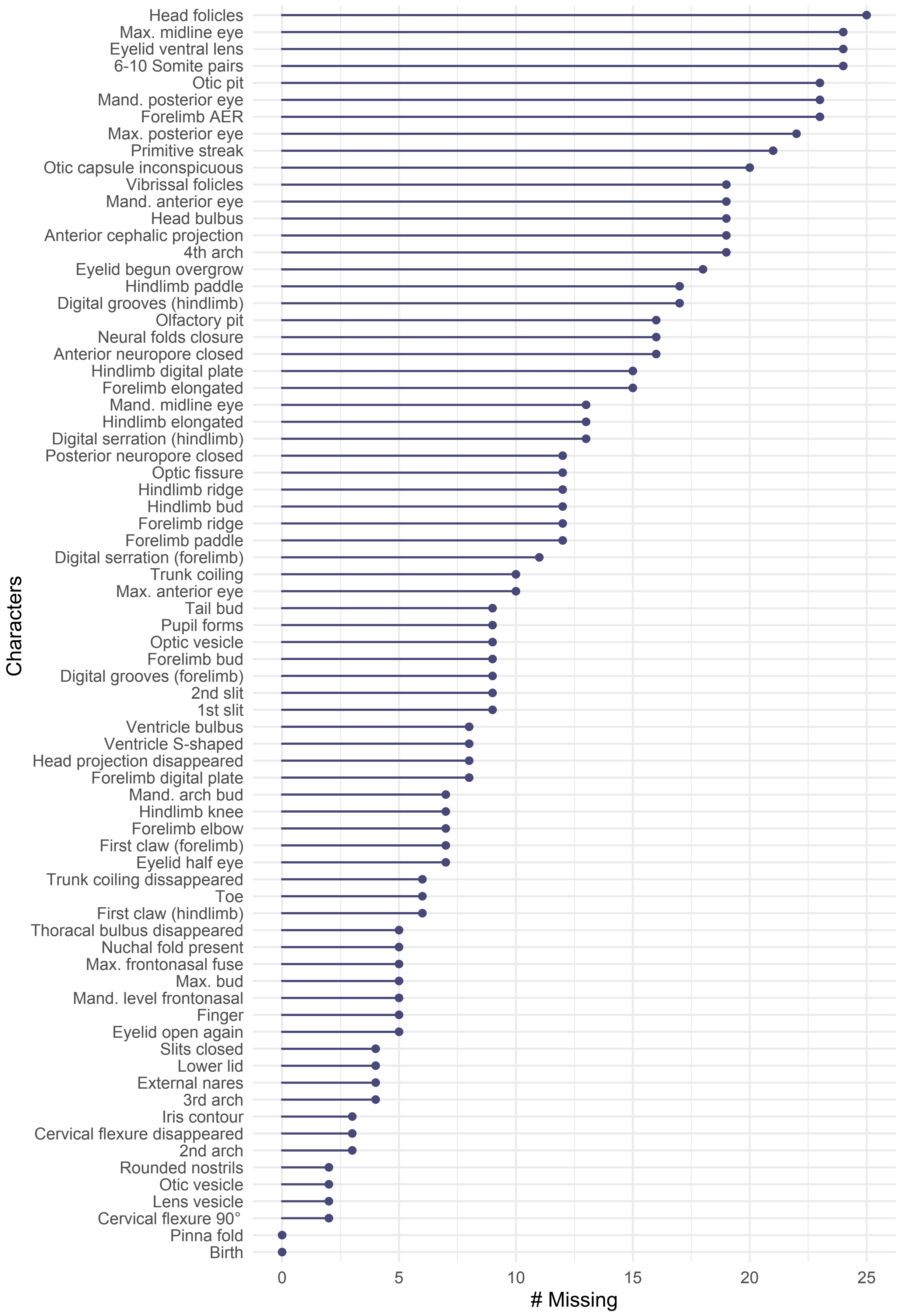

Figure S7. Missing character values, based on a reduced data set of 74 characters across 51 placental mammal species (see Supplementary File 1). The overall missing data rate for this data set was 21%.

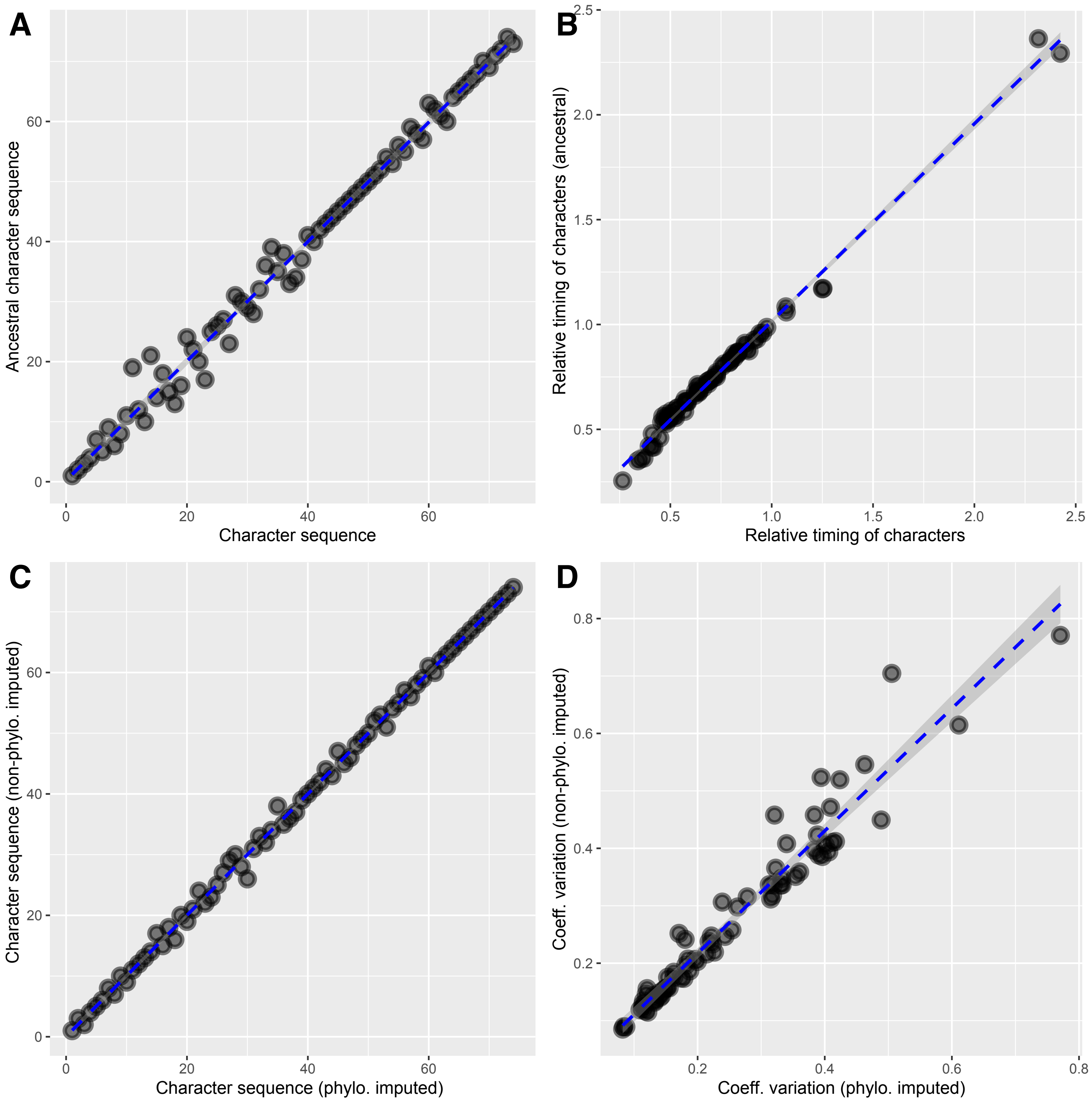

Figure S8. Diagnostic plots displaying correspondence (Spearman's $\rho = 0.99$) between the rank-based character sequence in extant placental mammals versus the reconstructed ancestral sequence (**A**). Means for relative timing of characters were also strongly concordant with reconstructed ancestral values (**B**; $r = 0.99$). Character sequences based on non-phylogenetically imputed versus phylogenetically imputed data sets were tightly correlated (**C**; Spearman's $\rho = 0.99$). Coefficients of variation based on non-phylogenetically imputed versus phylogenetically imputed data sets were also congruent (**D**; $r = 0.97$), though the relationship was moderate for extreme values representing fetal and perinatal (including birth) characters (see Table S2 below).

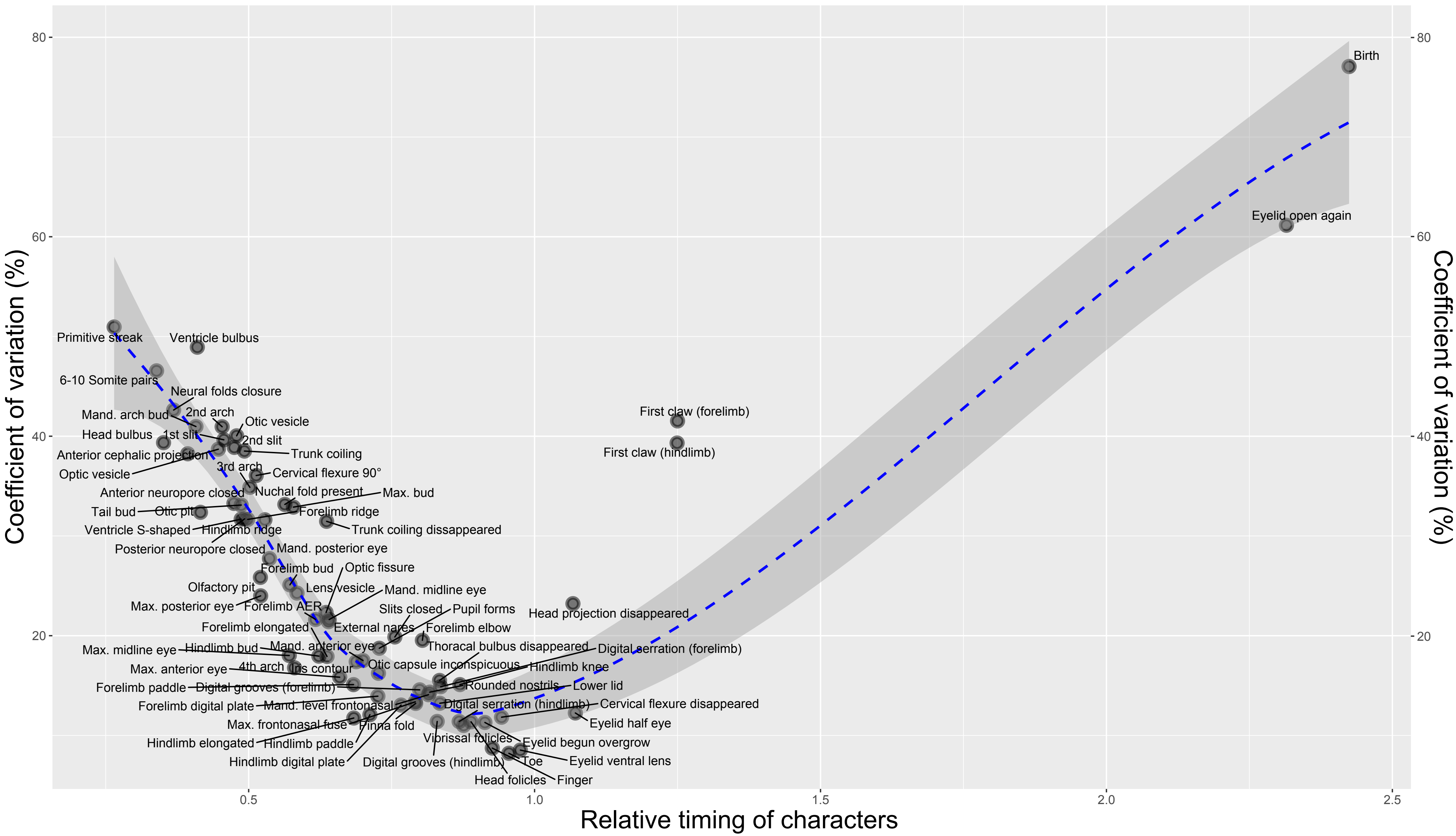

Figure S9. The coefficient of variation is plotted against relative timing scores; based on a character data set that included phylogenetically imputed missing values for 51 placental mammal species. Locally estimated scatterplot smoothing was applied to the data to visualize the mean trend (blue dashed line).

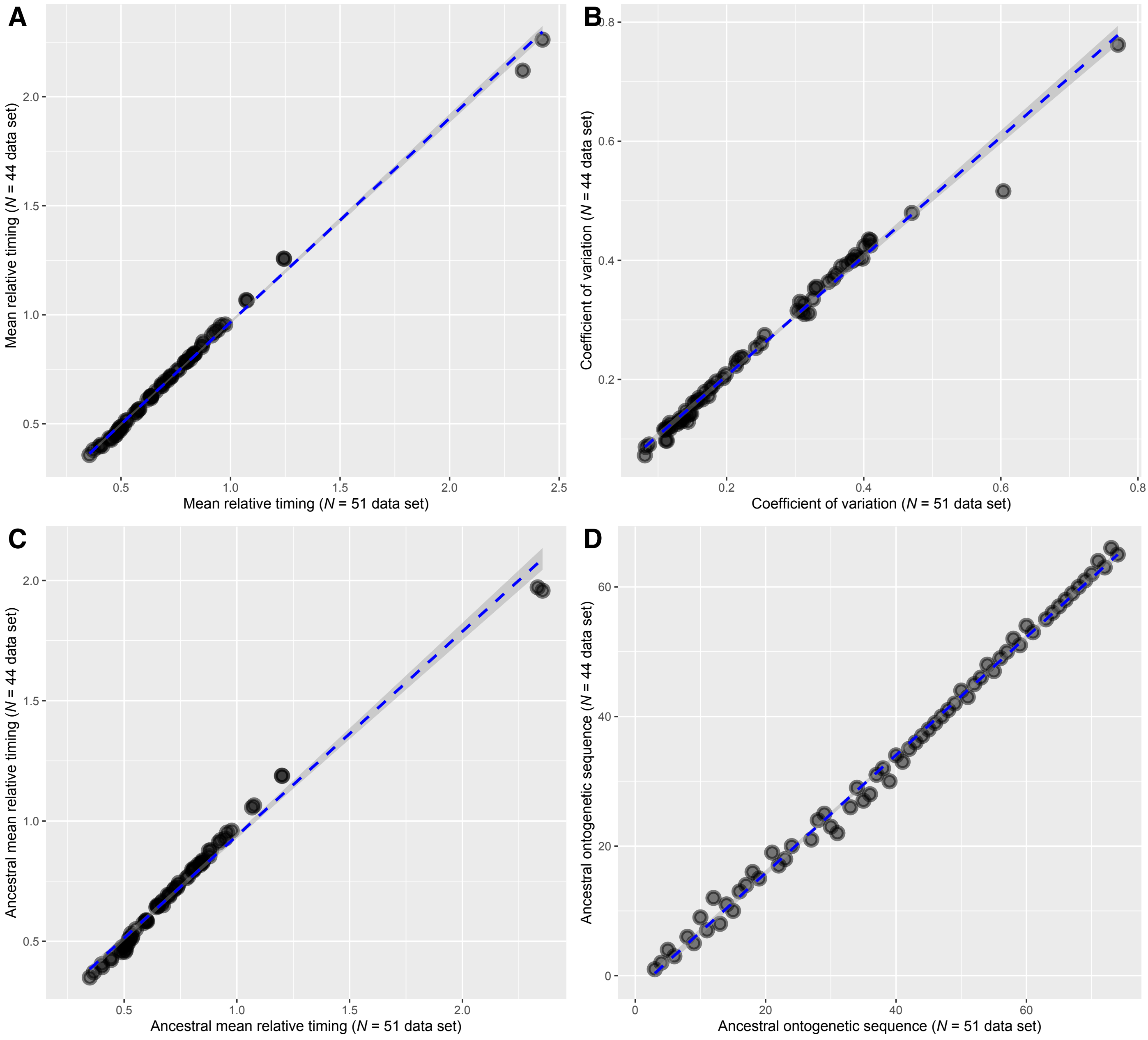

Figure S10. Diagnostic plots displaying correspondence between the mean relative timing for developmental characters in 44-species versus 51-species data sets with 15% and 21% missing data rates, respectively (**A;** Pearson's $r = 0.99$). The coefficients of variation calculated for these data sets were, with few exceptions, highly similar (**B**; Pearson's $r = 0.99$). Ancestral reconstructions for relative timing scores (**C**; Pearson's $r = 0.99$) and their sequence ranks (**D**; Spearman's $\rho = 0.99$) were strongly congruent.

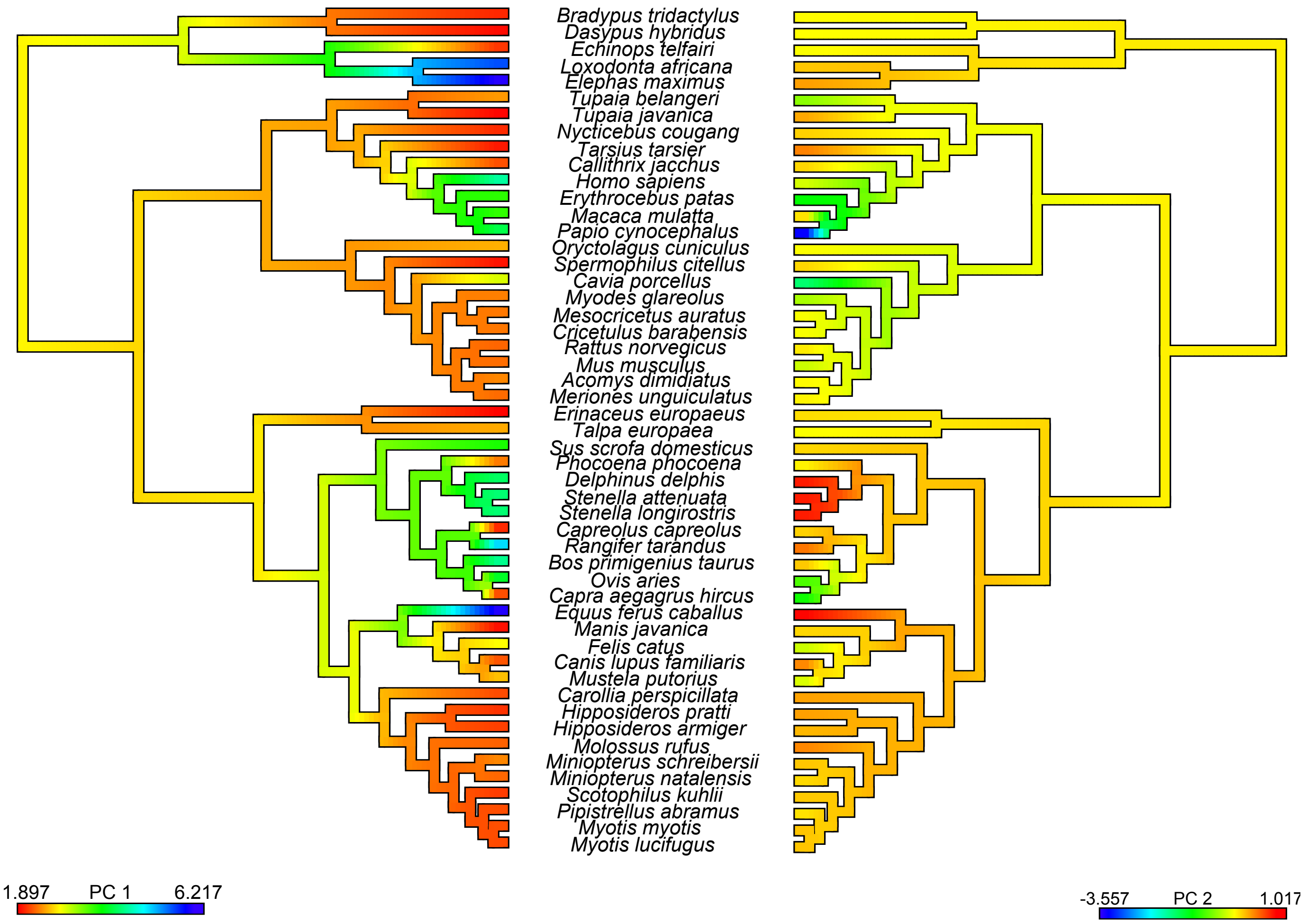

Figure S11. Ancestral state (maximum likelihood) reconstructions of principal components (PC) 1 and 2, on relative timing scores for developmental characters, mapped onto the phylogeny of placental mammals (see Fig. 2 in the main text and Table S1 below) used in this study; states are interpolated along edges as indicated by color gradients.

Figure S12. Scree plot for the phylogenetic principal component (PC) analysis on developmental characters.

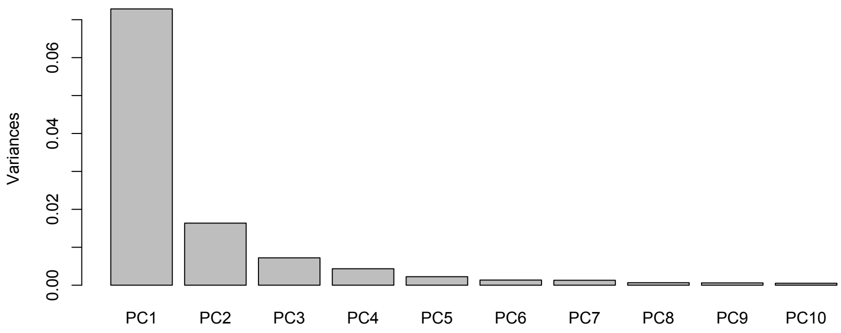

Table S1. Phylogenetic principal component analysis loadings. Variance explained and loadings are listed for the first five principal component (PC) axes. *Abbreviations: Mand. = Mandible; Max. = Maxillary.

Character	PC1 - 65%	PC2 - 15%	PC3 - 6%	PC4 - 4%	PC5 - 2%
Primitive streak	-0.3744	0.0032	-0.1894	-0.0317	0.2896
Neural folds closure	-0.1507	0.2115	-0.5563	-0.1350	0.3227
Anterior neuropore closed	-0.4331	0.0659	-0.5666	-0.1002	0.1981
Posterior neuropore closed	-0.2622	0.1797	-0.6796	-0.2115	0.2025
6-10 Somite pairs	-0.2841	0.1591	-0.5616	-0.1101	0.2904
Head bulbus	-0.1505	0.2317	-0.6085	-0.1002	0.2979
Anterior cephalic projection	-0.1547	0.1208	-0.6178	-0.0405	0.3386
Head projection disappeared	-0.1957	-0.0057	-0.0596	-0.2491	-0.2499
Olfactory pit	0.0153	0.2375	-0.7090	-0.2135	0.0948
External nares	-0.0956	0.1955	-0.6329	-0.3652	-0.0454
Rounded nostrils	-0.0318	0.0525	-0.5675	-0.3650	-0.3616
Otic pit	-0.1258	0.2201	-0.5693	-0.0259	0.2135
Otic vesicle	-0.1425	0.1751	-0.7257	-0.3693	0.2496
Otic capsule inconspicuous	-0.0293	0.2187	-0.4686	-0.3670	-0.3520
Pinna fold	0.1171	0.1374	-0.5475	-0.2229	-0.4580
Optic vesicle	-0.0381	0.2134	-0.7151	-0.0977	0.3135
Lens vesicle	0.0054	0.1968	-0.7212	-0.4402	-0.0275
Optic fissure	-0.0044	0.0623	-0.5935	-0.5066	-0.1789
Iris contour	0.1268	0.1135	-0.7216	-0.2847	-0.3641
Pupil forms	0.0228	0.1613	-0.5377	-0.2016	-0.2382
Ventricle bulbus	-0.2186	0.0787	-0.7104	-0.1513	0.4699
Thoracal bulbus disappeared	0.0756	0.1305	-0.4205	-0.2217	-0.5836
Ventricle S-shaped	-0.1319	0.1920	-0.6523	-0.1102	0.3307
Tail bud	0.0305	0.2174	-0.6937	-0.2422	0.2981
Forelimb ridge	-0.0113	0.1469	-0.6854	-0.1913	0.4215
Hindlimb ridge	-0.0364	0.1698	-0.6710	-0.4054	0.2898
Forelimb bud	-0.1005	0.0793	-0.5561	-0.4171	0.2612
Hindlimb bud	0.0629	0.1435	-0.5476	-0.2997	-0.0887
Forelimb elongated	0.0827	-0.0548	-0.4751	-0.3789	-0.0247
Hindlimb elongated	0.1700	0.1336	-0.5681	-0.2969	0.0461
Forelimb AER	-0.0761	0.1471	-0.4834	-0.4388	-0.0201
Forelimb elbow	0.0386	0.1613	-0.3793	-0.3005	-0.3261

Hindlimb knee	0.1153	0.1933	-0.3652	-0.2208	-0.3508
Forelimb paddle	-0.0487	0.1509	-0.6316	-0.3146	0.0008
Hindlimb paddle	-0.0755	0.1695	-0.4876	-0.3800	0.2077
Forelimb digital plate	-0.1651	0.0060	-0.5309	-0.4220	-0.3142
Hindlimb digital plate	-0.2016	-0.0693	-0.5484	-0.3907	-0.3821
Digital grooves (forelimb)	-0.1226	0.0276	-0.5010	-0.4079	-0.4720
Digital grooves (hindlimb)	-0.1583	0.0790	-0.3921	-0.3557	-0.4802
Digital serration (forelimb)	-0.1954	0.0014	-0.3586	-0.4929	-0.4848
Digital serration (hindlimb)	-0.2824	0.1675	-0.3009	-0.3497	-0.4839
Finger	-0.0343	0.1401	-0.4152	-0.2684	-0.4620
Toe	-0.0739	0.2118	-0.4315	-0.2895	-0.5049
First claw (forelimb)	-0.2717	0.9506	0.1396	-0.0467	0.0050
First claw (hindlimb)	-0.2705	0.9509	0.1406	-0.0395	0.0042
Birth	-0.9726	-0.1277	0.1227	-0.1490	0.0204
Max. bud	0.0416	0.0805	-0.2588	0.1989	0.5204
Max. posterior eye	0.1574	0.2347	-0.5534	-0.1284	0.2512
Max. midline eye	0.0365	0.1970	-0.7126	0.0014	0.0791
Max. anterior eye	0.0801	0.1519	-0.6241	-0.3529	0.1687
Max. frontonasal fuse	0.2094	0.3366	-0.4682	-0.3637	-0.4616
Mand. arch bud	-0.1680	0.1477	-0.5824	-0.2082	0.4048
Mand. posterior eye	0.1393	0.1419	-0.6018	-0.2215	0.2834
Mand. midline eye	-0.1113	-0.1467	-0.5742	-0.3636	-0.2004
Mand. anterior eye	-0.0494	0.0035	-0.3562	-0.2927	-0.1490
Mand. level frontonasal	-0.1760	-0.0122	-0.1328	-0.3649	-0.0614
2nd arch	-0.1840	0.0702	-0.7543	-0.2667	0.4270
3rd arch	-0.1346	0.1576	-0.7845	-0.3485	0.2806
4th arch	0.1489	0.1977	-0.7716	0.2264	0.1017
1st slit	-0.2164	0.1311	-0.6503	-0.2504	0.4686
2nd slit	-0.1370	0.1507	-0.6578	-0.2498	0.4411
Slits closed	0.2171	0.3325	-0.6379	-0.0277	-0.4175
Cervical flexure 90°	-0.0406	0.1574	-0.7512	-0.3677	0.1952
Cervical flexure disappeared	-0.1419	0.1059	-0.3547	-0.1354	-0.6357
Nuchal fold present	-0.0005	0.1142	-0.4945	-0.4568	0.0804
Lower lid	0.1119	0.0956	-0.4009	-0.2779	-0.5580
Eyelid begun overgrow	-0.0010	0.0043	-0.2469	-0.0213	-0.5189
Eyelid ventral lens	-0.0134	-0.1343	-0.1203	-0.2723	-0.4783
Eyelid half eye	-0.2083	-0.2502	-0.1352	-0.3093	-0.4822
Eyelid open again	-0.9508	0.0453	-0.1902	0.2369	-0.0359
Head follicles	-0.0620	0.1439	-0.3061	-0.2942	-0.4051
Vibrissal follicles	-0.0022	0.1422	-0.2299	-0.3077	-0.5722
Trunk coiling	0.1117	0.1760	-0.4504	-0.2252	0.2739
Trunk coiling disappeared	0.1920	0.1818	-0.4205	-0.2965	-0.0355

Table S2. Rates of evolution and reconstructed ancestral timing for 74 developmental characters in 51 placental mammals. Characters are ranked in ascending order according to the ancestral character sequence and are compared to a character sequence based on a ranking of character means of extant species; see standard deviations (SD) and coefficients of variation (CV). *Abbreviations: Mand. = Mandible; Max. = Maxillary.

Character*	Rate of evolution (σ^2)	Ancestral relative timing (reconstructed)	Ancestral character sequence (ranks)	Character sequence (ranks)	Character relative timing (mean)	SD	CV
Primitive streak	0.0040	0.261	1	1	0.265	0.135	0.510
6-10 Somite pairs	0.0043	0.347	2	2	0.339	0.158	0.466
Head bulbus	0.0036	0.353	3	3	0.351	0.138	0.394
Neural folds closure	0.0023	0.366	4	4	0.369	0.157	0.426
Otic pit	0.0015	0.407	5	8	0.416	0.135	0.324
Mand. arch bud	0.0047	0.409	6	6	0.408	0.167	0.410
Anterior cephalic projection	0.0020	0.412	7	5	0.393	0.150	0.382
Optic vesicle	0.0027	0.450	8	9	0.447	0.173	0.387
Ventricle bulbus	0.0030	0.477	9	7	0.410	0.201	0.489
Otic vesicle	0.0035	0.520	10	14	0.479	0.192	0.400
2nd arch	0.0034	0.520	11	10	0.454	0.186	0.409
1st slit	0.0028	0.529	12	11	0.457	0.181	0.396
Posterior neuropore closed	0.0015	0.536	13	18	0.493	0.156	0.317
Anterior neuropore closed	0.0011	0.536	14	12	0.474	0.158	0.333
Max. posterior eye	0.0008	0.538	15	23	0.521	0.125	0.240
Ventricle S-shaped	0.0018	0.541	16	15	0.487	0.154	0.317
Tail bud	0.0023	0.542	17	16	0.487	0.161	0.331
Olfactory pit	0.0014	0.544	18	22	0.521	0.135	0.258
2nd slit	0.0028	0.544	19	13	0.475	0.185	0.389
Trunk coiling	0.0034	0.546	20	17	0.493	0.190	0.385
Forelimb ridge	0.0020	0.548	21	19	0.498	0.158	0.317
3rd arch	0.0030	0.571	22	20	0.502	0.175	0.349
Cervical flexure 90°	0.0036	0.571	23	21	0.513	0.185	0.361
Max. midline eye	0.0003	0.581	24	27	0.571	0.103	0.180
Hindlimb ridge	0.0023	0.582	25	24	0.528	0.167	0.316
Mand. posterior eye	0.0009	0.591	26	25	0.536	0.149	0.277
Nuchal fold present	0.0035	0.614	27	26	0.563	0.187	0.332
4th arch	0.0003	0.616	28	30	0.580	0.097	0.168
Max. bud	0.0012	0.618	29	29	0.578	0.190	0.329
Lens vesicle	0.0013	0.621	30	31	0.585	0.142	0.243
Forelimb bud	0.0012	0.631	31	28	0.572	0.144	0.251
Forelimb AER	0.0014	0.645	32	32	0.617	0.133	0.216
Hindlimb bud	0.0004	0.668	33	33	0.623	0.112	0.179
Optic fissure	0.0014	0.669	34	34	0.635	0.142	0.223
External nares	0.0008	0.670	35	37	0.640	0.137	0.214
Mand. midline eye	0.0012	0.671	36	38	0.641	0.139	0.216
Max. anterior eye	0.0003	0.687	37	39	0.659	0.104	0.158
Forelimb elongated	0.0005	0.695	38	36	0.637	0.114	0.179
Trunk coiling disappeared	0.0015	0.702	39	35	0.636	0.200	0.315

Hindlimb elongated	0.0002	0.704	40	41	0.684	0.080	0.117
Forelimb paddle	0.0003	0.720	41	40	0.683	0.103	0.151
Iris contour	0.0006	0.725	42	42	0.688	0.120	0.174
Mand. anterior eye	0.0005	0.734	43	43	0.700	0.123	0.175
Hindlimb paddle	0.0002	0.739	44	44	0.712	0.086	0.121
Otic capsule inconspicuous	0.0005	0.740	45	46	0.727	0.118	0.162
Forelimb digital plate	0.0003	0.755	46	45	0.726	0.101	0.139
Pupil forms	0.0004	0.764	47	47	0.728	0.136	0.187
Slits closed	0.0007	0.791	48	48	0.756	0.150	0.199
Hindlimb digital plate	0.0002	0.806	49	49	0.767	0.100	0.130
Max. frontonasal fuse	0.0004	0.816	50	50	0.791	0.106	0.134
Pinna fold	0.0003	0.819	51	51	0.792	0.105	0.132
Digital grooves (forelimb)	0.0004	0.826	52	52	0.799	0.116	0.146
Forelimb elbow	0.0013	0.831	53	53	0.804	0.157	0.195
Mand. level frontonasal	0.0005	0.840	54	54	0.814	0.115	0.141
Digital grooves (hindlimb)	0.0002	0.853	55	56	0.830	0.095	0.114
Rounded nostrils	0.0003	0.859	56	55	0.817	0.117	0.143
Digital serration (forelimb)	0.0004	0.861	57	59	0.836	0.124	0.149
Lower lid	0.0004	0.866	58	58	0.834	0.110	0.132
Thoracal bulbus disappeared	0.0006	0.868	59	57	0.834	0.129	0.155
Vibrissal follicles	0.0003	0.877	60	62	0.876	0.096	0.110
Head follicles	0.0003	0.878	61	63	0.889	0.101	0.114
Digital serration (hindlimb)	0.0002	0.894	62	60	0.868	0.099	0.114
Hindlimb knee	0.0004	0.902	63	61	0.869	0.131	0.151
Eyelid begun overgrow	0.0002	0.923	64	64	0.913	0.103	0.113
Finger	0.0003	0.929	65	65	0.926	0.081	0.087
Cervical flexure disappeared	0.0004	0.956	66	66	0.942	0.111	0.118
Toe	0.0002	0.959	67	67	0.955	0.078	0.082
Eyelid ventral lens	0.0001	0.982	68	68	0.975	0.083	0.085
Eyelid half eye	0.0004	1.062	69	70	1.071	0.131	0.123
Head projection disappeared	0.0013	1.092	70	69	1.067	0.248	0.232
First claw (hindlimb)	0.0020	1.178	71	71	1.249	0.491	0.393
First claw (forelimb)	0.0021	1.180	72	72	1.250	0.519	0.415
Birth	0.0060	2.322	73	74	2.424	1.868	0.771
Eyelid open again	0.0046	2.378	74	73	2.315	1.415	0.612

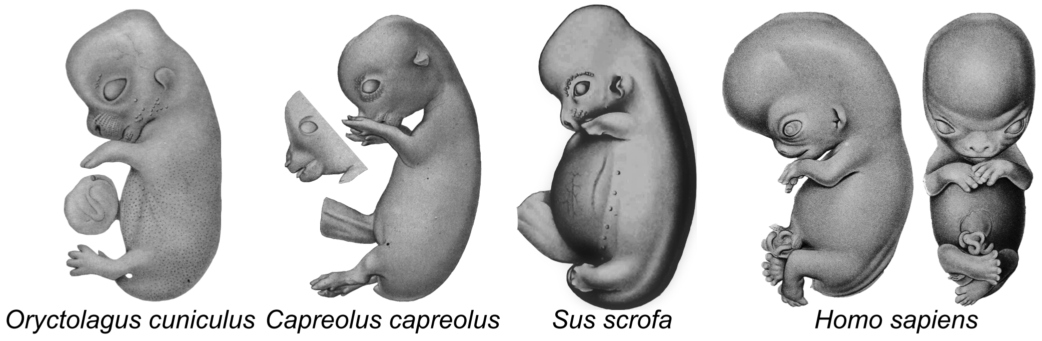

Figure S13. Representative embryos displaying characters associated with the embryo-to-fetus transition in placental mammals (see Tables S1-2). Images are modified from [2-5].

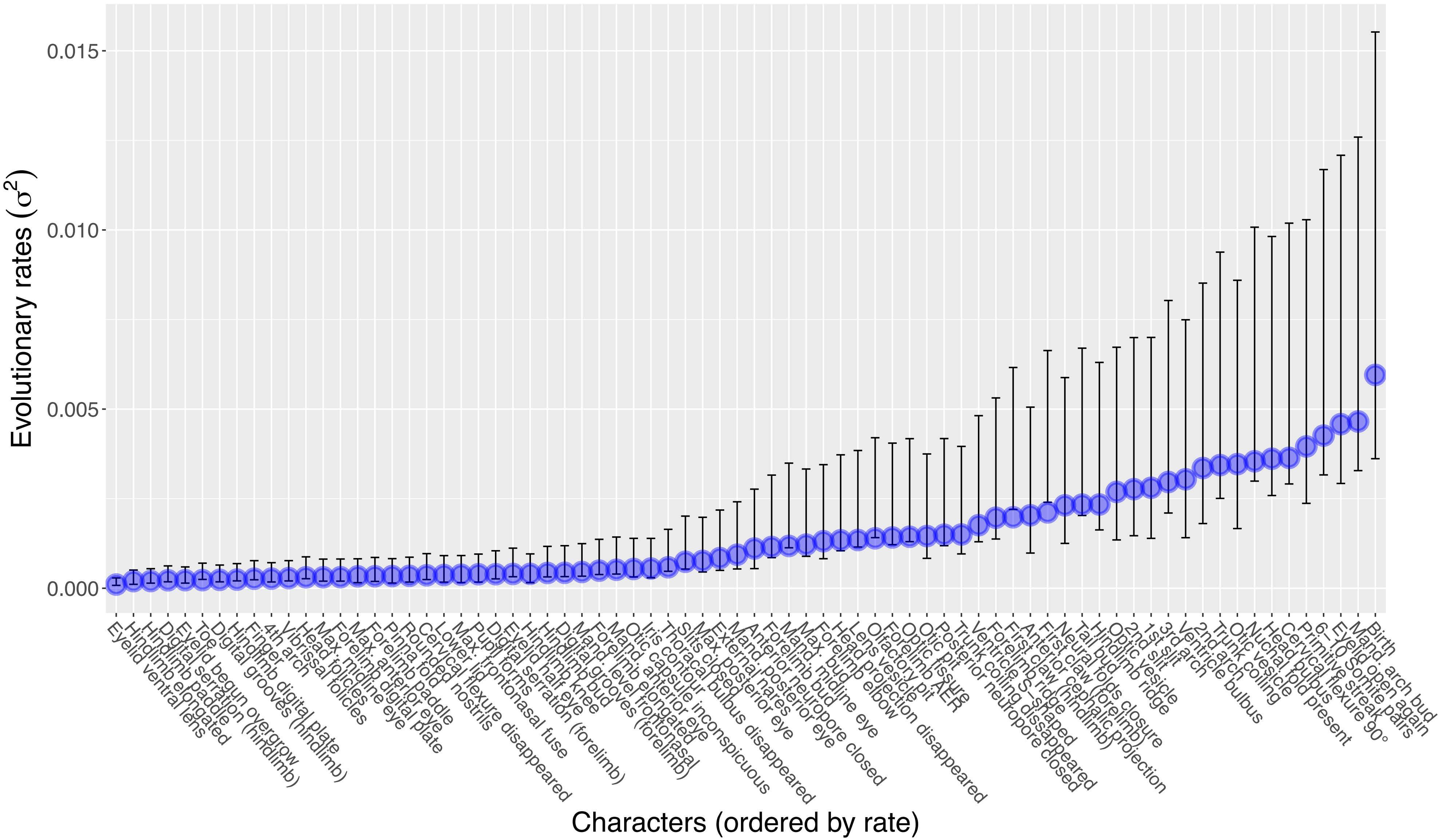

Figure S14. Evolutionary rates for developmental characters in placental mammals. Characters are in ascending order according to evolutionary rates (range bars = 95% confidence intervals).

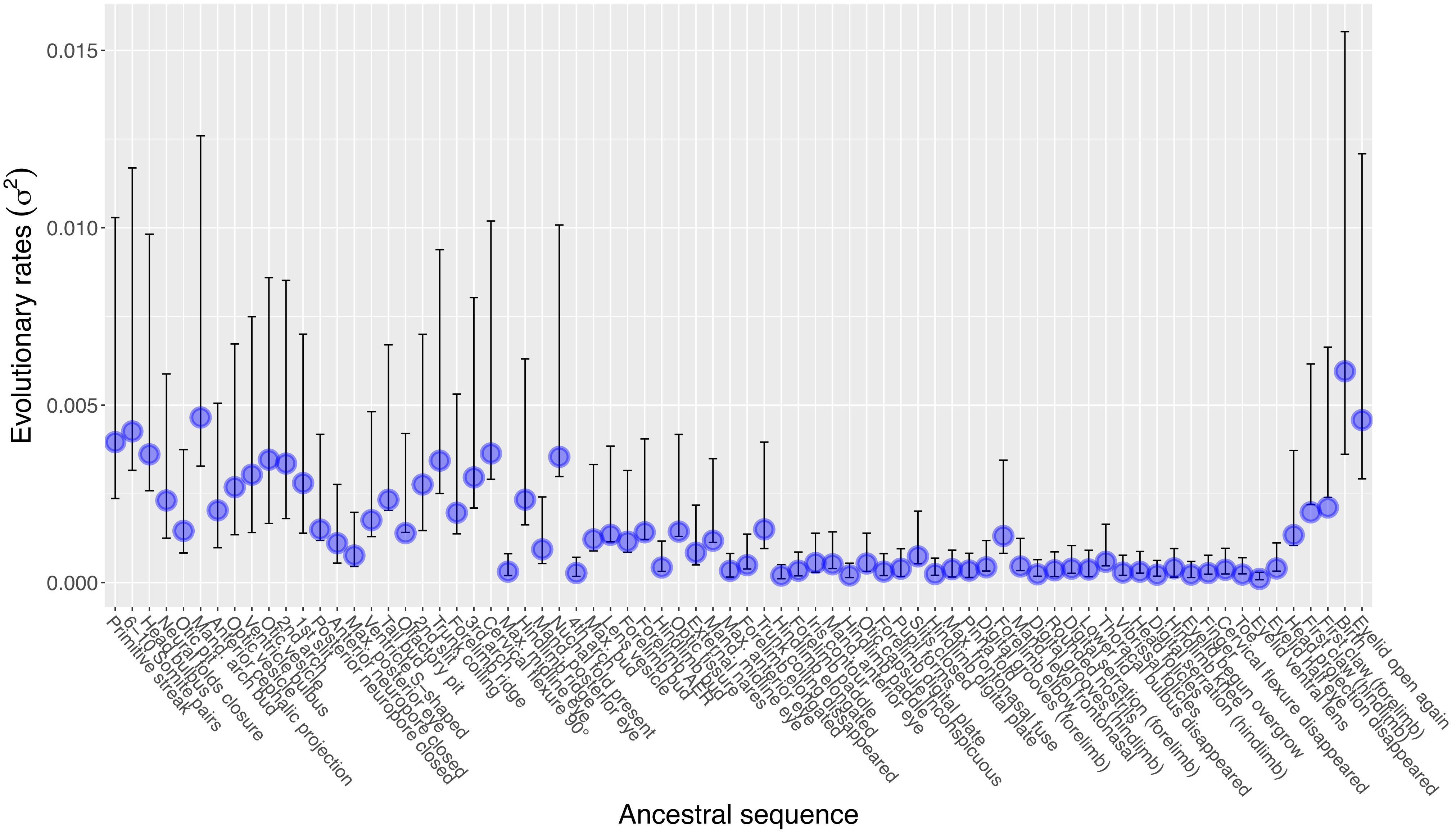

Figure S15. Evolutionary rates (range bars = 95% confidence intervals) for developmental characters. Characters are listed according to the order of the reconstructed ancestral developmental sequence for placental mammals.

3. Supplementary References

1. Werneburg I., Laurin M., Koyabu D., Sánchez-Villagra M.R. 2016 Evolution of organogenesis and the origin of altriciality in mammals. *Evol Dev* **18**(4), 229-244. (doi:10.1111/ede.12194).
2. Keibel F. 1897 Normentafel zur Entwicklungsgeschichte des Schweines (*Sus scrofa domesticus*). In *Normentafeln zur Entwicklungsgeschichte der Wirbeltiere* (ed. Keibel F.). Jena, Verlag von Gustav Fischer.
3. Minot C.S., Taylor E. 1905 Normal plates of the development of the rabbit (*Lepus cuniculus* L.). In *Normentafeln zur Entwicklungsgeschichte der Wirbeltiere* (ed. Keibel F.). Jena, Gustav Fischer Verlag.
4. Sakurai T. 1906 Normentafel zur Entwicklungsgeschichte des Rehes (*Cervus capreolus*). In *Normentafeln zur Entwicklungsgeschichte der Wirbeltiere* (ed. Keibel F.). Jena, Verlag von Gustav Fischer.
5. Keibel F., Elze C. 1908 Normentafel zur Entwicklungsgeschichte des Menschen. In *Normentafeln zur Entwicklungsgeschichte der Wirbeltiere* (ed. Keibel F.). Jena, Verlag von Gustav Fischer.
6. Werneburg I. 2009 A standard system to study vertebrate embryos. *PLoS One* **4**(6), e5887. (doi:10.1371/journal.pone.0005887).
7. Kumar S., Stecher G., Suleski M., Hedges S.B. 2017 TimeTree: A Resource for Timelines, Timetrees, and Divergence Times. *Mol Biol Evol* **34**(7), 1812-1819. (doi:10.1093/molbev/msx116).
8. Standard Event System. 2020 https://en.wikipedia.org/wiki/Standard_Event_System.
9. Werneburg I., Geiger M. 2016 Ontogeny of domestic dogs and the developmental foundations of carnivoran domestication. *Journal of Mammalian Evolution* **24**(3), 323-343. (doi:10.1007/s10914-016-9346-9).
10. Germain D., Laurin M. 2009 Evolution of ossification sequences in salamanders and urodele origins assessed through event-pairing and new methods. *Evolution & Development* **11**(2), 170-190. (doi:10.1111/j.1525-142X.2009.00318.x).
11. Laurin M., Germain D. 2011 Developmental characters in phylogenetic inference and their absolute timing information. *Syst Biol* **60**(5), 630-644. (doi:10.1093/sysbio/syr024).
12. Madley-Dowd P., Hughes R., Tilling K., Heron J. 2019 The proportion of missing data should not be used to guide decisions on multiple imputation. *J Clin Epidemiol* **110**, 63-73. (doi:10.1016/j.jclinepi.2019.02.016).
13. Josse J., Husson F. 2016 missMDA: A package for handling missing values in multivariate data analysis. *Journal of Statistical Software* **70**(1), 1-31. (doi:10.18637/jss.v070.i01).
14. Wickham H. 2016 *ggplot2: Elegant graphics for data analysis*. New York, Springer-Verlag.
15. Adams D.C. 2012 Comparing evolutionary rates for different phenotypic traits on a phylogeny using likelihood. *Systematic Biology* **62**(2), 181-192. (doi:10.1093/sysbio/sys083).
16. Goolsby E.W. 2015 Phylogenetic comparative methods for evaluating the evolutionary history of function-valued traits. *Systematic Biology* **64**(4), 568-578. (doi:10.1093/sysbio/syv012).
17. Goolsby E.W., Bruggeman J., Ané C., Fitzjohn R. 2017 Rphylopars: fast multivariate phylogenetic comparative methods for missing data and within-species variation. *Methods in Ecology and Evolution* **8**(1), 22-27. (doi:10.1111/2041-210x.12612).
18. Bruggeman J., Heringa J., Brandt B.W. 2009 PhyloPars: estimation of missing parameter values using phylogeny. *Nucleic Acids Res* **37**(Web Server issue), W179-184. (doi:10.1093/nar/gkp370).

19. Losos J.B. 2011 Seeing the forest for the trees: the limitations of phylogenies in comparative biology. (American Society of Naturalists Address). *Am Nat* **177**(6), 709-727. (doi:10.1086/660020).
20. Adams D.C. 2014 A generalized K statistic for estimating phylogenetic signal from shape and other high-dimensional multivariate data. *Systematic Biology* **63**(5), 685-697. (doi:10.1093/sysbio/syu030).
21. Adams D.C., Otárola-Castillo E. 2013 geomorph: a R pakcage for the collection and analysis of geometric shape data *Methods in Ecology and Evolution* **4**, 393-399.
22. Revell L.J. 2012 phytools: an R package for phylogenetic comparative biology (and other things). *Methods in Ecology and Evolution* **3**(2), 217-223. (doi:10.1111/j.2041-210X.2011.00169.x).
23. Revell L.J. 2013 Two new graphical methods for mapping trait evolution on phylogenies. *Methods in Ecology and Evolution* **4**(8), 754-759. (doi:10.1111/2041-210x.12066).
24. Werneburg I., Spiekman S.N.F. 2018 Mammalian embryology and organogenesis. In *Handbook of Zoology: Mammalian Evolution, Diversity and Systematics* (eds. Zachos F., Asher R.). Berlin, De Gruyter.
