# Supplementary material for: An irregular hourglass pattern describes the tempo of phenotypic development in placental mammal evolution
Source: Biol Lett. 2020 May 13;16(5):20200087. doi: 10.1098/rsbl.2020.0087 (PMC7280037; doi:10.1098/rsbl.2020.0087)
Supplement: Data [file rsbl20200087supp2.pdf]

Supplementary File 1

An irregular hourglass pattern describes the tempo of phenotypic development in placental mammal evolution

G.A. Cordero, M.R. Sánchez-Villagra, and I. Werneburg

Data matrix of absolute time, relative time, and ranks of developmental characters: Data for the 51 placental mammal species that were used the final analyses: 74 characters + the reference "Mand. occlusion point" character used to scale data. Each data point was assigned an internal tracking ID (IW #) to be matched to the corresponding source from which data was extracted (see the subsequent table below). The data (ranks and absolute time [Abs. time]) were originally extracted and transformed by Werneburg et al. (2016). Full details on data transformation and character definition can be found at:

Werneburg, I., Laurin, M., Koyabu, D., & Sánchez-Villagra, M. R. (2016). Evolution of organogenesis and the origin of altriciality in mammals. *Evol Dev, 18*(4), 229-244.

Species ID	IW1444			IW1290			IW1297		IW1299			IW1306			IW1276	
Species	*Acomys dimidiatus*			*Bos primigenius taurus*			*Bradypus tridactylus*		*Callithrix jacchus*			*Canis lupus familiaris*			*Capra aegagrus hircus*	
Character name	Rank	Abs. time (d)	Scaled	Rank	Abs. time (d)	Scaled	Rank	Scaled	Rank	Abs. time (d)	Scaled	Rank	Abs. time (d)	Scaled	Rank	Scaled
Primitive streak				1	19.5	0.375	1	0.200	1	26.5	0.264	5	15	0.333	3	0.214
Neural folds closure				2	20.5	0.394	2	0.400	4	56	0.557	10	15.5	0.344	7	0.500
Anterior neuropore closed				4	22.7	0.437	4	0.800	5	57	0.567	11	15.6	0.347		
Posterior neuropore closed							4	0.800				22	18.8	0.418		
6-10 somite pairs				2	20.5	0.394	2	0.400				15	16	0.356		
Head bulbus				3	21.6	0.415	2	0.400	2	45.5	0.453	16	16.4	0.364	7	0.500
Anterior cephalic				3	21.6	0.415	3	0.600				18	17.2	0.382		
Head projection	10	26.18	1.206	30	56.5	1.087			15	100.5	1.000	34	28.5	0.633		
Olfactory pit	1	13.875	0.639	4	22.7	0.437			10	64.5	0.642	25	23	0.511		
External nares	3	15.21	0.701	12	33	0.635	4	0.800	11	68	0.677	31	26	0.578	12	0.857
Surrounded nostrils	6	21.71	1.000	19	36.8	0.708	5	1.000	14	80	0.796	34	28.5	0.633	13	0.929
Otic pit				3	21.6	0.415						19	17.6	0.391		
Otic vesicle	2	15.21	0.701	4	22.7	0.437	4	0.800	5	57	0.567	20	18	0.400	9	0.643
Otic capsule inconspicuous	3	17.2	0.792									31	26	0.578		
Pinna fold	4	18.75	0.864	19	36.8	0.708	5	1.000	11	68	0.677	34	28.5	0.633	13	0.929
Optic vesicle				3	21.6	0.415			5	57	0.567	24	19.6	0.436	11	0.786
Lens vesicle	1	13.875	0.639	7	26	0.500	4	0.800	8	61.25	0.609	30	25	0.556	11	0.786
Optic fissure				13	34	0.654			10	64.5	0.642	35	29	0.644		
Contour lens/iris	3	17.2	0.792	18	36.6	0.704	5	1.000	10	64.5	0.642	31	26	0.578	12	0.857
Pupil forms	3	17.2	0.792	20	37	0.712			11	68	0.677	29	24	0.533		
Ventricle bulbus				3	21.6	0.415	4	0.800	4	56	0.557	21	18.4	0.409	8	0.571
Thoracal bulbus	5	19.54	0.900	25	45.5	0.875	5	1.000	14	80	0.796	34	28.5	0.633	13	0.929
Ventricle S-shaped				3	21.6	0.415	4	0.800	5	57	0.567	22	18.8	0.418	8	0.571
Tail bud				5	23.8	0.458	4	0.800	6	58.75	0.585	24	19.6	0.436	9	0.643
Forelimb ridge				6	24.9	0.479	4	0.800	6	58.75	0.585				9	0.643
Hind limb ridge	1	13.875	0.639	6	24.9	0.479			7	60.5	0.602	28	23	0.511	9	0.643
Forelimb bud	1	13.875	0.639	9	28	0.538										
Hind limb bud	2	15.21	0.701	11	30.5	0.587			9	62	0.617					
Forelimb elongated				12	33	0.635			9	62	0.617	28	23	0.511		
Hindlimb elongated				14	35	0.673			10	64.5	0.642	30	25	0.556	12	0.857
Forelimb AER				14	35	0.673						29	24	0.533		
Forelimb elbow	5	19.54	0.900	18	36.6	0.704			12	71.5	0.711				12	0.857
Hindlimb knee	5	19.54	0.900	17	36.4	0.700			15	100.5	1.000				12	0.857
Forelimb paddle				14	35	0.673			10	64.5	0.642	30	25	0.556	12	0.857
Hindlimb paddle	3	17.2	0.792	16	36	0.692			11	68	0.677					
Forelimb digital plate	3	17.2	0.792	16	36	0.692			11	68	0.677	33	28	0.622		
Hindlimb digital plate				17	36.4	0.700			12	71.5	0.711					
Digital grooves (forelimb)				19	36.8	0.708			12	71.5	0.711	34	28.5	0.633		
Digital grooves (hindlimb)				23	40.5	0.779			13	75	0.746					
digital serration (forelimb)	4	18.75	0.864	20	37	0.712			13	75	0.746					
Digital serration	4	18.75	0.864						14	80	0.796				13	0.929
Finger	6	21.71	1.000	23	40.5	0.779	5	1.000	14	80	0.796	35	29	0.644	13	0.929
Toe	7	22.67	1.044	24	44.5	0.856	5	1.000	15	100.5	1.000	35	29	0.644	14	1.000
First claw (forelimb)	8	23.625	1.088	35	80	1.538	5	1.000	15	100.5	1.000	39	35	0.778		
First claw (hind limb)	8	23.625	1.088	35	80	1.538	5	1.000	15	100.5	1.000	39	35	0.778		
Birth	19	37.5	1.727	42	284	5.462	6	1.200	17	141.5	1.408	44	60	1.333	15	1.071
Max. bud	2	15.21	0.701	6	24.9	0.479	4	0.800	11	68	0.677	28	23	0.511	9	0.643
Max. posterior eye				6	24.9	0.479						28	23	0.511	11	0.786
Max. midline eye	2	15.21	0.701									30	25	0.556		
Max. anterior eye	3	17.2	0.792	13	34	0.654						33	28	0.622	12	0.857
Max. frontonasal fuse	6	21.71	1.000	17	36.4	0.700	5	1.000				34	28.5	0.633	13	0.929
Mand. arch bud	1	13.875	0.639	3	21.6	0.415	2	0.400	5	57	0.567	16	16.4	0.364	7	0.500
Mand. posterior eye				6	24.9	0.479	4	0.800				30	25	0.556	11	0.786
Mand. midline eye	2	15.21	0.701	13	34	0.654										
Mand. anterior eye				17	36.4	0.700									12	0.857
Mand. level frontonasal	5	19.54	0.900	25	45.5	0.875						34	28.5	0.633	13	0.929
Mand. occlusion point	6	21.71	1.000	28	52	1.000	5	1.000	15	100.5	1.000	41	45	1.000	14	1.000

Species ID	IW1307		IW1302			IW1286			IW1310			IW1251		IW1285		
Species	*Capreolus capreolus*		*Carollia perspicillata*			*Cavia porcellus*			*Cricetulus barabensis*			*Dasypus hybridus*		*Delphinus delphis*		
Character name	Rank	Scaled	Rank	Abs. time (d)	Scaled	Rank	Abs. time (d)	Scaled	Rank	Abs. time (d)	Scaled	Rank	Scaled	Rank	Abs. time (d)	Scaled
Primitive streak	3	0.083	6	16.7	0.239	3	13.47	0.481				12	0.348			
Neural folds closure	8	0.222	10	30	0.429	10	15	0.536	2	10.75	0.677	16	0.464	1	12.75	0.280
Anterior neuropore closed	13	0.361	11	33.3	0.476	16	16.26	0.581	3	10.875	0.685	20	0.580			
Posterior neuropore closed	21	0.583	12	36.7	0.524	15	16	0.571	5	11.75	0.740	20	0.580	3	13.75	0.302
6-10 somite pairs						9	14.84	0.530				19	0.551	2	13.75	0.302
Head bulbus	10	0.278							1	10	0.630	19	0.551	2	13.25	0.291
Anterior cephalic	16	0.444				8	14.24	0.509	2	10.75	0.677	20	0.580	3	13.75	0.302
Head projection	36	1.000	19	65	0.929	9	29	1.036	13	18	1.134	35	1.014	13	70	1.538
Olfactory pit	24	0.667				21	18.19	0.650	3	10.875	0.685	25	0.725	3	13.75	0.302
External nares	26	0.722	14	44	0.629	23	18.51	0.661	5	11.75	0.740	28	0.812	6	23.5	0.516
Surrounded nostrils	31	0.861	17	54	0.771				8	13	0.819	31	0.899	8	30	0.659
Otic pit	17	0.472	10	30	0.429	13	15.53	0.555	2	10.75	0.677	19	0.551	3	13.75	0.302
Otic vesicle	20	0.556	11	33.3	0.476	15	16	0.571	3	10.875	0.685	20	0.580	4	14	0.308
Otic capsule inconspicuous	30	0.833				28	20.72	0.740	8	13	0.819					
Pinna fold	28	0.778	16	50	0.714	32	23.67	0.845	10	15.125	0.953	29	0.841	8	30	0.659
Optic vesicle	26	0.722	10	30	0.429	14	15.77	0.563	3	10.875	0.685	19	0.551	3	13.75	0.302
Lens vesicle	28	0.778	13	40	0.571	15	16	0.571	4	11.25	0.709	25	0.725	6	23.5	0.516
Optic fissure	28	0.778	16	50	0.714	18	17.53	0.626				30	0.870	8	30	0.659
Contour lens/iris	29	0.806	15	46	0.657	20	17.92	0.640	5	11.75	0.740	31	0.899	7	24.5	0.538
Pupil forms	28	0.778	14	44	0.629				9	14	0.882	30	0.870	8	30	0.659
Ventricle bulbus	15	0.417	10	30	0.429	10	15	0.536	2	10.75	0.677	20	0.580	3	13.75	0.302
Thoracal bulbus	28	0.778	15	46	0.657	33	26.16	0.934	7	12.75	0.803	35	1.014	8	30	0.659
Ventricle S-shaped	17	0.472	11	33.3	0.476	10	15	0.536	3	10.875	0.685			5	14.25	0.313
Tail bud	20	0.556	12	36.7	0.524	16	16.26	0.581	5	11.75	0.740	23	0.667	4	14	0.308
Forelimb ridge	22	0.611	12	36.7	0.524	17	16.5	0.589	3	10.875	0.685	25	0.725	3	13.75	0.302
Hind limb ridge	23	0.639	12	36.7	0.524	21	18.19	0.650	5	11.75	0.740	24	0.696			
Forelimb bud	26	0.722	13	40	0.571	24	19.52	0.697	5	11.75	0.740	27	0.783	6	23.5	0.516
Hind limb bud	26	0.722	14	44	0.629	25	19.7	0.704				27	0.783	6	23.5	0.516
Forelimb elongated	27	0.750	14	44	0.629	28	20.72	0.740	6	12.25	0.772	28	0.812	7	24.5	0.538
Hindlimb elongated	27	0.750				28	20.72	0.740	8	13	0.819	28	0.812	8	30	0.659
Forelimb AER	27	0.750	13	40	0.571									8	30	0.659
Forelimb elbow	28	0.778	17	54	0.771	31	21.64	0.773	7	12.75	0.803	33	0.957			
Hindlimb knee	30	0.833	17	54	0.771	32	23.67	0.845	10	15.125	0.953	34	0.986			
Forelimb paddle	27	0.750							6	12.25	0.772	28	0.812	8	30	0.659
Hindlimb paddle	27	0.750				31	21.64	0.773	8	13	0.819	28	0.812			
Forelimb digital plate	28	0.778	15	46	0.657	31	21.64	0.773	7	12.75	0.803	29	0.841			
Hindlimb digital plate	28	0.778	15	46	0.657	32	23.67	0.845	8	13	0.819	29	0.841			
Digital grooves (forelimb)	30	0.833	16	50	0.714	32	23.67	0.845	10	15.125	0.953	29	0.841	9	38	0.835
Digital grooves (hindlimb)	30	0.833	16	50	0.714	32	23.67	0.845	10	15.125	0.953	31	0.899			
digital serration (forelimb)	29	0.806	17	54	0.771				9	14	0.882	30	0.870	10	45.5	1.000
Digital serration	30	0.833	17	54	0.771	33	26.16	0.934	9	14	0.882	31	0.899			
Finger	34	0.944	18	60	0.857	33	26.16	0.934	11	15.875	1.000	33	0.957			
Toe	34	0.944	18	60	0.857	34	28	1.000	11	15.875	1.000	34	0.986			
First claw (forelimb)	34	0.944	19	65	0.929	35	67	2.393	11	15.875	1.000	35	1.014			
First claw (hind limb)	34	0.944	19	65	0.929	35	67	2.393	11	15.875	1.000	35	1.014			
Birth	38	1.056	25	115	1.643	36	68	2.429	14	20.5	1.291	36	1.043	18	280	6.154
Max. bud	20	0.556	14	44	0.629	17	16.5	0.589	4	11.25	0.709	24	0.696	3	13.75	0.302
Max. posterior eye	20	0.556				18	17.53	0.626	4	11.25	0.709	24	0.696	3	13.75	0.302
Max. midline eye	24	0.667				20	17.92	0.640	5	11.75	0.740	25	0.725	4	14	0.308
Max. anterior eye	26	0.722	14	44	0.629	22	18.31	0.654	6	12.25	0.772	27	0.783	6	23.5	0.516
Max. frontonasal fuse	31	0.861				29	21.03	0.751	8	13	0.819	33	0.957	8	30	0.659
Mand. arch bud	16	0.444	11	33.3	0.476	9	14.84	0.530	2	10.75	0.677	23	0.667	3	13.75	0.302
Mand. posterior eye						17	16.5	0.589	7	12.75	0.803			3	13.75	0.302
Mand. midline eye	24	0.667	15	46	0.657	18	17.53	0.626	8	13	0.819	24	0.696	6	23.5	0.516
Mand. anterior eye	26	0.722				20	17.92	0.640				26	0.754	8	30	0.659
Mand. level frontonasal	28	0.778	16	50	0.714	29	21.03	0.751	9	14	0.882	27	0.783	9	38	0.835
Mand. occlusion point	36	1.000	20	70	1.000	34	28	1.000	11	15.875	1.000	34.5	1.000	10	45.5	1.000

2nd arch	1	13.875	0.639	3	21.6	0.415	4	0.800	5	57	0.567	16	16.4	0.364	8	0.571	17	0.472	11	33.3	0.476	9	14.84	0.530	3	10.875	0.685	24	0.696	3	13.75	0.302
3rd arch	1	13.875	0.639	5	23.8	0.458	4	0.800	7	60.5	0.602	19	17.6	0.391	8	0.571	20	0.556	12	36.7	0.524	17	16.5	0.589	4	11.25	0.709	24	0.696	3	13.75	0.302
4th arch				6	24.9	0.479	4	0.800	7	60.5	0.602	28	23	0.511	9	0.643	21	0.583				17	16.5	0.589				25	0.725	4	14	0.308
1st slit				3	21.6	0.415	4	0.800	5	57	0.567	19	17.6	0.391	7	0.500	17	0.472	13	40	0.571	9	14.84	0.530	3	10.875	0.685			3	13.75	0.302
2nd slit				3	21.6	0.415	4	0.800	7	60.5	0.602	19	17.6	0.391	8	0.571	17	0.472	15	46	0.657	13	15.53	0.555	4	11.25	0.709			3	13.75	0.302
Slits closed	3	17.2	0.792	17	36.4	0.700			13	75	0.746	35	29	0.644	13	0.929	31	0.861	16	50	0.714	32	23.67	0.845	8	13	0.819			5	14.25	0.313
Cervical flexure 90°				4	22.7	0.437	4	0.800	9	62	0.617	24	19.6	0.436	9	0.643	21	0.583	14	44	0.629	13	15.53	0.555	5	11.75	0.740	25	0.725	3	13.75	0.302
Cervical flexure				28	52	1.000	5	1.000	15	100.5	1.000	34	28.5	0.633			32	0.889	17	54	0.771	34	28	1.000	11	15.875	1.000	30	0.870	9	38	0.835
Nuchal fold present	1	13.875	0.639	9	28	0.538			5	57	0.567	24	19.6	0.436	10	0.714	21	0.583	12	36.7	0.524	17	16.5	0.589	5	11.75	0.740	25	0.725	6	23.5	0.516
Lower lid	7	22.67	1.044	24	44.5	0.856	5	1.000	13	75	0.746	35	29	0.644	13	0.929	32	0.889	16	50	0.714	33	26.16	0.934	9	14	0.882	31	0.899	8	30	0.659
Eyelid half eye	8	23.625	1.088	32	60	1.154			15	100.5	1.000	39	35	0.778			37	1.028	20	70	1				8	13	0.819	35	1.0145	12	57	1.253
Eyelid begun overgrow	7	22.67	1.044	26	49.5	0.952						36	30	0.667			34	0.944	17	54	0.771							33	0.957	9	38	0.835
Eyelid ventral lens																	36	1.000										34	0.986	11	46.5	1.022
Eyelid open again	19	37.5	1.727	40	180	3.462	6	1.200	17	141.5	1.408	45	73	1.622	15	1.071	38	1.056	22	80	1.143	36	68	2.429	15	32.5	2.047	36	1.044	15	92.5	2.033
Head folicles	4	18.75	0.864									36	30	0.667	13	0.929	32	0.889				32	23.67	0.845	11	15.875	1.000					
Vibrissal folicles	3	17.2	0.792	25	45.5	0.875						36	30	0.667	14	1.000	33	0.917	16	50	0.714	32	23.67	0.845	10	15.125	0.953					
Trunk coiling	1	13.875	0.639										3	21.600	0.4154	4.000	0.8				24.000	19.6	0.4356	11.000	0.7857	20	0.556	20	0.580	1	12.75	0.280
Trunk coiling dissappeared	3	17.2	0.792										5	23.800	0.4577	5.000	1				30.000	25	0.5556	12.000	0.8571	22	0.611	24	0.696	5	14.25	0.313

IW1271 *Echinops telfairi*			IW1327 *Elephas maximus*			IW1289 *Equus ferus caballus*			IW1312 *Erinaceus europaeus*		IW1282 *Erythrocebus patas*			IW1329 *Felis catus*			IW1303 *Hipposideros armiger*		IW1304 *Hipposideros pratti*		IW1273 *Homo sapiens*			IW1252 *Loxodonta africana*			IW1443 *Macaca mulatta*			IW1287 *Manis javanica*		IW1454 *Meriones unguiculatus*		
Rank	Abs. time (d)	Scaled	Rank	Abs. time (d)	Scaled	Rank	Abs. time (d)	Scaled	Rank	Scaled	Rank	Abs. time (d)	Scaled	Rank	Abs. time (d)	Scaled	Rank	Scaled	Rank	Scaled	Rank	Abs. time (d)	Scaled	Rank	Abs. time (d)	Scaled	Rank	Abs. time (d)	Scaled	Rank	Scaled	Rank	Abs. time (d)	Scaled
						2	15	0.306	2	0.048				6	12.5	0.417	1	0.100	1	0.091	6	13	0.250				1	18	0.409	1	0.048			
						5	19.25	0.393	4	0.095				8	14.5	0.483					10	22	0.423				6	24	0.545			1	12	0.571
			1	77	0.786	13	21.77	0.444	17	0.405				9	16	0.533	2	0.200	2	0.182	15	33	0.635	7	103	1.000	7	26	0.591			2	13	0.619
			1	77	0.786	10	21.44	0.438	17	0.405	1	30	0.600	12	18.5	0.617	3	0.300	3	0.273	12	26	0.500				8	26.5	0.602			2	13	0.619
						8	21.22	0.433	4	0.095				8	14.5	0.483	1	0.100			10	22	0.423				4	21.75	0.494	2	0.095			
						8	21.22	0.433	4	0.095				7	13.5	0.450					6	13	0.250				3	21.375	0.486	4	0.190			
						11	21.55	0.440	5	0.119				9	16	0.533					7	16	0.308				6	24	0.545	8	0.381	1	12	0.571
			2	98	1.000	39	49	1.000	42	1.000	8	50	1.000	16	26.5	0.883	2	0.200			23	56.5	1.087	8	136.9	1.329	16	44	1.000	20	0.952	10	21	1.000
						13	21.77	0.444	26	0.619	2	34	0.680	8	14.5	0.483	5	0.500	5	0.455							8	26.5	0.602	9	0.429	3	14	0.667
1	38.5	0.811	1	77	0.786	19	26.5	0.541	29	0.690	3	37	0.740	13	20	0.667	5	0.500	5	0.455	14	32	0.615	1	58	0.563	10	29	0.659	12	0.571			
4	40.5	0.853	2	98	1.000	36	40	0.816	39	0.929	6	42.5	0.850	15	24	0.800	7	0.700	7	0.636	18	44	0.846	4	92	0.893	14	36	0.818	23	1.095	8	19	0.905
						13	21.77	0.444	5	0.119	1	30	0.600	8	14.5	0.483					9	20	0.385							5	0.238			
1	38.5	0.811				15	22	0.449	21	0.500	2	34	0.680	11	17.5	0.583	2	0.200	2	0.182	12	26	0.500	1	58	0.563	8	26.5	0.602	8	0.381	2	13	0.619
4	40.5	0.853	1	77	0.786				31	0.738				13	20	0.667					13	28	0.538	3	81	0.786	12	32	0.727					
4	40.5	0.853	1	77	0.786	31	35.33	0.721	36	0.857	7	45	0.900	14	22	0.733	7	0.700	7	0.636	18	44	0.846	3	81	0.786	13	34	0.773	19	0.905	7	18	0.857
						15	22	0.449	16	0.381	1	30	0.600	8	14.5	0.483	2	0.200	2	0.182	12	26	0.500	1	58	0.563	7	26	0.591	4	0.190	1	12	0.571
1	38.5	0.811				19	26.5	0.541	29	0.690	2	34	0.680	10	17	0.567	4	0.400	4	0.364	14	32	0.615	2	69.5	0.675	9	27	0.614	11	0.524	4	15	0.714
3	40	0.842				23	28.5	0.582	29	0.690	2	34	0.680	13	20	0.667			5	0.455	15	33	0.635	2	69.5	0.675	10	29	0.659	11	0.524	5	16	0.762
1	38.5	0.811	1	77	0.786	28	33	0.673	29	0.690	3	37	0.740	12	18.5	0.617	6	0.600	6	0.545	15	33	0.635	2	69.5	0.675	12	32	0.727	14	0.667	5	16	0.762
3	40	0.842	1	77	0.786	25	30	0.612	40	0.952	3	37	0.740	13	20	0.667	5	0.500	5	0.455	16	37	0.712				13	34	0.773	17	0.810			
1	38.5	0.811				11	21.55	0.440	4	0.095	1	30	0.600	9	16	0.533	2	0.200	2	0.182	10	22	0.423	1	58	0.563	7	26	0.591	4	0.190	1	12	0.571
5	41	0.863	2	98	1.000	28	33	0.673	32	0.762	7	45	0.900				8	0.800	7	0.636	23	56.5	1.087	4	92	0.893	13	34	0.773	19	0.905	7	18	0.857
1	38.5	0.811				14	21.88	0.447	20	0.476	1	30	0.600	10	17	0.567	5	0.500	5	0.455	11	24	0.462	1	58	0.563	7	26	0.591	9	0.429	2	13	0.619
						10	21.44	0.438	17	0.405	2	34	0.680	9	16	0.533	3	0.300	3	0.273	12	26	0.500	1	58	0.563	6	24	0.545	9	0.429	2	13	0.619
						16	23	0.469	25	0.595	2	34	0.680	11	17.5	0.583	2	0.200			12	26	0.500	1	58	0.563	8	26.5	0.602	9	0.429	2	13	0.619
1	38.5	0.811				16	23	0.469	27	0.643	2	34	0.680	12	18.5	0.617	2	0.200	2	0.182	13	28	0.538				8	26.5	0.602	9	0.429	3	14	0.667
1	38.5	0.811	1	77	0.786	19	26.5	0.541	29	0.690				12	18.5	0.617	3	0.300	3	0.273	13	28	0.538				10	29	0.659	10	0.476	3	14	0.667
2	39.5	0.832				19	26.5	0.541	31	0.738	3	37	0.740				4	0.400	4	0.364	14	32	0.615				10	29	0.659	10	0.476	4	15	0.714
2	39.5	0.832	1	77	0.786	20	27	0.551	29	0.690	3	37	0.740						5	0.455	14	32	0.615				12	32	0.727	11	0.524			
						20	27	0.551	34	0.810	4	38.5	0.770	13	20	0.667			6	0.545	15	33	0.635				12	32	0.727	13	0.619			
1	38.5	0.811				21	27.5	0.561			3	37	0.740	12	18.5	0.617	4	0.400	4	0.364	14	32	0.615							10	0.476			
8	47.5	1.000	2	98	1.000	32	36	0.735			6	42.5	0.850	13	20	0.667	8	0.800	11	1.000	15	33	0.635	2	69.5	0.675	12	32	0.727	11	0.524			
8	47.5	1.000	2	98	1.000	36	40	0.816	42	1.000	7	45	0.900	13	20	0.667	8	0.800	11	1.000	17	41	0.788	4	92	0.893	13	34	0.773	16	0.762			
3	40	0.842	2	98	1.000	27	32	0.653	31	0.738	4	38.5	0.770								15	33	0.635	2	69.5	0.675				10	0.476	4	15	0.714
3	40	0.842	1	77	0.786	27	32	0.653	34	0.810	5	40	0.800						6	0.545	16	37	0.712	3	81	0.786	12	32	0.727	11	0.524	5	16	0.762
3	40	0.842	2	98	1.000	31	35.33	0.721	34	0.810	5	40	0.800	13	20	0.667			6	0.545	16	37	0.712	2	69.5	0.675	13	34	0.773	16	0.762	5	16	0.762
3	40	0.842	2	98	1.000	32	36	0.735	39	0.929	6	42.5	0.850	14	22	0.733			7	0.636	17	41	0.788				14	36	0.818	16	0.762	6	17	0.810
4	40.5	0.853	4	120	1.224				39	0.929	6	42.5	0.850	14	22	0.733					17	41	0.788	2	69.5	0.675	15	36.5	0.830	16	0.762	6	17	0.810
4	40.5	0.853	4	120	1.224				40	0.952	6	42.5	0.850	14	22	0.733					18	44	0.846	3	81	0.786	15	36.5	0.830			7	18	0.857
4	40.5	0.853	4	120	1.224				39	0.929	7	45	0.900	15	24	0.800					18	44	0.846	4	92	0.893	14	36	0.818			7	18	0.857
4	40.5	0.853	4	120	1.224				40	0.952	7	45	0.900	15	24	0.800	8	0.800			20	50.5	0.971				15	36.5	0.830	19	0.905	8	19	0.905
5	41	0.863				36	40	0.816	41	0.976	8	50	1.000	16	26.5	0.883	9	0.900	9	0.818	20	50.5	0.971	4	92	0.893	16	44	1.000	19	0.905	8	19	0.905
6	43.16	0.909				38			41	0.976	8	50	1.000	16	26.5	0.883	9	0.900	9	0.818	21	52	1.000	5	97	0.942	16	44	1.000	20	0.952	10	21	1.000
8	47.5	1.000	6	133	1.357	39	49	1.000	42	1.000				19	41	1.367	10	1.000	10	0.909	25	91	1.750	9	150	1.456	17	53	1.205	22	1.048	9	20	0.952
8	47.5	1.000	6	133	1.357	39	49	1.000	42	1.000				19	41	1.367	10	1.000	10	0.909	25	91	1.750	9	150	1.456	17	53	1.205	22	1.048	10	21	1.000
10	49	1.032	10	660	6.735	48	335	6.837	43	1.024	9	170	3.400	22	62	2.067	15	1.500	12	1.091	28	266	5.115	12	626.65	6.084	19	164	3.727	22	1.048	14	25	1.190
1	38.5	0.811				17	24	0.490	23	0.548	2	34	0.680	10	17	0.567					13	28	0.538				8	26.5	0.602	10	0.476	3	14	0.667
									23	0.548				11	17.5	0.583	2	0.200			13	28	0.538				9	27	0.614					
									24	0.571				12	18.5	0.617					15	33	0.635	1	58	0.563	10	29	0.659					
2	39.5	0.832				17	24	0.490	26	0.619	5	40	0.800								16	37	0.712				11	31	0.705	11	0.524	4	15	0.714
4	40.5	0.853	1	77	0.786	25	30	0.612	34	0.810				13	20	0.667	8	0.800			19	47.5	0.913	2	69.5	0.675	12	32	0.727	16	0.762	6	17	0.810
						12	21.66	0.442	4	0.095	1	30	0.600	8	14.5	0.483	2	0.200	2	0.182	10	22	0.423				6	24	0.545	4	0.190	1	12	0.571
1	38.5	0.811				17	24	0.490	32	0.762				10	17	0.567			2	0.182	13	28	0.538	1	58	0.563	9	27	0.614					
3	40	0.842				28	33	0.673	33	0.786	6	42.5	0.850	11	17.5	0.583	5	0.500	5	0.455	18	44	0.846				13	34	0.773					
			1	77	0.786				37	0.881				14	22	0.733					22	54	1.038	3	81	0.786	14	36	0.818	11	0.524	4	15	0.714
4	40.5	0.853				36	40	0.816	39	0.929	3	37	0.740	15	24	0.800	8	0.800	7	0.636	23	56.5	1.087	5	97	0.942				19	0.905	6	17	0.810
8	47.5	1.000	2	98	1.000	39	49	1.000	42	1.000	8	50	1.000	17	30	1.000	10	1.000	11	1.000	21	52	1.000	7	103	1.000	16	44	1.000	21	1.000	10	21	1.000

1	38.5	0.811				14	21.88	0.447	11	0.262	1	30	0.600	9	16	0.533	2	0.200	2	0.182	12	26	0.500				7	26	0.591	5	0.238	2	13	0.619
1	38.5	0.811				17	24	0.490	21	0.500	1	30	0.600	10	17	0.567	3	0.300	3	0.273	12	26	0.500				8	26.5	0.602	8	0.381			
						23	28.5	0.582			2	34	0.680	11	17.5	0.583					13	28	0.538				9	27	0.614	11	0.524	4	15	0.714
1	38.5	0.811				14	21.88	0.447	21	0.500				9	16	0.533	2	0.200	2	0.182	12	26	0.500				7	26	0.591	5	0.238			
1	38.5	0.811				15	22	0.449	21	0.500				10	17	0.567	2	0.200	2	0.182	12	26	0.500				7	26	0.591	8	0.381			
4	40.5	0.853	2	98	1.000	28	33	0.673	36	0.857	7	45	0.900	14	22	0.733	8	0.800	7	0.636	18	44	0.846	3	81	0.786	13	34	0.773	17	0.810	6	17	0.810
1	38.5	0.811	1	77	0.786	12	21.66	0.442	18	0.429	2	34	0.680	9	16	0.533	2	0.200	2	0.182	12	26	0.500	1	58	0.563	8	26.5	0.602	10	0.476	4	15	0.714
6	43.16	0.909	4	120	1.224	38	42.5	0.867	42	1.000	7	45	0.900	17	30	1.000	8	0.800	11	1.000	22	54	1.038	7	103	1.000	16	44	1.000	20	0.952	8	19	0.905
1	38.5	0.811	1	77	0.786	17	24	0.490	28	0.667	2	34	0.680	12	18.5	0.617	11	1.100	2	0.182	13	28	0.538				9	27	0.614			3	14	0.667
4	40.5	0.853	2	98	1.000	31	35.33	0.721	40	0.952	6	42.5	0.850	13	20	0.667					19	47.5	0.913	4	92	0.893	15	36.5	0.830	19	0.905	6	17	0.810
8	47.5	1.000	4	120	1.224	40	55	1.122	42	1.000	8	50	1.000	17	30	1.000	11	1.100	10	0.909	24	70	1.346	6	100	0.971	17	53	1.205	21	1.000	10	21	1.000
			2	98	1.000	33	36.67	0.748						14	22	0.733	9	0.900			20	50.5	0.971	5	97	0.942								
			3	112	1.143	36	40	0.816	41	0.976				16	26.5	0.883					21	52	1.000	6	100	0.971				20	0.952			
11	72.5	1.526	10	660	6.735	48	335	6.837	44	1.048	9	170	3.400	23	81.5	2.717	13	1.300			28	205	3.942	12	626.65	6.084	19	137.5	3.125	23	1.095	15	43	2.048
1	38.5	0.811							41	0.976				18	35	1.167																8	19	0.905
									40	0.952				17	30	1.000	11	1.100												20	0.952	6	17	0.810
1	38.5	0.811				10	21.44	0.438	26	0.619	1	30	0.600	9	16	0.533	11	1.100	2	0.182	11	24	0.462	1	58	0.563	7	26	0.591	9	0.429	3	14	0.667
4	40.5	0.853	1	77	0.786	13	21.77	0.444	39	0.929	2	34	0.680	13	20	0.667	14	1.400	5	0.455	13	28	0.538	3	81	0.786	9	27	0.614	10	0.476	5	16	0.762

IW1275			IW1313		IW1305		IW1314		IW1272			IW1340			IW1446			IW1315			IW1451			IW1342		IW1332			IW1308			IW1345		
Mesocricetus auratus			*Miniopterus natalensis*		*Miniopterus schreibersii*		*Molossus rufus*		*Mus musculus*			*Mustela putorius*			*Myodes glareolus*			*Myotis lucifugus*			*Myotis myotis*			*Nycticebus cougang*		*Oryctolagus cuniculus*			*Ovis aries*			*Papio cynocephalus*		
Rank	Abs. time (d)	Scaled	Rank	Scaled	Rank	Scaled	Rank	Scaled	Rank	Abs. time (d)	Scaled	Rank	Abs. time (d)	Scaled	Rank	Abs. time (d)	Scaled	Rank	Abs. time (d)	Scaled	Rank	Abs. time (d)	Scaled	Rank	Scaled	Rank	Abs. time (d)	Scaled	Rank	Abs. time (d)	Scaled	Rank	Abs. time (d)	Scaled
2	7	0.538			1	0.111			9	5.5	0.344	1	12	0.429												2	6.5	0.382	1	13.5	0.397	1	17	0.362
5	8	0.615							12	8.125	0.508	3	16	0.571										2	0.250	7	8.4	0.494	3	15.5	0.456	3	25	0.532
7	8.5	0.654			2	0.222			14	9.125	0.570	4	17	0.607	1	9.5	0.655							2	0.250	10	9.5	0.559	5	17	0.500	4	27	0.574
9	9	0.692			3	0.333			17	10.625	0.664	4	17	0.607	1	9.5	0.655							2	0.250	10	9.5	0.559	5	17	0.500	5	28	0.596
5	8	0.615							12	8.125	0.508	2	15	0.536												7	8.4	0.494	3	15.5	0.456			
3	7.5	0.577							11	7.625	0.477	3	16	0.571										2	0.250	8	8.6	0.506	2	14.5	0.426	2	23	0.489
7	8.5	0.654							12	8.125	0.508	4	17	0.607										2	0.250	10	9.5	0.559	3	15.5	0.456	2	23	0.489
19	13	1.000	16	1.333					24	16	1.000	17	30	1.071	5	18.25	1.259				3	39	0.780	9	1.125	25	29	1.706	26	40	1.176	18	45	0.957
9	9	0.692					2	0.250	18	10.875	0.680	5	18	0.643	1	9.5	0.655				1	27	0.540			11	10	0.588				6	29	0.617
10	9.25	0.712	5	0.417	5	0.556	2	0.250	19	11.625	0.727	6	19	0.679				2	32	0.744	2	32	0.640			12	10.5	0.618	25	35	1.029	8	30.5	0.649
15	11	0.846	9	0.750	7	0.778	4	0.500	20	12.25	0.766	11	24	0.857	2	11.75	0.810	3	39	0.907	3	39	0.780	8	1.000	20	16	0.941	21	30	0.882	13	36.5	0.777
6	8.25	0.635							14	9.125	0.570	4	17	0.607	1	9.5	0.655									9	9	0.529	5	17	0.500	3	25	0.532
8	8.75	0.673	1	0.083	2	0.222	1	0.125	15	9.125	0.570	5	18	0.643	2	11.75	0.810				1	27	0.540	1	0.125	10	9.5	0.559	14	23.75	0.699	4	27	0.574
14	10.5	0.808	9	0.750			3	0.375	20	12.25	0.766	8	21	0.750	3	14.5	1.000				3	39	0.780	8	1.000				18	25	0.735	12	35.5	0.755
15	11	0.846	11	0.917	7	0.778	4	0.500	21	13.25	0.828	10	23	0.821	3	14.5	1.000	2	32	0.744	3	39	0.780	7	0.875	17	13	0.765	19	26	0.765	11	34.5	0.734
10	9.25	0.712	1	0.083	2	0.222			12	8.125	0.508	3	16	0.571										1	0.125	8	8.6	0.506	8	19	0.559	5	28	0.596
10	9.25	0.712	5	0.417	4	0.444	1	0.125	16	10.125	0.633	6	19	0.679	1	9.5	0.655	1	27	0.628	1	27	0.540	5	0.625	11	10	0.588	17	24.75	0.728	7	30	0.638
5	8	0.615	5	0.417			1	0.125				11	24	0.857	1	9.5	0.655	1	27	0.628	2	32	0.640	7	0.875				16	24.25	0.713	6	29	0.617
			9	0.750	6	0.667	3	0.375	16	10.125	0.633	10	23	0.821	2	11.75	0.810	2	32	0.744	2	32	0.640	6	0.750	16	12.5	0.735	19	26	0.765	9	31.5	0.670
			6	0.500	5	0.556	4	0.500				8	21	0.750							2	32	0.640	7	0.875	19	15	0.882	25	35	1.029	11	34.5	0.734
6	8.25	0.635	1	0.083					12	8.125	0.508	3	16	0.571										2	0.250	10	9.5	0.559	5	17	0.500	3	25	0.532
			11	0.917	8	0.889	3	0.375	21	13.25	0.828	14	27	0.964	4	15.5	1.069	3	39	0.907	4	43	0.860			18	14	0.824	15	24	0.706	12	35.5	0.755
7	8.5	0.654			5	0.556			14	9.125	0.570	5	18	0.643										2	0.250	11	10	0.588	7	18	0.529	5	28	0.596
10	9.25	0.712	1	0.083	3	0.333			16	10.125	0.633	5	18	0.643	1	9.5	0.655							5	0.625	11	10	0.588	6	17.25	0.507	4	27	0.574
8	8.75	0.673	1	0.083					14	9.125	0.570	5	18	0.643												11	10	0.588	9	19.75	0.581	5	28	0.596
9	9	0.692	1	0.083			1	0.125	16	10.125	0.633	5	18	0.643	1	9.5	0.655				1	27	0.540			12	10.5	0.618				6	29	0.617
9	9	0.692	3	0.250	3	0.333	1	0.125	15	9.125	0.570				1	9.5	0.655				2	32	0.640	5	0.625	12	10.5	0.618	11	20.75	0.610	8	30.5	0.649
10	9.25	0.712	5	0.417	4	0.444	2	0.250	17	10.625	0.664	7	20	0.714										5	0.625	13	11	0.647	11	20.75	0.610	9	31.5	0.670
10	9.25	0.712	4	0.333	5	0.556	2	0.250	16	10.125	0.633							1	27	0.628				6	0.750	16	12.5	0.735	12	21.25	0.625	9	31.5	0.670
11	9.5	0.731	6	0.500					18	10.875	0.680	8	21	0.750	2	11.75	0.810	1	27	0.628	2	32	0.640			16	12.5	0.735	15	24	0.706	10	33	0.702
12	9.75	0.750	2	0.167	4	0.444	1	0.125	17	10.625	0.664	7	20	0.714															16	24.25	0.713	11	34.5	0.734
18	12.5	0.962	5	0.417	8	0.889	2	0.250	23	15	0.938	13	26	0.929	3	14.5	1.000	4	43	1.000	3	39	0.780	7	0.875	18	14	0.824	17	24.75	0.728	14	37.5	0.798
19	13	1.000	9	0.750	8	0.889	5	0.625	25	17	1.063	13	26	0.929	5	18.25	1.259	3	39	0.907	4	43	0.860	9	1.125	23	18	1.059	18	25	0.735	16	41	0.872
10	9.5	0.731	6	0.500					19	11.625	0.727	6	19	0.679	2	11.75	0.810	1	27	0.628	2	32	0.640	6	0.750	17	13	0.765	15	24	0.706	10	33	0.702
12	9.75	0.750	7	0.583					19	11.625	0.727	9	22	0.786				2	32	0.744				7	0.875	17	13	0.765	15	24	0.706	12	35.5	0.755
12	9.75	0.750	7	0.583			3	0.375	21	13.25	0.828	7	20	0.714				2	32	0.744	3	39	0.780	6	0.750	18	14	0.824	16	24.25	0.713	11	34.5	0.734
14	10.5	0.808	8	0.667	6	0.667	3	0.375	21	13.25	0.828	10	23	0.821										7	0.875	19	15	0.882	16	24.25	0.713	12	35.5	0.755
			8	0.667	6	0.667	3	0.375	21	13.25	0.828	11	24	0.857	3	14.5	1.000	3	39	0.907	3	39	0.780	7	0.875	19	15	0.882	20	28	0.824	13	36.5	0.777
15	11	0.846	10	0.833			4	0.500	21	13.25	0.828	11	24	0.857	3	14.5	1.000	3	39	0.907	3	39	0.780			19	15	0.882	21	30	0.882	15	39	0.830
16	11.5	0.885	8	0.667			3	0.375	21	13.25	0.828	10	23	0.821	3	14.5	1.000	3	39	0.907	3	39	0.780			19	15	0.882	22	31	0.912	13	36.5	0.777
15	11	0.846	10	0.833	8	0.889	4	0.500	21	13.25	0.828	11	24	0.857	3	14.5	1.000	3	39	0.907	3	39	0.780			19	15	0.882	22	31	0.912	16	41	0.872
18	12.5	0.962	12	1.000	9	1.000	7	0.875	23	15	0.938	15	28	1.000	4	15.5	1.069	4	43	1.000	3	39	0.780	8	1.000	21	16.5	0.971	23	32	0.941	18	45	0.957
18	12.5	0.962	13	1.083	9	1.000	6	0.750	23	15	0.938	16	29	1.036	4	15.5	1.069	4	43	1.000	4	43	0.860	8	1.000	22	17	1.000	25	35	1.029	19	47	1.000
19	13	1.000	14	1.167	10	1.111	7	0.875	27	19.5	1.219	19	32	1.143	5	18.25	1.259	4	43	1.000	6	50	1.000	8	1.000	24	20	1.176	34	70.5	2.074	20	190	4.043
19	13	1.000	14	1.167	10	1.111	8	1.000	27	19.5	1.219	20	35	1.250	5	18.25	1.259	4	43	1.000	6	50	1.000	8	1.000	23	18	1.059	34	70.5	2.074	20	180	3.830
21	16	1.231	17	1.417	16	1.778	11	1.375	27	19.5	1.219	24	42	1.500	8	20	1.379	8	60	1.395	9	70	1.400	10	1.250	25	29	1.706	41	151	4.441	20	180	3.830
8	8.75	0.673	5	0.417	4	0.444	12	1.500	17	10.625	0.664	5	18	0.643	1	9.5	0.655				1	27	0.540	5	0.625	10	9.5	0.559	7	18	0.529	6	29	0.617
															1	9.5	0.655				1	27	0.540	5	0.625				9	19.75	0.581	6	29	0.617
9	9	0.692			4	0.444			18	10.875	0.680	6	19	0.679												13	11	0.647	10	20.25	0.596	8	30.5	0.649
10	9.25	0.712	5	0.417	5	0.556						7	20	0.714	2	11.75	0.810	1	27	0.628	2	32	0.640	6	0.750	16	12.5	0.735	17	24.75	0.728	10	33	0.702
15	11	0.846	9	0.750	6	0.667	4	0.500	22	14.25	0.891	11	24	0.857	3	14.5	1.000	3	39	0.907	3	39	0.780	7	0.875	19	15	0.882	19	26	0.765	15	39	0.830
5	8	0.615					1	0.125	12	8.125	0.508	4	17	0.607	1	9.5	0.655				1	27	0.540	2	0.250	10	9.5	0.559	6	17.25	0.507	3	25	0.532
									13	8.625	0.539															13	11	0.647	13	22	0.647	5	28	0.596
8	8.75	0.673	5	0.417	5	0.556	1	0.125	19	11.625	0.727	10	23	0.821				1	27	0.628	1	27	0.540			14	11.5	0.676	17	24.75	0.728	7	30	0.638
9	9	0.692					2	0.250	21	13.25	0.828				1	9.5	0.655				2	32	0.640	6	0.750	19	15	0.882	19	26	0.765	13	36.5	0.777
10	9.25	0.712	9	0.750	6	0.667	5	0.625	22	14.25	0.891	14	27	0.964	2	11.75	0.810	2	32	0.744	3	39	0.780	7	0.875	20	16	0.941	21	30	0.882	15	39	0.830
19	13	1.000	12	1.000	9	1.000	8	1.000	24	16	1.000	15	28	1.000	3	14.5	1.000	4	43	1.000	6	50	1.000	8	1.000	22	17	1.000	24	34	1.000	19	47	1.000

7	8.5	0.654	1	0.083	2	0.222	1	0.125	13	8.625	0.539	4	17	0.607	1	9.5	0.655				1	27	0.540	3	0.375	10	9.5	0.559	7	18	0.529	3	25	0.532
8	8.75	0.673	1	0.083	3	0.333	1	0.125	14	9.125	0.570	4	17	0.607	2	11.75	0.810				1	27	0.540	4	0.500	11	10	0.588	8	19	0.559	5	28	0.596
9	9	0.692							16	10.125	0.633	5	18	0.643							1	27	0.540			12	10.5	0.618	8	19	0.559	6	29	0.617
5	8	0.615	1	0.083			1	0.125	14	9.125	0.570				1	9.5	0.655				1	27	0.540	2	0.250	10	9.5	0.559	6	17.25	0.507	5	28	0.596
8	8.75	0.673	1	0.083			1	0.125	18	10.875	0.680				1	9.5	0.655				1	27	0.540	2	0.250	11	10	0.588	6	17.25	0.507	5	28	0.596
15	11	0.846	9	0.750	7	0.778	3	0.375	21	13.25	0.828	10	23	0.821	3	14.5	1.000	2	32	0.744	3	39	0.780	7	0.875	17	13	0.765	16	24.25	0.713	15	39	0.830
7	8.5	0.654	1	0.083	4	0.444	1	0.125	14	9.125	0.570	6	19	0.679	1	9.5	0.655	1	27	0.628	1	27	0.540	4	0.500	16	12.5	0.735	8	19	0.559	6	29	0.617
17	12	0.923	12	1.000	9	1.000	5	0.625	23	15	0.938	14	27	0.964	3	14.5	1.000	4	43	1.000	3	39	0.780	9	1.125	22	17	1.000	26	40	1.176	18	45	0.957
10	9.25	0.712	1	0.083	1	0.111	1	0.125	13	8.625	0.539	5	18	0.643	2	11.75	0.810	1	27	0.628	1	27	0.540	5	0.625	11	10	0.588	7	18	0.529	6	29	0.617
15	11	0.846	11	0.917	7	0.778	5	0.625	24	16	1.000	12	25	0.893	3	14.5	1.000				3	39	0.780	8	1.000	19	15	0.882	18	25	0.735	13	36.5	0.777
			15	1.250	11	1.222	8	1.000				17	30	1.071				7	57	1.326	5	45	0.900	9	1.125	24	20	1.176	28	43	1.265	18	45	0.957
16	11.5	0.885	13	1.083	8	0.889			25	17	1.063	13	26	0.929	5	18.25	1.259				4	43	0.860	8	1.000	21	16.5	0.971	18	25	0.735	15	39	0.830
18	12.5	0.962	14	1.167			6	0.750				13	26	0.929				6	50	1.163				8	1.000	22	17	1.000	19	26	0.765	17	43	0.915
22	28	2.154							29	32	2.000	25	76	2.714	9	29	2.000	9	62.29	1.449	10	77	1.500	10	1.250	26	37.5	2.206	39	119	3.500	20	180	3.830
17	12	0.923	12	1.000			4	0.500	23	15	0.938				4	15.5	1.069				6	50	1.000			19	15	0.882						
15	11	0.846	12	1.000	8	0.889	4	0.500	21	13.25	0.828	9	22	0.786	3	14.5	1.000				3	39	0.780	8	1.000	19	15	0.882	24	34	1.000			
9	9	0.692			4	0.444			13	8.625	0.539	4	17	0.607												10	9.5	0.559	7	18	0.529	5	28	0.596
11	9.5	0.731			5	0.556	3	0.375	14	9.125	0.570	5	18	0.643	3	14.5	1.000				1	27	0.540			16	12.5	0.735	10	20.25	0.596	8	30.5	0.649

IW1319 *Phocoena phocoena*		IW1320 *Pipistrellus abramus*		IW1300 *Rangifer tarandus*			IW1350 *Rattus norvegicus*			IW1321 *Scotophilus kuhlii*		IW1279 *Spermophilus citellus*		IW1283 *Stenella attenuata*			IW1284 *Stenella longirostris*			IW1288 *Sus scrofa domesticus*			IW1354 *Talpa europaea*			IW1355 *Tarsius tarsier*		IW1417 *Tupaia belangeri*			IW1281 *Tupaia javanica*	
Rank	Scaled	Rank	Scaled	Rank	Abs. time (d)	Scaled	Rank	Abs. time (d)	Scaled	Rank	Scaled	Rank	Scaled	Rank	Abs. time (d)	Scaled	Rank	Abs. time (d)	Scaled	Rank	Abs. time (d)	Scaled	Rank	Abs. time (d)	Scaled	Rank	Scaled	Rank	Abs. time (d)	Scaled	Rank	Scaled
							1	8.5	0.472	1	0.045	4	0.089							1	9	0.286	1	1.86	0.093			1	9	0.310		
		1	0.100				5	9.9	0.550	2	0.091	11	0.244	1	12.75	0.280	1	12.75	0.280	4	14	0.444	2	3.71	0.186	2	0.105	2	12	0.414	4	0.167
1	0.200	3	0.300	1	31.5	0.623	10	10.58	0.588	7	0.318	21	0.467															3	14	0.483	7	0.292
1	0.200	5	0.500	1	31.5	0.623	12	11	0.611	6	0.273	21	0.467	3	13.75	0.302	3	13.75	0.302	6	16.625	0.528				2	0.105	3	14	0.483	7	0.292
		1	0.100				4	9.55	0.531	3	0.136	10	0.222	2	13.25	0.291	2	13.25	0.291							1	0.053	2	12	0.414	1	0.042
							2	8.85	0.492	3	0.136	9	0.200	2	13.25	0.291	2	13.25	0.291	4	14	0.444	3	5.57	0.279	1	0.053	1	9	0.310	1	0.042
		1	0.100				7	10.32	0.573	5	0.227	11	0.244	3	13.75	0.302	3	13.75	0.302	5	16.5	0.524				2	0.105	2	12	0.414	4	0.167
6	1.200	11	1.100				27	19	1.056	24	1.091	45	1.000	13	70	1.538	13	70	1.538	33	49	1.556	10	20	1.000	17	0.895	6	29	1.000	24	1.000
							15	11.72	0.651	7	0.318	22	0.489	3	13.75	0.302	3	13.75	0.302	11	17.25	0.548				2	0.105	4	18	0.621	7	0.292
3	0.600	6	0.600	3	39.2	0.775	16	11.79	0.655	11	0.500	25	0.556	6	23.5	0.516	6	23.5	0.516	14	19.5	0.619				5	0.263	5	24	0.828	14	0.583
3	0.600	9	0.900	5	46.8	0.925	22	14.58	0.810	17	0.773	34	0.756	8	30	0.659	8	30	0.659	26	24.75	0.786				12	0.632	5	24	0.828	19	0.792
		3	0.300				7	10.32	0.573	5	0.227	13	0.289	3	13.75	0.302	3	13.75	0.302												2	0.083
1	0.200	5	0.500	1	31.5	0.623	9	10.48	0.582	6	0.273	15	0.333	4	14	0.308	4	14	0.308	5	16.5	0.524	5	9.29	0.465	4	0.211	3	14	0.483	3	0.125
3	0.600	10	1.000				16	11.79	0.655	13	0.591	27	0.600										9	18	0.900	9	0.474	5	24	0.828	13	0.542
4	0.800	9	0.900	4	43	0.850	20	13.5	0.750	15	0.682	39	0.867	8	30	0.659	8	30	0.659	27	27.25	0.865	9	18	0.900	11	0.579	5	24	0.828	18	0.750
		4	0.400	3	39.2	0.775	11	10.75	0.597	5	0.227	13	0.289	3	13.75	0.302	3	13.75	0.302	9	17	0.540	4	7.45	0.373	3	0.158	3	14	0.483	6	0.250
2	0.400	6	0.600	3	39.2	0.775	17	12.25	0.681	11	0.500	25	0.556	6	23.5	0.516	6	23.5	0.516	12	17.5	0.556				4	0.211	3	14	0.483	8	0.333
		8	0.800				18	12.5	0.694	12	0.545	26	0.578	8	30	0.659	8	30	0.659	14	19.5	0.619				4	0.211	3	14	0.483	10	0.417
4	0.800	8	0.800				19	13	0.722	14	0.636	27	0.600	7	24.5	0.538	7	24.5	0.538	17	21	0.667				7	0.368	4	18	0.621	11	0.458
5	1.000	7	0.700	4	43	0.850	21	14	0.778	12	0.545	35	0.778	8	30	0.659	8	30	0.659	17	21	0.667	8	15	0.750	10	0.526	4	18	0.621	13	0.542
1	0.200	1	0.100	1	31.5	0.623	8	10.39	0.577	3	0.136	12	0.267	3	13.75	0.302	3	13.75	0.302	7	16.75	0.532				2	0.105	3	14	0.483	4	0.167
5	1.000	9	0.900				21	14	0.778	21	0.955	42	0.933	8	30	0.659	8	30	0.659	28	29	0.921				14	0.737	5	24	0.828	20	0.833
1	0.200	2	0.200	1	31.5	0.623	9	10.46	0.581	5	0.227	14	0.311	5	14.25	0.313	5	14.25	0.313	8	16.875	0.536	6	11.14	0.557	2	0.105	3	14	0.483	8	0.333
1	0.200	3	0.300				11	10.75	0.597	7	0.318			4	14	0.308	4	14	0.308	10	17.125	0.544	7	13	0.650	3	0.158				8	0.333
1	0.200	4	0.400	1	31.5	0.623	11	10.75	0.597	10	0.455	19	0.422	3	13.75	0.302	3	13.75	0.302	11	17.25	0.548				4	0.211	3	14	0.483	8	0.333
1	0.200	5	0.500	1	31.5	0.623	17	12.25	0.681	10	0.455	24	0.533							13	17.75	0.563				5	0.263				11	0.458
2	0.400	5	0.500	2	35.3	0.698	17	12.25	0.681	11	0.500	25	0.556	6	23.5	0.516	6	23.5	0.516	12	17.5	0.556	7	13	0.650	6	0.316				12	0.500
3	0.600	7	0.700	2	35.3	0.698	18	12.5	0.694	12	0.545	30	0.667	6	23.5	0.516	6	23.5	0.516	14	19.5	0.619	7	13	0.650						13	0.542
3	0.600	6	0.600				19	13	0.722	12	0.545	31	0.689	7	24.5	0.538	7	24.5	0.538	14	19.5	0.619										
3	0.600	7	0.700	3	39.2	0.775	20	13.5	0.750	13	0.591	33	0.733	8	30	0.659	8	30	0.659	17	21	0.667						4	18	0.621	14	0.583
		6	0.600				18	12.5	0.694			28	0.622	8	30	0.659	8	30	0.659	17	21	0.667									14	0.583
5	1.000	10	1.000	4	43	0.850	18	12.5	0.694	12	0.545	32	0.711							20	22.5	0.714	9	18	0.900	12	0.632	6	29	1.000	16	0.667
		10	1.000	4	43	0.850	22	14.58	0.810	17	0.773	38	0.844							25	24.75	0.786	10	20	1.000	11	0.579	4	18	0.621	18	0.750
3	0.600	7	0.700	3	39.2	0.775	20	13.5	0.750	12	0.545	31	0.689	8	30	0.659	8	30	0.659	21	23	0.730				7	0.368				14	0.583
3	0.600						21	14	0.778			34	0.756							21	23	0.730				8	0.421	4	18	0.621	15	0.625
4	0.800	8	0.800	4	43	0.850	21	14	0.778	13	0.591	32	0.711							22	23.33	0.741	8	15	0.750	10	0.526	4	18	0.621	15	0.625
		8	0.800	4	43	0.850	22	14.58	0.810	14	0.636	35	0.778							24	24	0.762				11	0.579				16	0.667
4	0.800	8	0.800				20	13.5	0.750	16	0.727	36	0.800	9	38	0.835	9	38	0.835	25	24.75	0.786				12	0.632	5	24	0.828	18	0.750
							22	14.58	0.810	18	0.818	39	0.867							26	25.5	0.810				12	0.632	5	24	0.828	18	0.750
5	1.000	9	0.900				21	14	0.778	14	0.636	38	0.844	10	45.5	1.000	10	45.5	1.000	28	29	0.921	9	18	0.900	11	0.579	5	24	0.828	19	0.792
							23	15	0.833	21	0.955	39	0.867							28	29	0.921	9	18	0.900	12	0.632	5	24	0.828	19	0.792
		10	1.000	5	46.8	0.925	26	16.51	0.917	22	1.000	42	0.933							30	32.5	1.032	10	20	1.000	15	0.789	6	29	1.000	20	0.833
		10	1.000	5	46.8	0.925	27	17	0.944	22	1.000	44	0.978							30	32.5	1.032	10	20	1.000	16	0.842	6	29	1.000	22	0.917
		11	1.100				28	17.5	0.972	22	1.000	47	1.044							32	40	1.270	11	22	1.100	17	0.895	7	44	1.517	22	0.917
		11	1.100	7	54.5	1.077	28	17.5	0.972	23	1.045	46	1.022							32	40	1.270	11	22	1.100	18	0.947	7	44	1.517	24	1.000
8	1.600	14	1.400	8	280	5.534	32	22	1.222	24	1.091	50	1.111	18	280	6.154	18	280	6.154	36	114	3.619	14	29	1.450	22	1.158	7	44	1.517	25	1.042
1	0.200	5	0.500	1	31.5	0.623	13	11.6	0.644	9	0.409	22	0.489	3	13.75	0.302	3	13.75	0.302	11	17.25	0.548	7	13	0.650	5	0.263	3	14	0.483	7	0.292
1	0.200	5	0.500				16	11.79	0.655	10	0.455	23	0.511	3	13.75	0.302	3	13.75	0.302	11	17.25	0.548				7	0.368	3	14	0.483	8	0.333
		6	0.600							11	0.500	25	0.556	4	14	0.308	4	14	0.308	13	17.75	0.563				8	0.421				9	0.375
2	0.400	7	0.700	3	39.2	0.775	17	12.25	0.681	12	0.545	32	0.711	6	23.5	0.516	6	23.5	0.516	16	20.5	0.651				10	0.526	4	18	0.621	13	0.542
4	0.800	9	0.900	4	43	0.850	21	14	0.778	17	0.773	37	0.822	8	30	0.659	8	30	0.659	24	24	0.762				12	0.632	5	24	0.828	17	0.708
1	0.200	1	0.100				5	9.9	0.550	5	0.227	13	0.289	3	13.75	0.302	3	13.75	0.302	5	16.5	0.524	4	7.43	0.372	2	0.105	3	14	0.483	2	0.083
1	0.200			1	31.5	0.623	10	10.58	0.588	11	0.500	27	0.600	3	13.75	0.302	3	13.75	0.302							6	0.316				8	0.333
				3	39.2	0.775	11	10.75	0.597	12	0.545	38	0.844	6	23.5	0.516	6	23.5	0.516	11	17.25	0.548				8	0.421	3	14	0.483	10	0.417
		5	0.500				15	11.72	0.651	14	0.636	40	0.889	8	30	0.659	8	30	0.659	22	23.33	0.741				11	0.579				14	0.583
2	0.400	7	0.700				23	15	0.833	14	0.636	42	0.933	9	38	0.835	9	38	0.835	24	24	0.762	9	18	0.900	19	1.000	5	24	0.828	18	0.750
5	1.000	10	1.000	6	50.6	1.000	27	18	1.000	22	1.000	45	1.000	10	45.5	1.000	10	45.5	1.000	29	31.5	1.000	10	20	1.000	19	1.000	6	29	1.000	24	1.000

1	0.200	2	0.200	1	31.5	0.623	7	10.32	0.573	6	0.273	13	0.289	3	13.75	0.302	3	13.75	0.302	7	16.75	0.532	4	7.44	0.372	2	0.105	3	14	0.483	6	0.250
1	0.200	4	0.400	1	31.5	0.623	13	11.6	0.644	7	0.318	20	0.444	3	13.75	0.302	3	13.75	0.302	10	17.125	0.544	7	13	0.650	3	0.158	3	14	0.483	6	0.250
2	0.400			1	31.5	0.623	16	11.79	0.655			23	0.511	4	14	0.308	4	14	0.308	15	20	0.635	7	13	0.650						12	0.500
1	0.200	3	0.300	1	31.5	0.623	9	10.47	0.582	7	0.318	9	0.200	3	13.75	0.302	3	13.75	0.302	7	16.75	0.532	7	13	0.650	3	0.158	3	14	0.483	6	0.250
1	0.200	3	0.300	1	31.5	0.623	11	10.75	0.597	8	0.364	13	0.289	3	13.75	0.302	3	13.75	0.302	10	17.125	0.544	7	13	0.650	3	0.158	3	14	0.483	6	0.250
4	0.800	8	0.800				20	13.5	0.750	14	0.636	36	0.800	5	14.25	0.313	5	14.25	0.313				9	18	0.900	11	0.579	5	24	0.828	18	0.750
1	0.200	5	0.500	1	31.5	0.623	18	12.5	0.694	10	0.455	13	0.289	3	13.75	0.302	3	13.75	0.302	10	17.125	0.544				4	0.211	3	14	0.483	6	0.250
5	1.000	10	1.000				26	16.51	0.917	22	1.000	43	0.956	9	38	0.835	9	38	0.835	28	29	0.921	10	20	1.000	17	0.895	5	24	0.828	20	0.833
2	0.400	5	0.500	1	31.5	0.623	15	11.72	0.651	10	0.455			6	23.5	0.516	6	23.5	0.516	14	19.5	0.619				4	0.211	3	14	0.483	7	0.292
4	0.800	10	1.000	4	43	0.850	23	15	0.833	17	0.773	40	0.889	8	30	0.659	8	30	0.659	26	25.5	0.810				12	0.632	5	24	0.828	17	0.708
6	1.200	11	1.100				28	17.5	0.972	22	1.000	46	1.022	12	57	1.253	12	57	1.253	32	40	1.270	11	22	1.100	19	1.000	6	29	1.000	25	1.042
5	1.000	10	1.000	6	50.6	1.000				18	0.818	42	0.933	9	38	0.835	9	38	0.835							17	0.895				20	0.833
				7	54.5	1.077				21	0.955	45	1.000	11	46.5	1.022	11	46.5	1.022	28	29	0.921				18	0.947				24	1.000
8	1.600	15	1.500	8	280	5.534	33	35	1.944			51	1.133	15	100	2.198	15	100	2.198	36	114	3.619	15	51	2.550	22	1.158	9	61.5	2.121	26	1.083
		11	1.100				22	14.58	0.810	17	0.773	39	0.867							28	27.25	0.865	10	20	1.000	15	0.789	6	29	1.000	20	0.833
		10	1.000				21	14	0.778	18	0.818	39	0.867							28	27.25	#BEZU	9	18	0.900	15	0.789	5	24	0.828	20	0.833
1	0.200	1	0.100				7	10.32	0.573	7	0.318	15	0.333	1	12.75	0.280	1	12.75	0.280	7	16.75	0.532				2	0.105	3	14	0.483	7	0.292
2	0.400	7	0.700				19	13	0.722	11	0.500	31	0.689	5	14.25	0.313	5	14.25	0.313	15	20	0.635				4	0.211	4	18	0.621	16	0.667

Data Sources

Sources of all developmental data for placental mammals extracted by Werneburg et al. (2016). Sources for the final 51 species chosen for analyses can be related to data points above by matching their internal tracking IDs (IW #).

#	Species	Calculated absolute time	Referred absolute time (see reference)	Specimen/figure/stage, as used in the studies	Reference
IW1444	*Acomys dimidiatus*		days-hours		
IW1444-01	*Acomys dimidiatus*	13.875	13-21	description following age, Fig. 2-6	Dieterlen (1963)
IW1444-02	*Acomys dimidiatus*	15.21	15-05	description following age, Fig. 2-6	Dieterlen (1963)
IW1444-03	*Acomys dimidiatus*	17.2	17-12	description following age, Fig. 2-6	Dieterlen (1963)
IW1444-04	*Acomys dimidiatus*	18.75	18-18	description following age, Fig. 2-6	Dieterlen (1963)
IW1444-05	*Acomys dimidiatus*	19.54	19-13	description following age, Fig. 2-6	Dieterlen (1963)
IW1444-06	*Acomys dimidiatus*	21.71	21-17	description following age, Fig. 2-6	Dieterlen (1963)
IW1444-07	*Acomys dimidiatus*	22.67	22-16	description following age, Fig. 2-6	Dieterlen (1963)
IW1444-08	*Acomys dimidiatus*	23.625	23-15	description following age, Fig. 2-6	Dieterlen (1963)
IW1444-09	*Acomys dimidiatus*	24.9	25-11	description following age, Fig. 2-6	Dieterlen (1963)
IW1444-10	*Acomys dimidiatus*	26.18	26-11	description following age, Fig. 2-6	Dieterlen (1963)
IW1444-11	*Acomys dimidiatus*	27.46	27-11	description following age, Fig. 2-6	Dieterlen (1963)
IW1444-12	*Acomys dimidiatus*	28.375	28-09	description following age, Fig. 2-6	Dieterlen (1963)
IW1444-13	*Acomys dimidiatus*	29.375	29-09	description following age, Fig. 2-6	Dieterlen (1963)
IW1444-14	*Acomys dimidiatus*	31.432	31-10	description following age, Fig. 2-6	Dieterlen (1963)
IW1444-15	*Acomys dimidiatus*	32.54	32-13	description following age, Fig. 2-6	Dieterlen (1963)
IW1444-16	*Acomys dimidiatus*	33.412	33-10	description following age, Fig. 2-6	Dieterlen (1963)
IW1444-17	*Acomys dimidiatus*	35.67	35-16	description following age, Fig. 2-6	Dieterlen (1963)
IW1444-18	*Acomys dimidiatus*	36	36	description following age, Fig. 2-6	Dieterlen (1963)
IW1444-19	*Acomys dimidiatus*	37.5	37-38	description following age, Fig. 2-6, birth, eye open	Dieterlen (1963)
IW1444-20	*Acomys dimidiatus*	38.5	38-39	description following age, Fig. 2-6	Dieterlen (1963)
IW1444-21	*Acomys dimidiatus*	52.5	2 weeks	weaning	http://www.bristolzoo.org.uk/spiny-mouse [2014-05-02]
IW1448	*Apodemus agrarius*				
IW1448-01	*Apodemus agrarius*	11.5	11.5	third level	Štěrba (1977b)
IW1448-02	*Apodemus agrarius*	13.5	13.5	forth level	Štěrba (1977b)
IW1448-03	*Apodemus agrarius*	15	15	fifth level	Štěrba (1977b)
IW1448-04	*Apodemus agrarius*	17	17	sixth level	Štěrba (1977b)
IW1448-05	*Apodemus agrarius*	18	18	seventh level	Štěrba (1977b)
IW1448-06	*Apodemus agrarius*	20	20	eigth level	Štěrba (1977b)
IW1448-07	*Apodemus agrarius*	21	21	ninth level	Štěrba (1977b)
IW1448-08	*Apodemus agrarius*	22	22	birth	Štěrba (1977b)
IW1448-09	*Apodemus agrarius*	32	32	9-11d after birth: eye opening	Pelz et al. (1996)
IW1448-10	*Apodemus agrarius*	37	D15 after birth	weaning	Pelz et al. (1996)
IW1449	*Apodemus flavicollis*				
IW1449-01	*Apodemus flavicollis*	12.5	12.5	third level	Štěrba (1977b)
IW1449-02	*Apodemus flavicollis*	14	14	forth level	Štěrba (1977b)
IW1449-03	*Apodemus flavicollis*	15	15	fifth level	Štěrba (1977b)
IW1449-04	*Apodemus flavicollis*	17	17	sixth level	Štěrba (1977b)
IW1449-05	*Apodemus*	19	19	seventh level	Štěrba (1977b)

	flavicollis				
IW1449-06	*Apodemus flavicollis*	20.5	20.5	eigth level	Štěrba (1977b)
IW1449-07	*Apodemus flavicollis*	22	22	ninth level	Štěrba (1977b)
IW1449-08	*Apodemus flavicollis*	23	23	birth	Štěrba (1977b)
IW1449-09	*Apodemus flavicollis*	37.5	13-16 after birth	Eyes open	http://wildpro.twycrosszoo.org/s/0MRodenti/Muridae/apodemus/Apodemus_flavicollis/Apodemus_flavicollis.htm [2014-04-23]
IW1449-10	*Apodemus flavicollis*	54	3 weeks after birth	weaning	dito
IW1450	*Apodemus sylvaticus*				
IW1450-01	*Apodemus sylvaticus*	12.5	12.5	third level	Štěrba (1977b)
IW1450-02	*Apodemus sylvaticus*	14	14	forth level	Štěrba (1977b)
IW1450-03	*Apodemus sylvaticus*	15	15	fifth level	Štěrba (1977b)
IW1450-04	*Apodemus sylvaticus*	17	17	sixth level	Štěrba (1977b)
IW1450-05	*Apodemus sylvaticus*	19	19	seventh level	Štěrba (1977b)
IW1450-06	*Apodemus sylvaticus*	20.5	20.5	eigth level	Štěrba (1977b)
IW1450-07	*Apodemus sylvaticus*	22	22	ninth level	Štěrba (1977b)
IW1450-08	*Apodemus sylvaticus*	23	23	birth	Štěrba (1977b)
IW1450-09	*Apodemus sylvaticus*	39	39	Eye opening: 16 days after birth	http://www.the-piedpiper.co.uk/th1g.htm (2014-04-23)
IW1450-10	*Apodemus sylvaticus*	41	18d old	weaning	dito
IW1290	*Bos taurus primigenius*				
IW1290-01	*Bos taurus primigenius*	19.5	19.5	-	Evans and Sack (1973)
IW1290-02	*Bos taurus primigenius*	20.5	20.5	-	Evans and Sack (1973)
IW1290-03	*Bos taurus primigenius*	21.6	x	I	Krölling (1924)
IW1290-04	*Bos taurus primigenius*	22.7	x	II	Krölling (1924)
IW1290-05	*Bos taurus primigenius*	23.8	x	III	Krölling (1924)
IW1290-06	*Bos taurus primigenius*	24.9	x	IV	Krölling (1924)
IW1290-07	*Bos taurus primigenius*	26	x	V	Krölling (1924)
IW1290-08	*Bos taurus primigenius*	27.1	x	VI	Krölling (1924)
IW1290-09	*Bos taurus primigenius*	28	28	VII	Krölling (1924)
IW1290-10	*Bos taurus primigenius*	29.5	30	-	Evans and Sack (1973)
IW1290-11	*Bos taurus primigenius*	30.5	30	VIII	Krölling (1924)
IW1290-12	*Bos taurus primigenius*	33	33	IX	Krölling (1924)
IW1290-13	*Bos taurus primigenius*	34	x	Figure 13.11A	Rüsse and Sinowatz (1991)
IW1290-14	*Bos taurus primigenius*	35	x	Figure 1	Michl (1920)
IW1290-15	*Bos taurus primigenius*	36	36	x	Krölling (1924)
IW1290-16	*Bos taurus primigenius*	36.2	32	Figure 12.11	Rüsse and Sinowatz (1991)
IW1290-17	*Bos taurus primigenius*	36.4	x	Figure 13.11B	Rüsse and Sinowatz (1991)
IW1290-18	*Bos taurus primigenius*	36.6	x	Figure 12.15	Rüsse and Sinowatz (1991)
IW1290-19	*Bos taurus*	36.8	x	Figures 2-4	Michl (1920)

	primigenius				
IW1290-20	*Bos taurus primigenius*	37	37	Figure 12.11	Rüsse and Sinowatz (1991)
IW1290-21	*Bos taurus primigenius*	39.5	40	Figure 16.10A	Rüsse and Sinowatz (1991)
IW1290-22	*Bos taurus primigenius*	40	x	Figure 9.14	Rüsse and Sinowatz (1991)
IW1290-23	*Bos taurus primigenius*	40.5	40	-	Evans and Sack (1973)
IW1290-24	*Bos taurus primigenius*	44.5	45	Figure 12.11 / Table page 30	Rüsse and Sinowatz (1991) / Evans and Sack (1973)
IW1290-25	*Bos taurus primigenius*	45.5	45	Figure 16.10B	Rüsse and Sinowatz (1991)
IW1290-26	*Bos taurus primigenius*	49.5	50	Figure 16.10C / Table page 30	Rüsse and Sinowatz (1991) / Evans and Sack (1973)
IW1290-27	*Bos taurus primigenius*	50.5	50	Figure 12.11	Rüsse and Sinowatz (1991)
IW1290-28	*Bos taurus primigenius*	52	52	-	Evans and Sack (1973)
IW1290-29	*Bos taurus primigenius*	55	55	Figure 12.11	Rüsse and Sinowatz (1991)
IW1290-30	*Bos taurus primigenius*	56.5	x	Figure 22.19	Schnorr and Kressin (2006)
IW1290-31	*Bos taurus primigenius*	58	58	Figure 16.10D	Rüsse and Sinowatz (1991)
IW1290-32	*Bos taurus primigenius*	60	60	Table page 30	Evans and Sack (1973) / Rüsse and Sinowatz (1991)
IW1290-33	*Bos taurus primigenius*	62	62	Figure 12.11	Rüsse and Sinowatz (1991)
IW1290-34	*Bos taurus primigenius*	76	76	Table page 30	Evans and Sack (1973) / Rüsse and Sinowatz (1991)
IW1290-35	*Bos taurus primigenius*	80	80		
IW1290-36	*Bos taurus primigenius*	88	x	Figure 29.9	Schnorr and Kressin (2006)
IW1290-37	*Bos taurus primigenius*	114	3.8 months (1 month = 30days)	Figure 15.17 + 15.22 / Figure 8.11	Schnorr and Kressin (2006) / Rüsse and Sinowatz (1991)
IW1290-38	*Bos taurus primigenius*	120	4 months /1 month = 30 days	Figure 9.22	Rüsse and Sinowatz (1991)
IW1290-39	*Bos taurus primigenius*	129	4.3 months (1 month = 30days)	-	Rüsse and Sinowatz (1991)
IW1290-40	*Bos taurus primigenius*	182/180	182/180	-	Evans and Sack (1973) / Müller 1972-73 (eye opening in *Bos taurus* 180d)
IW1290-41	*Bos taurus primigenius*	230	230	Table page 30	Evans and Sack (1973) / Rüsse and Sinowatz (1991)
IW1290-42	*Bos taurus primigenius*	284	278-290d	birth: Table page 30 / Figure 9.21A	Evans and Sack (1973) / Rüsse and Sinowatz (1991)
IW1290-43	*Bos taurus primigenius*	300	postnatal (not long after birth)	Figure 9.21B	Evans and Sack (1973) / Rüsse and Sinowatz (1991)
IW1290-44	*Bos taurus primigenius*	489	Normal weaning 205days	weaning	http://beefmagazine.com/weaning/early-weaning-may-offer-no-feed-savings-advantage [2014-05-02]
IW1297	*Bradypus tridactylus*				
IW1297-01	*Bradypus tridactylus*	x	x	C. 462, figure 6-8	Heuser and Wislocki (1935)
IW1297-02	*Bradypus tridactylus*	x	x	C. 463, figure 9-10	Heuser and Wislocki (1935)
IW1297-03	*Bradypus tridactylus*	x	x	C. 464, figure 11-12	Heuser and Wislocki (1935)
IW1297-04	*Bradypus tridactylus*	x	x	C. 465, figure 13-15	Heuser and Wislocki (1935)
IW1297-05	*Bradypus tridactylus*	x	x	specimen 1: notebook 28 page 2-5; specimen 2: notebook 28, page 11-12	Embryological collection Berlin (Hill collection)
IW1297-06	*Bradypus tridactylus*	141	141	Birth, eye open, full fur	http://animaldiversity.ummz.umich.edu/accounts/Bradypus_tridactylus/ (2014-02-10), Hayssen (2009)
IW1297-07	*Bradypus tridactylus*	204	At least 9 weeks (63 days)	weaning	Hayssen (2009)
IW1299	*Callithrix jacchus ("Hapale")*				
IW1299-01	*Callithrix jacchus*	26.5	25-28	stage VII	Phillips (1976) / for Phillips (1976) see also Butler and

					Juurlink (1987)
IW1299-02	*Callithrix jacchus*	45.5	42-49	stage VIII	Phillips (1976) / for Phillips (1976) see also Butler and Juurlink (1987)
IW1299-03	*Callithrix jacchus*	55	55-59	stage IX	Phillips (1976) / for Phillips (1976) see also Butler and Juurlink (1987)
IW1299-04	*Callithrix jacchus*	56	51-59	stage X	Phillips (1976) / for Phillips (1976) see also Butler and Juurlink (1987)
IW1299-05	*Callithrix jacchus*	57	52-60	stage XI	Phillips (1976) / for Phillips (1976) see also Butler and Juurlink (1987)
IW1299-06	*Callithrix jacchus*	58.75		notebook 34, embryo A, pages 19-21	Embryological collection berlin (Hill collection)
IW1299-07	*Callithrix jacchus*	60.5	51-70	stage XIII	Phillips (1976) / for Phillips (1976) see also Butler and Juurlink (1987)
IW1299-08	*Callithrix jacchus*	61.25		notebook 34, embryo B, pages 1-18	Embryological collection berlin (Hill collection)
IW1299-09	*Callithrix jacchus*	62	61-63	stage XIV	Phillips (1976) / for Phillips (1976) see also Butler and Juurlink (1987)
IW1299-10	*Callithrix jacchus*	64.5	54-75	stage XV	Phillips (1976) / for Phillips (1976) see also Butler and Juurlink (1987)
IW1299-11	*Callithrix jacchus*	68	66-83	stage XVI	Phillips (1976) / for Phillips (1976) see also Butler and Juurlink (1987)
IW1299-12	*Callithrix jacchus*	71.5	61-77	stage XVII	Phillips (1976) / for Phillips (1976) see also Butler and Juurlink (1987)
IW1299-13	*Callithrix jacchus*	75	75	stage XIX	Phillips (1976) / for Phillips (1976) see also Butler and Juurlink (1987)
IW1299-14	*Callithrix jacchus*	80	73-87	stage XXI	Phillips (1976) / for Phillips (1976) see also Butler and Juurlink (1987)
IW1299-15	*Callithrix jacchus*	100.5		notebook 34, embryo B; pages 37-41 = figure 67	Embryological collection berlin (Hill collection) = Hill (1932)
IW1299-16	*Callithrix jacchus*	121		notebook 34, embryo A, pages 32-35.42	Embryological collection berlin (Hill collection)
IW1299-17	*Callithrix jacchus*	141.5	132-151	birth (estimated eye opening)	Hayssen et al. (1993)
IW1299-18	*Callithrix jacchus*	232.75	3 months (91.25)	weaning	http://pin.primate.wisc.edu/factsheets/entry/common_marmoset/behav [2014-05-02]
IW1306	*Canis lupus*				
IW1306-01	*Canis lupus forma familiaris*	3		figure 31	Bischoff (1845)
IW1306-02	*Canis lupus forma familiaris*	6		VI1, figure 14	Bonnet (1897)
IW1306-03	*Canis lupus forma familiaris*	9		VI2, figure 15-16	Bonnet (1897)
IW1306-04	*Canis lupus forma familiaris*	12		VII1, figure 17	Bonnet (1897)
IW1306-05	*Canis lupus forma familiaris*	15		VII2, figure 18	Bonnet (1897)
IW1306-06	*Canis lupus forma familiaris*	15.1	15	page 23	Evans and Sack (1973)
IW1306-07	*Canis lupus forma familiaris*	15.2		VIII1, figure 19	Bonnet (1897)
IW1306-08	*Canis lupus forma familiaris*	15.3		figure 32	Bischoff (1845)
IW1306-09	*Canis lupus forma familiaris*	15.4		figure 33	Bischoff (1845)
IW1306-10	*Canis lupus forma familiaris*	15.5		VIII2, figure 20	Bonnet (1897)
IW1306-11	*Canis lupus forma familiaris*	15.6		figure 34	Bischoff (1845)
IW1306-12	*Canis lupus forma familiaris*	15.7		VIII3, figure 21	Bonnet (1897)
IW1306-13	*Canis lupus forma familiaris*	15.8		VIII4, figure 22	Bonnet (1897)
IW1306-14	*Canis lupus forma familiaris*	15.9		VIII5, figure 23	Bonnet (1897)
IW1306-15	*Canis lupus forma*	16	16	page 23 / figure 35 / embryo	Evans and Sack (1973) /

	familiaris			IX1, figure 1	Bischoff (1845) / Bonnet (1901)
IW1306-16	*Canis lupus forma familiaris*	16.4		embryo IX2, figure 2	Bonnet (1901)
IW1306-17	*Canis lupus forma familiaris*	16.8		embryo X3, figure 3-4	Bonnet (1901)
IW1306-18	*Canis lupus forma familiaris*	17.2		embryo X5, figure 5-6	Bonnet (1901)
IW1306-19	*Canis lupus forma familiaris*	17.6		embryo XI6, figure 7	Bonnet (1901)
IW1306-20	*Canis lupus forma familiaris*	18		embryo XI8, figure 8	Bonnet (1901)
IW1306-21	*Canis lupus forma familiaris*	18.4		embryo I2, figure 9	Bonnet (1901)
IW1306-22	*Canis lupus forma familiaris*	18.8	0.5d after specimen in figure 35 of Bischoff (1845)	figure 36	Bischoff (1845)
IW1306-23	*Canis lupus forma familiaris*	19.2	23-24	figure 37	Bischoff (1845)
IW1306-24	*Canis lupus forma familiaris*	19.6	20	page 23	Evans and Sack (1973)
IW1306-25	*Canis lupus forma familiaris*	20		embryo XIIr2, figure 10 / figure 38	Bonnet (1901) / Bischoff (1845)
IW1306-26	*Canis lupus forma familiaris*	21		figure 39	Bischoff (1845)
IW1306-27	*Canis lupus forma familiaris*	22	0.5d after specimen in figure 39	figure 40	Bischoff (1845)
IW1306-28	*Canis lupus forma familiaris*	23		figure 41	Bischoff (1845)
IW1306-29	*Canis lupus forma familiaris*	24	20	10:29-10:55	Animals in the Womb: Dogs
IW1306-30	*Canis lupus forma familiaris*	25	25	figure 42	Bischoff (1845)
IW1306-31	*Canis lupus forma familiaris*	26		figure 43	Bischoff (1845)
IW1306-32	*Canis lupus forma familiaris*	27		figure 44	Bischoff (1845)
IW1306-33	*Canis lupus forma familiaris*	28	22	figure 8.29 / figure 57	Rüsse and Sinowatz (1991) / Keibel 1906
IW1306-34	*Canis lupus forma familiaris*	28.5	28d (4 weeks)	figure 45	Bischoff (1845)
IW1306-35	*Canis lupus forma familiaris*	29		figure 1A	Miglino et al. (2006)
IW1306-36	*Canis lupus forma familiaris*	30	30	11:49-12:54, around 15:30 / page 23	Animals in the Womb: Dogs / Evans and Sack (1973)
IW1306-37	*Canis lupus forma familiaris*	32		figure 16.6	Rüsse and Sinowatz (1991)
IW1306-38	*Canis lupus forma familiaris*	34	34	16:46-17:41, around 19:00, 20:25	Animals in the Womb: Dogs
IW1306-39	*Canis lupus forma familiaris*	35	35	page 23	Evans and Sack (1973)
IW1306-40	*Canis lupus forma familiaris*	36	36	22.15-22.52	Townend (2009)
IW1306-41	*Canis lupus forma familiaris*	45	45	figure 1B	Miglino et al. (2006)
IW1306-42	*Canis lupus forma familiaris*	55	55	33:45-35:00	Townend (2009)
IW1306-43	*Canis lupus forma familiaris*	59	59	38:38-39:33	Townend (2009)
IW1306-44	*Canis lupus forma familiaris*	60	57-63 birth (Evans and Sack 1973)	around 42:00	Townend (2009)
IW1306-45	*Canis lupus forma familiaris*	73	12-14	eye opening in the grey wolf	http://www.transylvanianwildlifeproject.com/2009/12/wolves.html [2014-04-23]
IW1306-46	*Canis lupus forma familiaris*	105	45d (wolf)	weaning	http://animaldiversity.ummz.umich.edu/accounts/Canis_lupus/ [2014-04-23]
IW1276	*Capra ("aegagrus") hircus*				
IW1276-01	*Capra hircus*	x	x	Plate 1, Figure 1	Tsukaguchi (1912)
IW1276-02	*Capra hircus*	x	x	Plate 1, Figure 2	Tsukaguchi (1912)
IW1276-03	*Capra hircus*	x	x	Plate 1, Figure 3	Tsukaguchi (1912)
IW1276-04	*Capra hircus*	x	x	Plate 1, Figure 4	Tsukaguchi (1912)
IW1276-05	*Capra hircus*	x	x	Plate 1, Figure 5	Tsukaguchi (1912)
IW1276-06	*Capra hircus*	x	x	Plate 1, Figure 6	Tsukaguchi (1912)
IW1276-07	*Capra hircus*	x	x	Plate 1, Figure 7	Tsukaguchi (1912)

IW1276-08	*Capra hircus*	x	x	Plate 1, Figure 8	Tsukaguchi (1912)
IW1276-09	*Capra hircus*	x	x	Plate 1, Figure 9	Tsukaguchi (1912)
IW1276-10	*Capra hircus*	x	x	Plate 1, Figure 10	Tsukaguchi (1912)
IW1276-11	*Capra hircus*	x	x	Plate 1, Figure 11	Tsukaguchi (1912)
IW1276-12	*Capra hircus*	x	x	Plate 1, Figure 12	Tsukaguchi (1912)
IW1276-13	*Capra hircus*	x	x	Plate 1, Figure 13	Tsukaguchi (1912)
IW1276-14	*Capra hircus*	x	x	Plate 1, Figure 14	Tsukaguchi (1912)
IW1276-15	*Capra hircus*	160	150-170	birth (estimated eye opening)	Hayssen et al. (1993)
IW1276-16	*Capra hircus*	464.17	10 months after birth (304.17)	weaning	http://animaldiversity.ummz.umich.edu/accounts/Capra_hircus/ [2014-04-23]
IW1307	*Capreolus capreolus*				
IW1307-01	*Capreolus capreolus*	x	x	figure 1	Sakurai (1906)
IW1307-02	*Capreolus capreolus*	x	x	figure 2	Sakurai (1906)
IW1307-03	*Capreolus capreolus*	x	x	figure 3	Sakurai (1906)
IW1307-04	*Capreolus capreolus*	x	x	figure 4	Sakurai (1906)
IW1307-05	*Capreolus capreolus*	x	x	figure 5	Sakurai (1906)
IW1307-06	*Capreolus capreolus*	x	x	figure 6	Sakurai (1906)
IW1307-07	*Capreolus capreolus*	x	x	figure 7	Sakurai (1906)
IW1307-08	*Capreolus capreolus*	x	x	figure 8	Sakurai (1906)
IW1307-09	*Capreolus capreolus*	x	x	figure 9	Sakurai (1906)
IW1307-10	*Capreolus capreolus*	x	x	figure 10	Sakurai (1906)
IW1307-11	*Capreolus capreolus*	x	x	figure 11	Sakurai (1906)
IW1307-12	*Capreolus capreolus*	x	x	figure 12	Sakurai (1906)
IW1307-13	*Capreolus capreolus*	x	x	figure 13	Sakurai (1906)
IW1307-14	*Capreolus capreolus*	x	x	figure 14	Sakurai (1906)
IW1307-15	*Capreolus capreolus*	x	x	figure 15	Sakurai (1906)
IW1307-16	*Capreolus capreolus*	x	x	figure 16	Sakurai (1906)
IW1307-17	*Capreolus capreolus*	x	x	figure 17	Sakurai (1906)
IW1307-18	*Capreolus capreolus*	x	x	figure 18	Sakurai (1906)
IW1307-19	*Capreolus capreolus*	x	x	figure 19	Sakurai (1906)
IW1307-20	*Capreolus capreolus*	x	x	figure 20 / plate 24	Sakurai (1906) / Retzius (1900)
IW1307-21	*Capreolus capreolus*	x	x	figure 21	Sakurai (1906)
IW1307-22	*Capreolus capreolus*	x	x	figure 22	Sakurai (1906)
IW1307-23	*Capreolus capreolus*	x	x	figure 23 / figure 55a	Sakurai (1906) / Keibel (1906)
IW1307-24	*Capreolus capreolus*	x	x	figure 24	Sakurai (1906)
IW1307-25	*Capreolus capreolus*	x	x	figure 25	Sakurai (1906)
IW1307-26	*Capreolus capreolus*	x	x	figure 26	Sakurai (1906)
IW1307-27	*Capreolus capreolus*	x	x	figure 27	Sakurai (1906)
IW1307-28	*Capreolus capreolus*	x	x	figure 28 / figure 55b	Sakurai (1906)
IW1307-29	*Capreolus capreolus*	x	x	figure 29	Sakurai (1906)
IW1307-30	*Capreolus capreolus*	x	x	figure 30	Sakurai (1906)
IW1307-31	*Capreolus capreolus*	x	x	figure 31 / figure 55c	Sakurai (1906) / Keibel (1906)
IW1307-32	*Capreolus*	x	x	figure 32	Sakurai (1906)

	capreolus				
IW1307-33	*Capreolus capreolus*	x	x	figure 33	Sakurai (1906)
IW1307-34	*Capreolus capreolus*	x	x	figure 34	Sakurai (1906)
IW1307-35	*Capreolus capreolus*	x	x	figure 35	Sakurai (1906)
IW1307-36	*Capreolus capreolus*	x	x	figure 36	Sakurai (1906)
IW1307-37	*Capreolus capreolus*	x	x	figure 37	Sakurai (1906)
IW1307-38	*Capreolus capreolus*	249	294	birth (estimated eye opening)	Hayssen et al. (1993)
IW1307-39	*Capreolus capreolus*	349	100 days after birth	weaning	http://wildpro.twycrosszoo.org/S/00Man/MammalHusbandryTechniques/UKMHusbIndTech/HR_M_Deer.htm [2014-04-23]
IW1302	*Carollia perspicillata*				
IW1302-01	*Carollia perspicillata*	0	0 (fertilization)	stage 1	Cretekos et al. (2005)
IW1302-02	*Carollia perspicillata*	3.3	x	stage 2	Cretekos et al. (2005)
IW1302-03	*Carollia perspicillata*	6.7	x	stage 3	Cretekos et al. (2005)
IW1302-04	*Carollia perspicillata*	10	x	stage 4	Cretekos et al. (2005)
IW1302-05	*Carollia perspicillata*	13.6	x	stage 5	Cretekos et al. (2005)
IW1302-06	*Carollia perspicillata*	16.7	x	stage 6	Cretekos et al. (2005)
IW1302-07	*Carollia perspicillata*	20,	x	stage 7	Cretekos et al. (2005)
IW1302-08	*Carollia perspicillata*	23.3	x	stage 8	Cretekos et al. (2005)
IW1302-09	*Carollia perspicillata*	26.7	x	stage 9	Cretekos et al. (2005)
IW1302-10	*Carollia perspicillata*	30	x	stage 10	Cretekos et al. (2005)
IW1302-11	*Carollia perspicillata*	33.3	x	stage 11	Cretekos et al. (2005)
IW1302-12	*Carollia perspicillata*	36.7	x	stage 12	Cretekos et al. (2005)
IW1302-13	*Carollia perspicillata*	40	x	stage 13	Cretekos et al. (2005)
IW1302-14	*Carollia perspicillata*	44	44	stage 14	Cretekos et al. (2005)
IW1302-15	*Carollia perspicillata*	46	46	stage 15	Cretekos et al. (2005)
IW1302-16	*Carollia perspicillata*	50	50	stage 16	Cretekos et al. (2005)
IW1302-17	*Carollia perspicillata*	54	54	stage 17	Cretekos et al. (2005)
IW1302-18	*Carollia perspicillata*	60	60	stage 18	Cretekos et al. (2005)
IW1302-19	*Carollia perspicillata*	65		stage 19	Cretekos et al. (2005)
IW1302-20	*Carollia perspicillata*	70	70	stage 20	Cretekos et al. (2005)
IW1302-21	*Carollia perspicillata*	75		stage 21	Cretekos et al. (2005)
IW1302-22	*Carollia perspicillata*	80	80	stage 22	Cretekos et al. (2005)
IW1302-23	*Carollia perspicillata*	85	83-87	stage 23	Cretekos et al. (2005)
IW1302-24	*Carollia perspicillata*	90	90	stage 24, fetal period	Cretekos et al. (2005)
IW1302-25	*Carollia perspicillata*	115	105-125	Birth	Laska (1990)
IW1302-26	*Carollia perspicillata*	187	72 days after birth	weaning	Cloutier and Thomas (1992)
IW1286	*Cavia porcellus*				
IW1286-01	*Cavia porcellus*	12	14+d	3	Harman and Prickett Dobrovolny (1933), age calculated and estimated after

					Scott (1937)
IW1286-02	*Cavia porcellus*	12.73	12d17h35min	3	Scott (1937)
IW1286-03	*Cavia porcellus*	13.47	13d11h22min	4	Scott (1937)
IW1286-04	*Cavia porcellus*	13.72	14d	4	Harman and Prickett Dobrovolny (1933), age calculated and estimated after Scott (1937)
IW1286-05	*Cavia porcellus*	13.98	17	41.42	Bischoff (1852), age calculated and estimated after Scott (1937)
IW1286-06	*Cavia porcellus*	14.24	N.N.	5, 6	Harman and Prickett Dobrovolny (1933), age calculated and estimated after Scott (1937)
IW1286-07	*Cavia porcellus*	14.49	14d11h44min	5	Scott (1937)
IW1286-08	*Cavia porcellus*	14.66	early 14d	7	Harman and Prickett Dobrovolny (1933), age calculated and estimated after Scott (1937)
IW1286-09	*Cavia porcellus*	14.84	15	8	Harman and Prickett Dobrovolny (1933), age calculated and estimated after Scott (1937)
IW1286-10	*Cavia porcellus*	15	18	46.47.48.49	Bischoff (1852), age calculated and estimated after Scott (1937)
IW1286-11	*Cavia porcellus*	15.17	15	9	Harman and Prickett Dobrovolny (1933), age calculated and estimated after Scott (1937)
IW1286-12	*Cavia porcellus*	15.35	15+	10	Harman and Prickett Dobrovolny (1933), age calculated and estimated after Scott (1937)
IW1286-13	*Cavia porcellus*	15.53	15d12h45min	6	Scott (1937)
IW1286-14	*Cavia porcellus*	15.77	early 15	11	Harman and Prickett Dobrovolny (1933), age calculated and estimated after Scott (1937)
IW1286-15	*Cavia porcellus*	16	21	53, 54	Bischoff (1852), age calculated and estimated after Scott (1937)
IW1286-16	*Cavia porcellus*	16.26	13-14d	55	Bischoff (1852), age calculated and estimated after Scott (1937)
IW1286-17	*Cavia porcellus*	16.5	16d12h6min	7	Scott (1937)
IW1286-18	*Cavia porcellus*	17.53	17d12h40min	8	Scott (1937)
IW1286-19	*Cavia porcellus*	17.73	16	12	Harman and Prickett Dobrovolny (1933), age calculated and estimated after Scott (1937)
IW1286-20	*Cavia porcellus*	17.92	17	13	Harman and Prickett Dobrovolny (1933), age calculated and estimated after Scott (1937)
IW1286-21	*Cavia porcellus*	18.19	18	14	Harman and Prickett Dobrovolny (1933), age calculated and estimated after Scott (1937)
IW1286-22	*Cavia porcellus*	18.31	22	56, 57	Bischoff (1852), age calculated and estimated after Scott (1937)
IW1286-23	*Cavia porcellus*	18.51	18d12h15min	9	Scott (1937)
IW1286-24	*Cavia porcellus*	19.52	19d12h24min	10	Scott (1937)
IW1286-25	*Cavia porcellus*	19.7	19d16h55min	11	Scott (1937)
IW1286-26	*Cavia porcellus*	20.04	19	15	Harman and Prickett Dobrovolny (1933), age calculated and estimated after Scott (1937)
IW1286-27	*Cavia porcellus*	20.38	20	16	Harman and Prickett Dobrovolny (1933), age calculated and estimated after Scott (1937)
IW1286-28	*Cavia porcellus*	20.72	20d17h10min	12	Scott (1937)
IW1286-29	*Cavia porcellus*	21.03	22	60, 61	Bischoff (1852), age calculated and estimated after Scott (1937)

IW1286-30	*Cavia porcellus*	21.33	16	63	Bischoff (1852), age calculated and estimated after Scott (1937)
IW1286-31	*Cavia porcellus*	21.64	21d15h15min	13	Scott (1937)
IW1286-32	*Cavia porcellus*	23.67	23d16h5min	14	Scott (1937)
IW1286-33	*Cavia porcellus*	26.16	26d3h45min	15	Scott (1937)
IW1286-34	*Cavia porcellus*	28	4 weeks	65-67	Bischoff (1852), age calculated and estimated after Scott (1937)
IW1286-35	*Cavia porcellus*	67	almost birth (Bischoff 1852), (67d - wikipedia.de)	68-69	Bischoff (1852), age calculated and estimated after Scott (1937)
IW1286-36	*Cavia porcellus*	68	68	birth (estimated eye opening)	Evans and Sack (1973)
IW1286-37	*Cavia porcellus*	85.5	14-21 days after birth	weaning	http://animaldiversity.ummz.umich.edu/accounts/Cavia_porcellus/ [2014-05-02]
IW1310	*Cricetulus barabensis ("griseus")*		d		
IW1310-01	*Cricetulus barabensis*	10	10	stage 9	ten Donkelaar et al. 1979 / Butler and Juurlink 1987
IW1310-02	*Cricetulus barabensis*	10.75	10.5-11	stage 10	ten Donkelaar et al. 1979 / Butler and Juurlink 1987
IW1310-03	*Cricetulus barabensis*	10.875	10.75-11	stage 11	ten Donkelaar et al. 1979 / Butler and Juurlink 1987
IW1310-04	*Cricetulus barabensis*	11.25	11-11.5	stage 12	ten Donkelaar et al. 1979 / Butler and Juurlink 1987
IW1310-05	*Cricetulus barabensis*	11.75	11.5-12	stage 13	ten Donkelaar et al. 1979 / Butler and Juurlink 1987
IW1310-06	*Cricetulus barabensis*	12.25	12-12.5	stage 14	ten Donkelaar et al. 1979 / Butler and Juurlink 1987
IW1310-07	*Cricetulus barabensis*	12.75	12.5-13	stage 15	ten Donkelaar et al. 1979 / Butler and Juurlink 1987
IW1310-08	*Cricetulus barabensis*	13	13	stage 16	ten Donkelaar et al. 1979 / Butler and Juurlink 1987
IW1310-09	*Cricetulus barabensis*	14	14	stage 17	ten Donkelaar et al. 1979 / Butler and Juurlink 1987
IW1310-10	*Cricetulus barabensis*	15.125	15-15.25	stage 18	ten Donkelaar et al. 1979 / Butler and Juurlink 1987
IW1310-11	*Cricetulus barabensis*	15.875	15.75-16	stage 19	ten Donkelaar et al. 1979 / Butler and Juurlink 1987
IW1310-12	*Cricetulus barabensis*	17	17	fetal stage 1	ten Donkelaar et al. 1979 / Butler and Juurlink 1987
IW1310-13	*Cricetulus barabensis*	18	18	fetal stage 2	ten Donkelaar et al. 1979 / Butler and Juurlink 1987
IW1310-14	*Cricetulus barabensis*	20.5	20-21	fetal stage 3, birth	ten Donkelaar et al. 1979 / Butler and Juurlink 1987
IW1310-15	*Cricetulus barabensis*	32.5	10-14d after birth	Eye opening	National Research Concil (1995)
IW1310-16	*Cricetulus barabensis*	40.5	20 days	weaning	http://eol.org/pages/1179507/data [2014-05-02]
IW1447	*Crocidura russela*				
IW1447-01	*Crocidura russela*	20	20	Fig. 3, listed by days	Vogel (1972), summarized by Štěrba (1977a)
IW1447-02	*Crocidura russela*	22	22	Fig. 3, listed by days	Vogel (1972), summarized by Štěrba (1977a)
IW1447-03	*Crocidura russela*	25	25	Fig. 3, listed by days	Vogel (1972), summarized by Štěrba (1977a)
IW1447-04	*Crocidura russela*	28	28	Fig. 3, listed by days	Vogel (1972), summarized by Štěrba (1977a)
IW1447-05	*Crocidura russela*	31	31, birth	Fig. 3, listed by days; birth	Vogel (1972), summarized by Štěrba (1977a)
IW1447-06	*Crocidura russela*	32	32	Fig. 3, listed by days	Vogel (1972), summarized by Štěrba (1977a)
IW1447-07	*Crocidura russela*	33	33	Fig. 3, listed by days	Vogel (1972), summarized by Štěrba (1977a)
IW1447-08	*Crocidura russela*	34	34	Fig. 3, listed by days	Vogel (1972), summarized by Štěrba (1977a)
IW1447-09	*Crocidura russela*	35	35	Fig. 3, listed by days	Vogel (1972), summarized by Štěrba (1977a)
IW1447-10	*Crocidura russela*	36	36	Fig. 3, listed by days	Vogel (1972), summarized by Štěrba (1977a)
IW1447-11	*Crocidura russela*	44	13d after birth	Eye opening	http://wildpro.twycrosszoo.org/s/0MInsectivor/Soricidae/Crocidura/Crocidura_russala/Crocidura_russala.htm [2014-

					04-23]
IW1447-12	*Crocidura russela*	47,5	13-20 days after birth	weaning	http://animaldiversity.ummz.umich.edu/accounts/Crocidura_russula/ [2014-04-23]
IW1251	*Dasypus hybridus*			Uterus (“Keimblase”)-Number / Figure of whole Embryo / Textfigur	
IW1251-01	*Dasypus hybridus*	x	x	96 / 38 / x	Fernandez (1915)
IW1251-02	*Dasypus hybridus*	x	x	43 / 39 / x	Fernandez (1915)
IW1251-03	*Dasypus hybridus*	x	x	103 / 3, 40 / x	Fernandez (1915)
IW1251-04	*Dasypus hybridus*	x	x	174 / 42, 43 / x	Fernandez (1915)
IW1251-05	*Dasypus hybridus*	x	x	175 /41 / x	Fernandez (1915)
IW1251-06	*Dasypus hybridus*	x	x	148 / 44-48 / x	Fernandez (1915)
IW1251-07	*Dasypus hybridus*	x	x	99 / x / 2-10	Fernandez (1915)
IW1251-08	*Dasypus hybridus*	x	x	46 / 1.2 / 11	Fernandez (1915)
IW1251-09	*Dasypus hybridus*	x	x	109 / x / 12-18	Fernandez (1915)
IW1251-10	*Dasypus hybridus*	x	x	178 / x / 19-26	Fernandez (1915)
IW1251-11	*Dasypus hybridus*	x	x	177 / 49-50 / x	Fernandez (1915)
IW1251-12	*Dasypus hybridus*	x	x	42 / 10 / 27-30	Fernandez (1915)
IW1251-13	*Dasypus hybridus*	x	x	220 / 11 / 31	Fernandez (1915)
IW1251-14	*Dasypus hybridus*	x	x	94 / 12.13 / 32, 33	Fernandez (1915)
IW1251-15	*Dasypus hybridus*	x	x	226 / 4, 14 / 34	Fernandez (1915)
IW1251-16	*Dasypus hybridus*	x	x	4 /17 / x	Fernandez (1915)
IW1251-17	*Dasypus hybridus*	x	x	199 / 5.15.16 / 35	Fernandez (1915)
IW1251-18	*Dasypus hybridus*	x	x	8 / 18 / 36-40	Fernandez (1915)
IW1251-19	*Dasypus hybridus*	x	x	188 / 19-22 / 41-46	Fernandez (1915)
IW1251-20	*Dasypus hybridus*	x	x	176 / 6, 23 / x	Fernandez (1915)
IW1251-21	*Dasypus hybridus*	x	x	11 / x / 47-48	Fernandez (1915)
IW1251-22	*Dasypus hybridus*	x	x	58 / 25 /49	Fernandez (1915)
IW1251-23	*Dasypus hybridus*	x	x	179 / 7, 24 / x	Fernandez (1915)
IW1251-24	*Dasypus hybridus*	x	x	136 / 26, 27 / 58-65	Fernandez (1915)
IW1251-25	*Dasypus hybridus*	x	x	180 / 8, 28 / 66-77	Fernandez (1915)
IW1251-26	*Dasypus hybridus*	x	x	124 / 29 / 78-87	Fernandez (1915)
IW1251-27	*Dasypus hybridus*	x	x	10 / x / 88-93	Fernandez (1915)
IW1251-28	*Dasypus hybridus*	x	x	222 / 9, 30 / 94-104	Fernandez (1915)
IW1251-29	*Dasypus hybridus*	x	x	150 / 31 / 105-115, 128	Fernandez (1915)
IW1251-30	*Dasypus hybridus*	x	x	28 /32 / 129	Fernandez (1915)
IW1251-31	*Dasypus hybridus*	x	x	185 /33 / 116-130	Fernandez (1915)
IW1251-32	*Dasypus hybridus*	x	x	206 / 34 / 117, 131	Fernandez (1915)
IW1251-33	*Dasypus hybridus*	x	x	30 / 35 / 118-126, 133	Fernandez (1915)
IW1251-34	*Dasypus hybridus*	x	x	81 / 36 / x	Fernandez (1915)
IW1251-35	*Dasypus hybridus*	x	x	24 / 37 / x	Fernandez (1915)
IW1251-36	*Dasypus hybridus*	120	4 months	birth (estimated eye opening)	Hayssen et al. (1993)
IW1251-37	*Dasypus hybridus*	255	4-5 month after birth	weaning	http://animaldiversity.ummz.umich.edu/accounts/Dasypus_hybridus/ [2014-05-02]
IW1285	*Delphinus delphis*				
IW1285-01	*Delphinus delphis*	12.75	13	as for *Stenella attenuata*	Thewissen and Heyning (2007) / Štěrba et al. (2000)
IW1285-02	*Delphinus delphis*	13.25	13	as for *Stenella attenuata*	Thewissen and Heyning (2007) / Štěrba et al. (2000)
IW1285-03	*Delphinus delphis*	13.75	14	as for *Stenella attenuata*	Thewissen and Heyning (2007) / Štěrba et al. (2000)
IW1285-04	*Delphinus delphis*	14	14	as for *Stenella attenuata*	Thewissen and Heyning (2007) / Štěrba et al. (2000)
IW1285-05	*Delphinus delphis*	14.25	14	as for *Stenella attenuata*	Thewissen and Heyning (2007) / Štěrba et al. (2000)
IW1285-06	*Delphinus delphis*	23.5	22-28, mean=24	as for *Stenella attenuata*	Thewissen and Heyning (2007) / Štěrba et al. (2000)
IW1285-07	*Delphinus delphis*	24.5	22-28, mean=24	as for *Stenella attenuata*	Thewissen and Heyning (2007) / Štěrba et al. (2000)
IW1285-08	*Delphinus delphis*	30	27-36, mean=30	as for *Stenella attenuata*	Thewissen and Heyning (2007) / Štěrba et al. (2000)
IW1285-09	*Delphinus delphis*	38	32-42, mean=38	as for *Stenella attenuata*	Thewissen and Heyning (2007) / Štěrba et al. (2000)
IW1285-10	*Delphinus delphis*	45.5	41-52, mean=46	as for *Stenella attenuata*	Thewissen and Heyning (2007) / Štěrba et al. (2000)
IW1285-11	*Delphinus delphis*	46.5	41-52, mean=46	as for *Stenella attenuata*	Thewissen and Heyning (2007) / Štěrba et al. (2000)
IW1285-12	*Delphinus delphis*	57	51-66, mean=57	as for *Stenella attenuata*	Thewissen and Heyning (2007) / Štěrba et al. (2000)
IW1285-13	*Delphinus delphis*	70	62-78, mean=70	as for *Stenella attenuata*	Thewissen and Heyning (2007) / Štěrba et al. (2000)
IW1285-14	*Delphinus delphis*	89	78-100	as for *Stenella attenuata*	Thewissen and Heyning (2007) / Štěrba et al. (2000)

IW1285-15	*Delphinus delphis*	92.5	85-100	as for *Stenella attenuata*	Thewissen and Heyning (2007) / Štĕrba et al. (2000)
IW1285-16	*Delphinus delphis*	142.5	110-175	as for *Stenella attenuata*	Thewissen and Heyning (2007) / Štĕrba et al. (2000)
IW1285-17	*Delphinus delphis*	222.5	175-270	as for *Stenella attenuata*	Thewissen and Heyning (2007) / Štĕrba et al. (2000)
IW1285-18	*Delphinus delphis*	280	270-290, birth	as for *Stenella attenuata*	Thewissen and Heyning (2007) / Štĕrba et al. (2000)
IW1285-19	*Delphinus delphis*	553.75	6-12 months after birth (9months average)	weaning	http://www.horta.uac.pt/projectos/golfinicho/paginas/species.en.html [2014-05-02]
IW1271	*Echinops telfairi*				
IW1271-01	*Echinops telfairi*	38.5	39d (circa)	Figure 2-1	Werneburg et al. (2013)
IW1271-02	*Echinops telfairi*	39.5	39d	Figure 2-2	Werneburg et al. (2013)
IW1271-03	*Echinops telfairi*	40	x	Figure 2-3	Werneburg et al. (2013)
IW1271-04	*Echinops telfairi*	40.5	x	Figure 2-4	Werneburg et al. (2013)
IW1271-05	*Echinops telfairi*	41	41d	Figure 2-5	Werneburg et al. (2013)
IW1271-06	*Echinops telfairi*	43.16	x	Figure 2-6	Werneburg et al. (2013)
IW1271-07	*Echinops telfairi*	45.33	x	Figure 2-7	Werneburg et al. (2013)
IW1271-08	*Echinops telfairi*	47.5	48	Figure 2-8	Werneburg et al. (2013)
IW1271-09	*Echinops telfairi*	48.5	48	Figure 2-9	Werneburg et al. (2013)
IW1271-10	*Echinops telfairi*	49	49	birth	Hayssen et al. (1993)
IW1271-11	*Echinops telfairi*	72,5	71-74d	Eye opening	Müller (1972)
IW1271-12	*Echinops telfairi*	78	29 days after birth	weaning	http://genomics.senescence.info/species/entry.php?species=Echinops_telfairi [2014-05-02]
IW1327	*Elephas maximus ("indicus")*			movie sequence begins as (time)	
IW1327-01	*Elephas maximus*	77	week 11 (40.05)	40:05	Abbas (2006)
IW1327-02	*Elephas maximus*	98	week 14 (40.25min)	40:25	Abbas (2006)
IW1327-03	*Elephas maximus*	112	week 16 (42:35)	42:35	Abbas (2006)
IW1327-04	*Elephas maximus*	120	4 months (1.08h)	1:08:00	Abbas (2006)
IW1327-05	*Elephas maximus*	126	week 18 (1:09:47.1.10.50h, 1.11.40)	1:09:47, 1:10:50, 1:11:40	Abbas (2006)
IW1327-06	*Elephas maximus*	133	week 19 (1.12.11h, 1.13.10)	1:12:11, 1:13:10	Abbas (2006)
IW1327-07	*Elephas maximus*	365	1year (1.15h, 1.16.45h)	1:15:00, 1:16:45	Abbas (2006)
IW1327-08	*Elephas maximus*	390	13 months (1.17.25, 1.20.40, 1.21.35h)	1:17:25, 1:20:40, 1:21:35	Abbas (2006)
IW1327-09	*Elephas maximus*	570	19 months (1.21.50, 1.22.30h)	1:21:50, 1:22:30	Abbas (2006)
IW1327-10	*Elephas maximus*	660	22 months, birth	1:23:30	Abbas (2006)
IW1327-11	*Elephas maximus*	1208	548	weaning	http://genomics.senescence.info/species/entry.php?species=Elephas_maximus [2014-05-02]
IW1289	*Equus caballus forma domestica ("ferus caballus")*		days		
IW1289-01	*Equus caballus forma domestica*	7.5	x	Figure 3	Ewart (1917)
IW1289-02	*Equus caballus forma domestica*	15	15		Evans and Sack (1973)
IW1289-03	*Equus caballus forma domestica*	15.1	13: end of second week (originally 21d after Bonnet)	Figure 3	Ewart (1917)
IW1289-04	*Equus caballus forma domestica*	17.5	17.5: middle of third week (originally 21d after Martin)	Figure 4	Ewart (1917)
IW1289-05	*Equus caballus forma domestica*	19.25	x	Figure 4	Ewart (1917)
IW1289-06	*Equus caballus forma domestica*	21	21d2h (Highland mare; after 7th day of oestrus)	Figure 5	Ewart (1917)
IW1289-07	*Equus caballus forma domestica*	21.11	21	Figure 7 / 52	Martin (1890): cited in Ewart (1917) / Keibel (1906)
IW1289-08	*Equus caballus forma domestica*	21.22	17	Figure 2 - day 17	Acker et al. (2001)
IW1289-09	*Equus caballus forma domestica*	21.33	18	Figure 2 - day 18	Acker et al. (2001)
IW1289-10	*Equus caballus forma domestica*	21.44	21	Figure 2a	Vitums (1969)
IW1289-11	*Equus caballus forma domestica*	21.55	19	Figure 2 - day 19	Acker et al. (2001)
IW1289-12	*Equus caballus*	21.66	20	Figure 2 - day 20	Acker et al. (2001)

	forma domestica				
IW1289-13	*Equus caballus forma domestica*	21.77	21 / 21.5 +/-0.5	Figures 8-11, 34-35 / Figure 40	Ewart (1917) / Betteridge et al. (1982)
IW1289-14	*Equus caballus forma domestica*	21.88	21	Plate 19	Robinson and Gibson (1917)
IW1289-15	*Equus caballus forma domestica*	22	22	Figure 2 - day 22	Acker et al. (2001)
IW1289-16	*Equus caballus forma domestica*	23	23	Figure 2b	Vitums (1969)
IW1289-17	*Equus caballus forma domestica*	24	24	Figure 2 - day 24	Acker et al. (2001)
IW1289-18	*Equus caballus forma domestica*	25.5	26	-	Evans and Sack (1973)
IW1289-19	*Equus caballus forma domestica*	26.5	26	Figure 2 - day 26	Acker et al. (2001)
IW1289-20	*Equus caballus forma domestica*	27	27	Figure 3	Vitums (1969)
IW1289-21	*Equus caballus forma domestica*	27.5	28	Figure 3	Ewart (1917)
IW1289-22	*Equus caballus forma domestica*	28	x	Figure 1	Harrison and Mohn (1932)
IW1289-23	*Equus caballus forma domestica*	28.5	28	Figure 3	Ewart (1917)
IW1289-24	*Equus caballus forma domestica*	29.5	30	-	Schnorr and Kressin (2006)
IW1289-25	*Equus caballus forma domestica*	30	30	Figure 2 - day 30	Acker et al. (2001)
IW1289-26	*Equus caballus forma domestica*	30.5	30	Figure 4	Vitums (1969)
IW1289-27	*Equus caballus forma domestica*	32	32	Figure 5	Vitums (1969)
IW1289-28	*Equus caballus forma domestica*	33	x	Figure 2	Harrison and Mohn (1932)
IW1289-29	*Equus caballus forma domestica*	34	34	Figure 6	Vitums (1969)
IW1289-30	*Equus caballus forma domestica*	34.67	35	Figure 4	Ewart (1917)
IW1289-31	*Equus caballus forma domestica*	35.33	35	Figure 7	Vitums (1969)
IW1289-32	*Equus caballus forma domestica*	36	36	Figure 8	Vitums (1969)
IW1289-33	*Equus caballus forma domestica*	36.67	35	-	Evans and Sack (1973)
IW1289-34	*Equus caballus forma domestica*	38	38	Figure 9	Vitums (1969)
IW1289-35	*Equus caballus forma domestica*	37.33	36	Figure 2 - day 36	Acker et al. (2001)
IW1289-36	*Equus caballus forma domestica*	40	40	Figure 2 - day 40	Acker et al. (2001)
IW1289-37	*Equus caballus forma domestica*	41.5	42	Figure 5	Ewart (1917)
IW1289-38	*Equus caballus forma domestica*	42.5	42	Figure 10	Vitums (1969)
IW1289-39	*Equus caballus forma domestica*	49	49	Figure 6	Ewart (1917)
IW1289-40	*Equus caballus forma domestica*	55	55	-	Evans and Sack (1973)
IW1289-41	*Equus caballus forma domestica*	46	56	Figure 7	Ewart (1917)
IW1289-42	*Equus caballus forma domestica*	80	80	Figure 15.4	Schnorr and Kressin 2006
IW1289-43	*Equus caballus forma domestica*	95	95 (before end of 14th week)	Figure 8.24	Rüsse and Sinowatz (1991)
IW1289-44	*Equus caballus forma domestica*	112	112	Table page 26	Evans and Sack (1973)
IW1289-45	*Equus caballus forma domestica*	180	180	Table page 26	Evans and Sack (1973)
IW1289-46	*Equus caballus forma domestica*	240	240	Table page 26	Evans and Sack (1973)
IW1289-47	*Equus caballus forma domestica*	270	270	Table page 26	Evans and Sack (1973)
IW1289-48	*Equus caballus forma domestica*	335	335	birth: Table page 26 (estimated eye opening)	Evans and Sack (1973)
IW1289-49	*Equus caballus forma domestica*	609	274d after birth	weaning	http://genomics.senescence.info/species/entry.php?species=Equus_caballus [2014-05-02]

IW1312	*Erinaceus europaeus*				
IW1312-01	*Erinaceus europaeus*	x	x	figure 3 (blastoporus)	Petermann (1907)
IW1312-02	*Erinaceus europaeus*	x	x	figures 6-9 (primitive streak)	Petermann (1907)
IW1312-03	*Erinaceus europaeus*	x	x	figure 10-12 (21-2 somite)	Petermann (1907)
IW1312-04	*Erinaceus europaeus*	x	x	figure 1, embryo 1	Jacobfeuerborn (1908)
IW1312-05	*Erinaceus europaeus*	x	x	figure 28, embryo 27	Jacobfeuerborn (1908)
IW1312-06	*Erinaceus europaeus*	x	x	figures 2+29, embryo 2	Jacobfeuerborn (1908)
IW1312-07	*Erinaceus europaeus*	x	x	figure 30, embryo 28	Jacobfeuerborn (1908)
IW1312-08	*Erinaceus europaeus*	x	x	figure 31, embryo 29	Jacobfeuerborn (1908)
IW1312-09	*Erinaceus europaeus*	x	x	figure 3+32, embryo 32	Jacobfeuerborn (1908)
IW1312-10	*Erinaceus europaeus*	x	x	figure 33, embryo 30	Jacobfeuerborn (1908)
IW1312-11	*Erinaceus europaeus*	x	x	figure 34, embryo 31	Jacobfeuerborn (1908)
IW1312-12	*Erinaceus europaeus*	x	x	figure 35, embryo 32	Jacobfeuerborn (1908)
IW1312-13	*Erinaceus europaeus*	x	x	figure 36, embryo 33	Jacobfeuerborn (1908)
IW1312-14	*Erinaceus europaeus*	x	x	textfigure 1, embryo 34	Jacobfeuerborn (1908)
IW1312-15	*Erinaceus europaeus*	x	x	figure 4, embryo 4	Jacobfeuerborn (1908)
IW1312-16	*Erinaceus europaeus*	x	x	figure 37, embryo 35	Jacobfeuerborn (1908)
IW1312-17	*Erinaceus europaeus*	x	x	figure 5, embryo 5	Jacobfeuerborn (1908)
IW1312-18	*Erinaceus europaeus*	x	x	figure 6, embryo 6	Jacobfeuerborn (1908)
IW1312-19	*Erinaceus europaeus*	x	x	figure 7, embryo 7	Jacobfeuerborn (1908)
IW1312-20	*Erinaceus europaeus*	x	x	figure 8, embryo 8	Jacobfeuerborn (1908)
IW1312-21	*Erinaceus europaeus*	x	x	figure 9, embryo 9	Jacobfeuerborn (1908)
IW1312-22	*Erinaceus europaeus*	x	x	figure 10, embryo 10	Jacobfeuerborn (1908)
IW1312-23	*Erinaceus europaeus*	x	x	figure 11, embryo 11	Jacobfeuerborn (1908)
IW1312-24	*Erinaceus europaeus*	x	x	figure 12, embryo 12	Jacobfeuerborn (1908)
IW1312-25	*Erinaceus europaeus*	x	x	figure 38, embryo 36	Jacobfeuerborn (1908)
IW1312-26	*Erinaceus europaeus*	x	x	figures 13+39, embryo 13	Jacobfeuerborn (1908)
IW1312-27	*Erinaceus europaeus*	x	x	figure 40, embryo 17	Jacobfeuerborn (1908)
IW1312-28	*Erinaceus europaeus*	x	x	figure 14, embryo 14	Jacobfeuerborn (1908)
IW1312-29	*Erinaceus europaeus*	x	x	figure 15+41, embryo 15	Jacobfeuerborn (1908)
IW1312-30	*Erinaceus europaeus*	x	x	figure 16, embryo 16	Jacobfeuerborn (1908)
IW1312-31	*Erinaceus europaeus*	x	x	figure 42, embryo 38	Jacobfeuerborn (1908)
IW1312-32	*Erinaceus europaeus*	x	x	figure 17, embryo 43	Jacobfeuerborn (1908)
IW1312-33	*Erinaceus europaeus*	x	x	figure 18, embryo 18	Jacobfeuerborn (1908)
IW1312-34	*Erinaceus europaeus*	x	x	figure 19, embryo 19	Jacobfeuerborn (1908)
IW1312-35	*Erinaceus europaeus*	x	x	figure 20, embryo 20	Jacobfeuerborn (1908)
IW1312-36	*Erinaceus europaeus*	x	x	figure 21, embryo 21	Jacobfeuerborn (1908)
IW1312-37	*Erinaceus europaeus*	x	x	figure 44, embryo 39	Jacobfeuerborn (1908)

IW1312-38	*Erinaceus europaeus*	x	x	figure 22, embryo 22	Jacobfeuerborn (1908)
IW1312-39	*Erinaceus europaeus*	x	x	figure 23, embryo 23	Jacobfeuerborn (1908)
IW1312-40	*Erinaceus europaeus*	x	x	figure 24, embryo 24	Jacobfeuerborn (1908)
IW1312-41	*Erinaceus europaeus*	x	x	figure 25, embryo 25	Jacobfeuerborn (1908)
IW1312-42	*Erinaceus europaeus*	x	x	figure 26, embryo 26	Jacobfeuerborn (1908)
IW1312-43	*Erinaceus europaeus*	34	28-40	birth	Hayssen et al. (1993)
IW1312-44	*Erinaceus europaeus*	48	48	14d after birth, eye opening	http://eol.org/pages/1178684/overview [2014-05-02]
IW1312-45	*Erinaceus europaeus*	76	42d after birth	weaning	http://genomics.senescence.info/species/entry.php?species=Erinaceus_europaeus [2014-05-02]
IW1445	*Erinaceus ("concolor") roumanicus*				
IW1445-01	*Erinaceus roumanicus*	15	15	third level	Štĕrba (1977a)
IW1445-02	*Erinaceus roumanicus*	17	17	forth level	Štĕrba (1977a)
IW1445-03	*Erinaceus roumanicus*	20	20	fifth level	Štĕrba (1977a)
IW1445-04	*Erinaceus roumanicus*	22	22	sixth level	Štĕrba (1977a)
IW1445-05	*Erinaceus roumanicus*	25	25	seventh level	Štĕrba (1977a)
IW1445-06	*Erinaceus roumanicus*	29	29	eigth level	Štĕrba (1977a)
IW1445-07	*Erinaceus roumanicus*	32	32	ninth level	Štĕrba (1977a)
IW1445-08	*Erinaceus roumanicus*	35	35	birth	Štĕrba (1977a)
IW1445-09	*Erinaceus roumanicus*	43	43	Fig. 2C12	Vogel (1972)
IW1445-10	*Erinaceus roumanicus*	49	49	14d after birth [*Erinaceus europaeus*]	http://eol.org/pages/1178684/overview [2014-05-02]
IW1445-11	*Erinaceus roumanicus*	77	42d after birth	Weaning [*Erinaceus europaeus*]	http://genomics.senescence.info/species/entry.php?species=Erinaceus_europaeus [2014-05-02]
IW1282	*Erythrocebus patas*		d		
IW1282-01	*Erythrocebus patas*	30	30	12	Binkerd et al. (1984)
IW1282-02	*Erythrocebus patas*	34	34	14	Binkerd et al. (1984)
IW1282-03	*Erythrocebus patas*	37	34-40	15	Binkerd et al. (1984)
IW1282-04	*Erythrocebus patas*	38.5	30+/-5.37	16	Binkerd et al. (1984)
IW1282-05	*Erythrocebus patas*	40	40	17	Binkerd et al. (1984)
IW1282-06	*Erythrocebus patas*	42.5	30+/-5	19	Binkerd et al. (1984)
IW1282-07	*Erythrocebus patas*	45	45	21	Binkerd et al. (1984)
IW1282-08	*Erythrocebus patas*	50	50	23	Binkerd et al. (1984)
IW1282-09	*Erythrocebus patas*	170	170	Birth (stimated eye opening)	http://animaldiversity.ummz.umich.edu/accounts/Erythrocebus_patas/ (2014-02-11)
IW1282-10	*Erythrocebus patas*	332	162d after birth	weaning	http://genomics.senescence.info/species/entry.php?species=Erythrocebus_patas [2014-05-02]
IW1329	*Felis catus*				
IW1329-01	*Felis catus forma domestica*	3.5	2.5-4.5	stage/figure 1	Knospe (2002) (information added from Rüsse and Sinowatz 1991 + Evans and Sack 1973)
IW1329-02	*Felis catus forma domestica*	5.25	4.5-6	stage/figure 2	Knospe (2002) (information added from Rüsse and Sinowatz 1991 + Evans and Sack 1973)
IW1329-03	*Felis catus forma domestica*	7.25	6-8.5	stage/figure 3	Knospe (2002) (information added from Rüsse and Sinowatz 1991 + Evans and Sack 1973)
IW1329-04	*Felis catus forma domestica*	9.5	8.5-10.5	stage/figure 4	Knospe (2002) (information added from Rüsse and Sinowatz 1991 + Evans and

					Sack 1973)
IW1329-05	*Felis catus forma domestica*	11.25	10.5-12	stage/figure 5	Knospe (2002) (information added from Rüsse and Sinowatz 1991 + Evans and Sack 1973)
IW1329-06	*Felis catus forma domestica*	12.5	12-13	stage/figure 6	Knospe (2002) (information added from Rüsse and Sinowatz 1991 + Evans and Sack 1973)
IW1329-07	*Felis catus forma domestica*	13.5	13-14	stage/figure 7	Knospe (2002) (information added from Rüsse and Sinowatz 1991 + Evans and Sack 1973)
IW1329-08	*Felis catus forma domestica*	14.5	14-15	stage/figure 8	Knospe (2002) (information added from Rüsse and Sinowatz 1991 + Evans and Sack 1973)
IW1329-09	*Felis catus forma domestica*	16	15-17	stage/figure 9	Knospe (2002) (information added from Rüsse and Sinowatz 1991 + Evans and Sack 1973)
IW1329-10	*Felis catus forma domestica*	17	16-18	stage/figure 10	Knospe (2002) (information added from Rüsse and Sinowatz 1991 + Evans and Sack 1973)
IW1329-11	*Felis catus forma domestica*	17.5	17-18	stage/figure 11	Knospe (2002) (information added from Rüsse and Sinowatz 1991 + Evans and Sack 1973)
IW1329-12	*Felis catus forma domestica*	18.5	18-19	stage/figure 12	Knospe (2002) (information added from Rüsse and Sinowatz 1991 + Evans and Sack 1973)
IW1329-13	*Felis catus forma domestica*	20	19-21	stage/figure 13	Knospe (2002) (information added from Rüsse and Sinowatz 1991 + Evans and Sack 1973)
IW1329-14	*Felis catus forma domestica*	22	21-23	stage/figure 14	Knospe (2002) (information added from Rüsse and Sinowatz 1991 + Evans and Sack 1973)
IW1329-15	*Felis catus forma domestica*	24	23-25	stage/figure 15	Knospe (2002) (information added from Rüsse and Sinowatz 1991 + Evans and Sack 1973)
IW1329-16	*Felis catus forma domestica*	26.5	25-28	stage/figure 16	Knospe (2002) (information added from Rüsse and Sinowatz 1991 + Evans and Sack 1973)
IW1329-17	*Felis catus forma domestica*	30	28-32	stage/figure 17	Knospe (2002) (information added from Rüsse and Sinowatz 1991 + Evans and Sack 1973)
IW1329-18	*Felis catus forma domestica*	35	32-38	stage/figure 18	Knospe (2002) (information added from Rüsse and Sinowatz 1991 + Evans and Sack 1973)
IW1329-19	*Felis catus forma domestica*	41	38-44	stage/figure 19	Knospe (2002) (information added from Rüsse and Sinowatz 1991 + Evans and Sack 1973)
IW1329-20	*Felis catus forma domestica*	46	44-48	stage/figure 20	Knospe (2002) (information added from Rüsse and Sinowatz 1991 + Evans and Sack 1973)
IW1329-21	*Felis catus forma domestica*	54	48-60	stage/figure 21	Knospe (2002) (information added from Rüsse and Sinowatz 1991 + Evans and Sack 1973)
IW1329-22	*Felis catus forma domestica*	62	58-66 (birth)	stage/figure 22	Knospe (2002) (information added from Rüsse and Sinowatz 1991 + Evans and Sack 1973)
IW1329-23	*Felis catus forma domestica*	81.5	81.5	19-20d after birth	http://www.brainmuseum.org/specimens/carnivora/cat/index.html [2014-05-02]
IW1329-24	*Felis catus forma*	118	56d after birth	weaning	http://genomics.senescence.in

	domestica				fo/species/entry.php?species=Felis_catus [2014-05-02]
IW1330	*Galago senegalensis*				
IW1330-01	*Galago senegalensis*	26.5	25-28	primitive streak stage	Butler (1972); summarized by Butler and Juurlink (1987)
IW1330-02	*Galago senegalensis*	33.5	x	Carnegie Stage 11	Butler (1972); summarized by Butler and Juurlink (1987)
IW1330-03	*Galago senegalensis*	40.5	x	Carnegie Stage 18	Butler (1972); summarized by Butler and Juurlink (1987)
IW1330-04	*Galago senegalensis*	47.5	x	Carnegie Stage 19/20	Butler (1972); summarized by Butler and Juurlink (1987)
IW1330-05	*Galago senegalensis*	54.5	x	Carnegie Stage 22	Butler (1972); summarized by Butler and Juurlink (1987)
IW1330-06	*Galago senegalensis*	61.5	61-62	Carnegie Stage 23	Butler (1972); summarized by Butler and Juurlink (1987)
IW1330-07	*Galago senegalensis*	133.5	122-145	Birth / eye opening	Hayssen et al. (1993) /http://www.brainmuseum.org/specimens/primates/lessbushbaby/index.html
IW1330-08	*Galago senegalensis*	222.5	89 after birth	weaning	http://genomics.senescence.info/species/entry.php?species=Galago_senegalensis [2014-05-02]
IW1303	*Hipposideros armiger*				
IW1303-01	*Hipposideros armiger*	x	x	stage 10	Wang et al. (2010)
IW1303-02	*Hipposideros armiger*	x	x	stage 11	Wang et al. (2010)
IW1303-03	*Hipposideros armiger*	x	x	stage 12	Wang et al. (2010)
IW1303-04	*Hipposideros armiger*	x	x	stage 13	Wang et al. (2010)
IW1303-05	*Hipposideros armiger*	x	x	stage 14	Wang et al. (2010)
IW1303-06	*Hipposideros armiger*	x	x	stage 15	Wang et al. (2010)
IW1303-07	*Hipposideros armiger*	x	x	stage 16	Wang et al. (2010)
IW1303-08	*Hipposideros armiger*	x	x	stage 17	Wang et al. (2010)
IW1303-09	*Hipposideros armiger*	x	x	stage 18	Wang et al. (2010)
IW1303-10	*Hipposideros armiger*	x	x	stage 19	Wang et al. (2010)
IW1303-11	*Hipposideros armiger*	x	x	stage 20	Wang et al. (2010)
IW1303-12	*Hipposideros armiger*	x	x	stage 21	Wang et al. (2010)
IW1303-13	*Hipposideros armiger*	x	x	stage 22	Wang et al. (2010)
IW1303-14	*Hipposideros armiger*	x	x	stage 23	Wang et al. (2010)
IW1303-15	*Hipposideros armiger*	x	x	stage 24 (fetal stage)	Wang et al. (2010)
IW1303-16	*Hipposideros armiger*	x	x	Birth	-
IW1304	*Hipposideros pratti*				
IW1304-01	*Hipposideros pratti*	x	x	stage 10	Wang et al. (2010)
IW1304-02	*Hipposideros pratti*	x	x	stage 11	Wang et al. (2010)
IW1304-03	*Hipposideros pratti*	x	x	stage 12	Wang et al. (2010)
IW1304-04	*Hipposideros pratti*	x	x	stage 13	Wang et al. (2010)
IW1304-05	*Hipposideros pratti*	x	x	stage 14	Wang et al. (2010)
IW1304-06	*Hipposideros pratti*	x	x	stage 15	Wang et al. (2010)
IW1304-07	*Hipposideros pratti*	x	x	stage 16	Wang et al. (2010)
IW1304-08	*Hipposideros pratti*	x	x	stage 18	Wang et al. (2010)
IW1304-09	*Hipposideros pratti*	x	x	stage 19	Wang et al. (2010)
IW1304-10	*Hipposideros pratti*	x	x	stage 20	Wang et al. (2010)
IW1304-11	*Hipposideros pratti*	x	x	stage 22	Wang et al. (2010)
IW1304-12	*Hipposideros pratti*	x	x	stage 24 (fetal stage)	Wang et al. (2010)
IW1304-13	*Hipposideros pratti*	x	x	birth	-
IW1273	*Homo sapiens (1)*		d		
IW1273-01	*Homo sapiens (1)*	1	1	1	O'Rahilly and Müller (1987)
IW1273-02	*Homo sapiens (1)*	2.25	1.5-3	2	O'Rahilly and Müller (1987)
IW1273-03	*Homo sapiens (1)*	4	4	3	O'Rahilly and Müller (1987)

IW1273-04	*Homo sapiens (1)*	5.5	5-6	4	O'Rahilly and Müller (1987)
IW1273-05	*Homo sapiens (1)*	9.5	7-12	5	O'Rahilly and Müller (1987)
IW1273-06	*Homo sapiens (1)*	13	13	6	O'Rahilly and Müller (1987)
IW1273-07	*Homo sapiens (1)*	16	16	7	O'Rahilly and Müller (1987)
IW1273-08	*Homo sapiens (1)*	18	17-19	8	O'Rahilly and Müller (1987)
IW1273-09	*Homo sapiens (1)*	20	19-21	9	O'Rahilly and Müller (1987)
IW1273-10	*Homo sapiens (1)*	22	21-23	10	O'Rahilly and Müller (1987)
IW1273-11	*Homo sapiens (1)*	24	23-25	11	O'Rahilly and Müller (1987)
IW1273-12	*Homo sapiens (1)*	26	25-27	12	O'Rahilly and Müller (1987)
IW1273-13	*Homo sapiens (1)*	28	28	13	O'Rahilly and Müller (1987)
IW1273-14	*Homo sapiens (1)*	32	32	14	O'Rahilly and Müller (1987)
IW1273-15	*Homo sapiens (1)*	33	33	15	O'Rahilly and Müller (1987)
IW1273-16	*Homo sapiens (1)*	37	37	16	O'Rahilly and Müller (1987)
IW1273-17	*Homo sapiens (1)*	41	41	17	O'Rahilly and Müller (1987)
IW1273-18	*Homo sapiens (1)*	44	44	18	O'Rahilly and Müller (1987)
IW1273-19	*Homo sapiens (1)*	47.5	47-48	19	O'Rahilly and Müller (1987)
IW1273-20	*Homo sapiens (1)*	50.5	50-51	20	O'Rahilly and Müller (1987)
IW1273-21	*Homo sapiens (1)*	52	52	21	O'Rahilly and Müller (1987)
IW1273-22	*Homo sapiens (1)*	54	54	22	O'Rahilly and Müller (1987)
IW1273-23	*Homo sapiens (1)*	56.5	56-57	23	O'Rahilly and Müller (1987)
IW1273-24	*Homo sapiens (1)*	70	10. week	x	http://www.baby-lexikon.com (11.02.2011)
IW1273-25	*Homo sapiens (1)*	91	13. week	x	http://www.baby-lexikon.com (11.02.2011)
IW1273-26	*Homo sapiens (1)*	98	14. week	x	http://www.baby-lexikon.com (11.02.2011)
IW1273-27	*Homo sapiens (1)*	161	23. week	X	http://www.baby-lexikon.com (11.02.2011)
IW1273-28	*Homo sapiens (1)*	205	205	eye lid opens at day 205	Müller (1972)
IW1273-29	*Homo sapiens (1)*	266	40. week	x	http://www.baby-lexikon.com (11.02.2011)
IW1273-30	*Homo sapiens (1)*	905	639 after birth	weaning	http://genomics.senescence.info/species/entry.php?species=Homo_sapiens [2014-05-02]
IW1274	*Homo sapiens (2)*			images	
IW1274-01	*Homo sapiens (2)*	4	x	I	Keibel and Elze (1908)
IW1274-02	*Homo sapiens (2)*	8	x	II	Keibel and Elze (1908)
IW1274-03	*Homo sapiens (2)*	12	10-14	III	Keibel and Elze (1908)
IW1274-04	*Homo sapiens (2)*	13.5	13-14	IV	Keibel and Elze (1908)
IW1274-05	*Homo sapiens (2)*	14.5	x	V	Keibel and Elze (1908)
IW1274-06	*Homo sapiens (2)*	15.5	x	VI	Keibel and Elze (1908)
IW1274-07	*Homo sapiens (2)*	16.5	ca. 3 weeks	VII	Keibel and Elze (1908)
IW1274-08	*Homo sapiens (2)*	17.5	4 weeks	VIII	Keibel and Elze (1908)
IW1274-09	*Homo sapiens (2)*	18.5	x	IX	Keibel and Elze (1908)
IW1274-10	*Homo sapiens (2)*	19.5	x	X	Keibel and Elze (1908)
IW1274-11	*Homo sapiens (2)*	21	21	XI	Keibel and Elze (1908)
IW1274-12	*Homo sapiens (2)*	24.75	x	XII	Keibel and Elze (1908)
IW1274-13	*Homo sapiens (2)*	28.5	27-28	XIII	Keibel and Elze (1908)
IW1274-14	*Homo sapiens (2)*	29.2	x	XIV	Keibel and Elze (1908)
IW1274-15	*Homo sapiens (2)*	29.9	x	XV	Keibel and Elze (1908)
IW1274-16	*Homo sapiens (2)*	30.6	ca. 4 weeks	XVI	Keibel and Elze (1908)
IW1274-17	*Homo sapiens (2)*	31.3	x	XVII	Keibel and Elze (1908)
IW1274-18	*Homo sapiens (2)*	32	32-33	XVIII	Keibel and Elze (1908)
IW1274-19	*Homo sapiens (2)*	32.5	31	XIX	Keibel and Elze (1908)
IW1274-20	*Homo sapiens (2)*	40.3	x	XX	Keibel and Elze (1908)
IW1274-21	*Homo sapiens (2)*	48.2	x	XI	Keibel and Elze (1908)
IW1274-22	*Homo sapiens (2)*	56	56	XXII	Keibel and Elze (1908)
IW1274-23	*Homo sapiens (2)*	58	x	XXIII	Keibel and Elze (1908)
IW1274-24	*Homo sapiens (2)*	60	60	XXIV	Keibel and Elze (1908)
IW1274-25	*Homo sapiens (2)*	163	x	XV	Keibel and Elze (1908)
IW1274-26	*Homo sapiens (2)*	205	205	Eye opening	Müller (1972)
IW1274-27	*Homo sapiens (2)*	266	266	birth	http://www.baby-lexikon.com (11.02.2011)
IW1274-28	*Homo sapiens (2)*	905	639 after birth	weaning	http://genomics.senescence.info/species/entry.php?species=Homo_sapiens [2014-05-02]
IW1252	*Loxodonta africana*	d (1 month = 30.42d)			
IW1252-01	*Loxodonta africana*	58	58d / 60d	Figure 1A / Figure 3a	Geath et al. (1999) / Hildebrandt et al. (2007)
IW1252-02	*Loxodonta africana*	69.5	less 1 month (1g) (referred age not applied to this study)	Figure 1A	Raubenheimer (2000)
IW1252-03	*Loxodonta africana*	81	81d	Figure 3b	Hildebrandt et al. (2007)
IW1252-04	*Loxodonta africana*	92	92d	Figure 3c	Hildebrandt et al. (2007)

IW1252-05	*Loxodonta africana*	97	97d	Figure 3d	Hildebrandt et al. (2007)
IW1252-06	*Loxodonta africana*	100	100d	Figure 3e	Hildebrandt et al. (2007)
IW1252-07	*Loxodonta africana*	103	103d	Figure 3f	Hildebrandt et al. (2007)
IW1252-08	*Loxodonta africana*	136.9	4.5 months (twice mentioned, preferred herein) / 5.3 months (inconstequently used in reference)	Fig 2A, S15-16 / Fig. S17	Allen (2006)
IW1252-09	*Loxodonta africana*	150	150d	Figure 3g	Hildebrandt et al. (2007)
IW1252-10	*Loxodonta africana*	166	166d	Figure 1B	Geath et al. (1999)
IW1252-11	*Loxodonta africana*	167	167d	Figure 3h	Hildebrandt et al. (2007)
IW1252-12	*Loxodonta africana*	626.652	20.6 months	Fig. S24, birth (estimated eye opening)	Allen (2006)
IW1252-13	*Loxodonta africana*	1697.652	1071 days after birth	weaning	http://genomics.senescence.info/species/entry.php?species=Loxodonta_africana [2014-05-02]
IW1336	*Macaca mulatta* (1)				
IW1336-01	*Macaca mulatta* (1)	13.5	12-15	stage 6	Hendrickx and Sawyer (1975) = cited in Butler and Juurlink (1987)
IW1336-02	*Macaca mulatta* (1)	20.5	20-21	stage 9	Hendrickx and Sawyer (1975) = cited in Butler and Juurlink (1987)
IW1336-03	*Macaca mulatta* (1)	22	21-23	stage 10	Hendrickx and Sawyer (1975) = cited in Butler and Juurlink (1987)
IW1336-04	*Macaca mulatta* (1)	25	24-26	stage 11	Hendrickx and Sawyer (1975) = cited in Butler and Juurlink (1987)
IW1336-05	*Macaca mulatta* (1)	27.5	27-28	stage 12	Hendrickx and Sawyer (1975) = cited in Butler and Juurlink (1987)
IW1336-06	*Macaca mulatta* (1)	29	28-30	stage 13	Hendrickx and Sawyer (1975) = cited in Butler and Juurlink (1987)
IW1336-07	*Macaca mulatta* (1)	31	30-32	stage 14	Hendrickx and Sawyer (1975) = cited in Butler and Juurlink (1987)
IW1336-08	*Macaca mulatta* (1)	31.5	30-33	stage 15	Hendrickx and Sawyer (1975) = cited in Butler and Juurlink (1987)
IW1336-09	*Macaca mulatta* (1)	33	32-34	stage 16	Hendrickx and Sawyer (1975) = cited in Butler and Juurlink (1987)
IW1336-10	*Macaca mulatta* (1)	35	34-36	stage 17	Hendrickx and Sawyer (1975) = cited in Butler and Juurlink (1987)
IW1336-11	*Macaca mulatta* (1)	36.5	35-38	stage 18	Hendrickx and Sawyer (1975) = cited in Butler and Juurlink (1987)
IW1336-12	*Macaca mulatta* (1)	39	36-42	stage 19	Hendrickx and Sawyer (1975) = cited in Butler and Juurlink (1987)
IW1336-13	*Macaca mulatta* (1)	40	38-42	stage 20	Hendrickx and Sawyer (1975) = cited in Butler and Juurlink (1987)
IW1336-14	*Macaca mulatta* (1)	42	40-44	stage 21	Hendrickx and Sawyer (1975) = cited in Butler and Juurlink (1987)
IW1336-15	*Macaca mulatta* (1)	45	45	stage 22	Hendrickx and Sawyer (1975) = cited in Butler and Juurlink (1987)
IW1336-16	*Macaca mulatta* (1)	47	47	stage 23	Hendrickx and Sawyer (1975) = cited in Butler and Juurlink (1987)
IW1336-17	*Macaca mulatta* (1)	137.5	135-140	Eye opening	Müller (1972)
IW1336-18	*Macaca mulatta* (1)	164	164	birth	http://pin.primate.wisc.edu/factsheets/entry/rhesus_macaque [2014-01-21]
IW1336-19	*Macaca mulatta* (1)	456	292d after birth	weaning	http://genomics.senescence.info/species/entry.php?species=Macaca_mulatta [2014-05-02]
IW1443	*Macaca mulatta* (2)				
IW1443-01	*Macaca mulatta* (2)	18	18	primitive streak/groove	Heuser and Hartmann (1941)
IW1443-02	*Macaca mulatta* (2)	21	21	Fig. 214	Heuser and Hartmann (1941)

IW1443-03	*Macaca mulatta* (2)	21.375	21	Fig. 215	Heuser and Hartmann (1941)
IW1443-04	*Macaca mulatta* (2)	21.75	21.75	Fig. 216	Heuser and Hartmann (1941)
IW1443-05	*Macaca mulatta* (2)	23.5	23-24	Fig. 217	Heuser and Hartmann (1941)
IW1443-06	*Macaca mulatta* (2)	24	24	Fig. 218	Heuser and Hartmann (1941)
IW1443-07	*Macaca mulatta* (2)	26	26	Fig. 219	Heuser and Hartmann (1941)
IW1443-08	*Macaca mulatta* (2)	26.5	26.5	Fig. 220, Fig. 228A	Heuser and Hartmann (1941)
IW1443-09	*Macaca mulatta* (2)	27	27	Fig. 221, Fig. 228B	Heuser and Hartmann (1941)
IW1443-10	*Macaca mulatta* (2)	29	29	Fig. 222, Fig. 228C	Heuser and Hartmann (1941)
IW1443-11	*Macaca mulatta* (2)	31	31	Fig. 223, Fig. 228D	Heuser and Hartmann (1941)
IW1443-12	*Macaca mulatta* (2)	32	32	Fig. 224, Fig. 228E	Heuser and Hartmann (1941)
IW1443-13	*Macaca mulatta* (2)	34	34	Fig. 225, Fig. 228F	Heuser and Hartmann (1941)
IW1443-14	*Macaca mulatta* (2)	36	36	Fig. 226, Fig. 228G	Heuser and Hartmann (1941)
IW1443-15	*Macaca mulatta* (2)	36.5	36 (more advanced)	Fig. 227	Heuser and Hartmann (1941)
IW1443-16	*Macaca mulatta* (2)	44	44	Fig. 228H	Heuser and Hartmann (1941)
IW1443-17	*Macaca mulatta* (2)	53	53	Fig. 228I	Heuser and Hartmann (1941)
IW1443-18	*Macaca mulatta* (2)	57	57	Fig. 228J	Heuser and Hartmann (1941)
IW1443-19	*Macaca mulatta* (2)	137.5	135-140	Eye opening	Müller (1972)
IW1443-20	*Macaca mulatta* (2)	164	164	birth	http://pin.primate.wisc.edu/factsheets/entry/rhesus_macaque (2014-01-21)
IW1336-21	*Macaca mulatta* (2)	456	292d after birth	weaning	http://genomics.senescence.info/species/entry.php?species=Macaca_mulatta [2014-05-02]
IW1287	*Manis javanica*			mixture: specimen/stage	
IW1287-01	*Manis javanica*	x	x	9	van Oordt (1921) (this specimen was not used by Huisman 1933)
IW1287-02	*Manis javanica*	x	x	1 / 10, 33-35	Huisman (1933) / van Oordt (1921)
IW1287-03	*Manis javanica*	x	x	2 / 11, 12, 36-43, 62	Huisman (1933) / van Oordt (1921)
IW1287-04	*Manis javanica*	x	x	3 / 13, 44, 63	Huisman (1933) / van Oordt (1921)
IW1287-05	*Manis javanica*	x	x	4 / 14, 45-50, 64	Huisman (1933) / van Oordt (1921)
IW1287-06	*Manis javanica*	x	x	5 / 15, 51-55, 65	Huisman (1933) / van Oordt (1921)
IW1287-07	*Manis javanica*	x	x	6 / 16, 56	Huisman (1933) / van Oordt (1921)
IW1287-08	*Manis javanica*	x	x	7 / 17, 18, 57, 66	Huisman (1933) / van Oordt (1921)
IW1287-09	*Manis javanica*	x	x	8 / 19, 58-61, 67	Huisman (1933) / van Oordt (1921)
IW1287-10	*Manis javanica*	x	x	9 = Figure I	Huisman (1933)
IW1287-11	*Manis javanica*	x	x	10	Huisman (1933)
IW1287-12	*Manis javanica*	x	x	41	Weber (1892)
IW1287-13	*Manis javanica*	x	x	53	Weber (1892)
IW1287-14	*Manis javanica*	x	x	11 = Figure II	Huisman (1933)
IW1287-15	*Manis javanica*	x	x	12	Huisman (1933)
IW1287-16	*Manis javanica*	x	x	13	Huisman (1933)
IW1287-17	*Manis javanica*	x	x	14	Huisman (1933)
IW1287-18	*Manis javanica*	x	x	15	Huisman (1933)
IW1287-19	*Manis javanica*	x	x	16 = Figure III	Huisman (1933)
IW1287-20	*Manis javanica*	x	x	17	Huisman (1933)
IW1287-21	*Manis javanica*	x	x	18	Huisman (1933)
IW1287-22	*Manis javanica*	x	x	19 = Figure IV	Huisman (1933)
IW1287-23	*Manis javanica*	x	x	20 = Figures V, VI	Huisman (1933)
IW1287-24	*Manis javanica*	x	x	21 (+/- Fig. VII)/ 42	Huisman (1933) / Hubrecht (1894)
IW1287-25	*Manis javanica*	x	x	22 (Fig. VII = between stage 21 and 22) / 49+71	Huisman (1933) / Weber (1892)
IW1287-26	*Manis javanica*	130	about 130	birth	http://animaldiversity.ummz.umich.edu/accounts/Manis_javanica/ (2014-01-21)
IW1287-27	*Manis javanica*	140	10d after birth	Eye opening	http://www.wildlifeatrisk.org/UserFiles/File/species%20at%20risk-jpangolin.pdf (2014-04-23)
IW1287-28	*Manis javanica*	221.25	3 months after birth	weaning	http://animaldiversity.ummz.umich.edu/accounts/Manis_javanica/ [2014-05-02]
IW1454	*Meriones unguiculatus*				
IW1454-01	*Meriones unguiculatus*	12	12	Figure 1, 14	Bagwell and Leavitt (1974); summarized by Štěrba

					(1977b)
IW1454-02	*Meriones unguiculatus*	13	13	Figure 2, 15 (for figure number see text description, incorrectly labeled in figure legend)	Bagwell and Leavitt (1974); summarized by Štěrba (1977b)
IW1454-03	*Meriones unguiculatus*	14	14	Figure 3, 16 (for figure number see text description, incorrectly labeled in figure legend)	Bagwell and Leavitt (1974); summarized by Štěrba (1977b)
IW1454-04	*Meriones unguiculatus*	15	15	Figure 4, 17	Bagwell and Leavitt (1974); summarized by Štěrba (1977b)
IW1454-05	*Meriones unguiculatus*	16	16	Figure 5	Bagwell and Leavitt (1974); summarized by Štěrba (1977b)
IW1454-06	*Meriones unguiculatus*	17	17	Figure 6	Bagwell and Leavitt (1974); summarized by Štěrba (1977b)
IW1454-07	*Meriones unguiculatus*	18	18	Figure 7	Bagwell and Leavitt (1974); summarized by Štěrba (1977b)
IW1454-08	*Meriones unguiculatus*	19	19	Figure 8	Bagwell and Leavitt (1974); summarized by Štěrba (1977b)
IW1454-09	*Meriones unguiculatus*	20	20	Figure 9	Bagwell and Leavitt (1974); summarized by Štěrba (1977b)
IW1454-10	*Meriones unguiculatus*	21	21	Figure 10	Bagwell and Leavitt (1974); summarized by Štěrba (1977b)
IW1454-11	*Meriones unguiculatus*	22	22	Figure 11	Bagwell and Leavitt (1974); summarized by Štěrba (1977b)
IW1454-12	*Meriones unguiculatus*	23	23	Figure 12	Bagwell and Leavitt (1974); summarized by Štěrba (1977b)
IW1454-13	*Meriones unguiculatus*	24	24	Figure 13	Bagwell and Leavitt (1974); summarized by Štěrba (1977b)
IW1454-14	*Meriones unguiculatus*	25	25	birth	Bagwell and Leavitt (1974); summarized by Štěrba (1977b)
IW1454-15	*Meriones unguiculatus*	43	16-20 after birth	eye opens	Gulotta (1971)
IW1454-16	*Meriones unguiculatus*	49	24 after birth	weaning	http://genomics.senescence.info/species/entry.php?species=Meriones_unguiculatus [2014-05-02]
IW1275	*Mesocricetus auratus*				
IW1275-01	*Mesocricetus auratus*	0.33	8h (fertilisation)	text description	Boyer (1953, same as: 1968)
IW1275-02	*Mesocricetus auratus*	7	7 (primitive streak)	text description	Boyer (1953, same as: 1968)
IW1275-03	*Mesocricetus auratus*	7.5	7.5	text description	Boyer (1953, same as: 1968)
IW1275-04	*Mesocricetus auratus*	7.75	7.75	figure 1	Boyer (1953, same as: 1968)
IW1275-05	*Mesocricetus auratus*	8	8	figure 2	Boyer (1953, same as: 1968)
IW1275-06	*Mesocricetus auratus*	8.25	8.25	text description	Boyer (1953, same as: 1968)
IW1275-07	*Mesocricetus auratus*	8.5	8.5	figure 3	Boyer (1953, same as: 1968)
IW1275-08	*Mesocricetus auratus*	8.75	8.75	figure 4	Boyer (1953, same as: 1968)
IW1275-09	*Mesocricetus auratus*	9	9	figure 5	Boyer (1953, same as: 1968)
IW1275-10	*Mesocricetus auratus*	9.25	9.25	figure 6	Boyer (1953, same as: 1968)
IW1275-11	*Mesocricetus auratus*	9.5	9.5	figure 7	Boyer (1953, same as: 1968)
IW1275-12	*Mesocricetus auratus*	9.75	9.75	figure 8	Boyer (1953, same as: 1968)
IW1275-13	*Mesocricetus auratus*	10	10	figure 9	Boyer (1953, same as: 1968)
IW1275-14	*Mesocricetus*	10.5	10.5	figure 10	Boyer (1953, same as: 1968)

	auratus				
IW1275-15	*Mesocricetus auratus*	11	11	figure 11	Boyer (1953, same as: 1968)
IW1275-16	*Mesocricetus auratus*	11.5	11.5	figure 12	Boyer (1953, same as: 1968)
IW1275-17	*Mesocricetus auratus*	12	12	figure 13	Boyer (1953, same as: 1968)
IW1275-18	*Mesocricetus auratus*	12.5	12.5	figure 14	Boyer (1953, same as: 1968)
IW1275-19	*Mesocricetus auratus*	13	13	figure 15	Boyer (1953, same as: 1968)
IW1275-20	*Mesocricetus auratus*	15	15	figure 16	Boyer (1953, same as: 1968)
IW1275-21	*Mesocricetus auratus*	16	16	birth	Boyer (1953, same as: 1968)
IW1275-22	*Mesocricetus auratus*	28	28	eye opens	Müller (1972)
IW1275-23	*Mesocricetus auratus*	36	20d after birth	weaning	http://genomics.senescence.info/species/entry.php?species=Mesocricetus_auratus [2014-05-02]
IW1313	*Miniopterus natalensis*				
IW1313-01	*Miniopterus natalensis*	x	x	stage CS (Cretekos Stage) 12	Hockman et al. (2009)
IW1313-02	*Miniopterus natalensis*	x	x	stage CS (Cretekos Stage) 13	Hockman et al. (2009)
IW1313-03	*Miniopterus natalensis*	x	x	stage CS (Cretekos Stage) 13L	Hockman et al. (2009)
IW1313-04	*Miniopterus natalensis*	x	x	stage CS (Cretekos Stage) 14E	Hockman et al. (2009)
IW1313-05	*Miniopterus natalensis*	x	x	stage CS (Cretekos Stage) 14	Hockman et al. (2009)
IW1313-06	*Miniopterus natalensis*	x	x	stage CS (Cretekos Stage) 14L	Hockman et al. (2009)
IW1313-07	*Miniopterus natalensis*	x	x	stage CS (Cretekos Stage) 15VE	Hockman et al. (2009)
IW1313-08	*Miniopterus natalensis*	x	x	stage CS (Cretekos Stage) 15E	Hockman et al. (2009)
IW1313-09	*Miniopterus natalensis*	x	x	stage CS (Cretekos Stage) 15	Hockman et al. (2009)
IW1313-10	*Miniopterus natalensis*	x	x	stage CS (Cretekos Stage) 16E	Hockman et al. (2009)
IW1313-11	*Miniopterus natalensis*	x	x	stage CS (Cretekos Stage) 16	Hockman et al. (2009)
IW1313-12	*Miniopterus natalensis*	x	x	stage CS (Cretekos Stage) 17	Hockman et al. (2009)
IW1313-13	*Miniopterus natalensis*	x	x	stage CS (Cretekos Stage) 18	Hockman et al. (2009)
IW1313-14	*Miniopterus natalensis*	x	x	stage CS (Cretekos Stage) 19	Hockman et al. (2009)
IW1313-15	*Miniopterus natalensis*	x	x	stage CS (Cretekos Stage) 20	Hockman et al. (2009)
IW1313-16	*Miniopterus natalensis*	x	x	stage CS (Cretekos Stage) 21	Hockman et al. (2009)
IW1313-17	*Miniopterus natalensis*	126.88	126.88 (average)	birth	http://eol.org/pages/4437078/overview (2014-02-10)
IW1305	*Miniopterus schreibersii fuliginosus*				
IW1305-01	*Miniopterus schreibersii fuliginosus*	x	x	stage 10	Wang et al. (2010)
IW1305-02	*Miniopterus schreibersii fuliginosus*	x	x	stage 11	Wang et al. (2010)
IW1305-03	*Miniopterus schreibersii fuliginosus*	x	x	stage 12	Wang et al. (2010)
IW1305-04	*Miniopterus schreibersii fuliginosus*	x	x	stage 13	Wang et al. (2010)
IW1305-05	*Miniopterus schreibersii fuliginosus*	x	x	stage 14	Wang et al. (2010)
IW1305-06	*Miniopterus schreibersii*	x	x	stage 15	Wang et al. (2010)

	fuliginosus				
IW1305-07	*Miniopterus schreibersii fuliginosus*	x	x	stage 16	Wang et al. (2010)
IW1305-08	*Miniopterus schreibersii fuliginosus*	x	x	stage 17	Wang et al. (2010)
IW1305-09	*Miniopterus schreibersii fuliginosus*	x	x	stage 18	Wang et al. (2010)
IW1305-10	*Miniopterus schreibersii fuliginosus*	x	x	stage 19	Wang et al. (2010)
IW1305-11	*Miniopterus schreibersii fuliginosus*	x	x	stage 20	Wang et al. (2010)
IW1305-12	*Miniopterus schreibersii fuliginosus*	x	x	stage 21	Wang et al. (2010)
IW1305-13	*Miniopterus schreibersii fuliginosus*	x	x	stage 22	Wang et al. (2010)
IW1305-14	*Miniopterus schreibersii fuliginosus*	x	x	stage 23	Wang et al. (2010)
IW1305-15	*Miniopterus schreibersii fuliginosus*	x	x	stage 24 (fetal stage)	Wang et al. (2010)
IW1305-16	*Miniopterus schreibersii fuliginosus*	240	240	new born	http://animaldiversity.ummz.umich.edu/accounts/Miniopterus_schreibersii/ (2014-02-10)
IW1314	*Molossus rufus*				
IW1314-01	*Molossus rufus*	x	x	stage 13 (after Cretekos et al. 2005)	Nolte et al. (2009)
IW1314-02	*Molossus rufus*	x	x	stage 14	Nolte et al. (2009)
IW1314-03	*Molossus rufus*	x	x	stage 15	Nolte et al. (2009)
IW1314-04	*Molossus rufus*	x	x	stage 16	Nolte et al. (2009)
IW1314-05	*Molossus rufus*	x	x	stage 17	Nolte et al. (2009)
IW1314-06	*Molossus rufus*	x	x	stage 18	Nolte et al. (2009)
IW1314-07	*Molossus rufus*	x	x	stage 18L	Nolte et al. (2009)
IW1314-08	*Molossus rufus*	x	x	stage 20	Nolte et al. (2009)
IW1314-09	*Molossus rufus*	x	x	stage 21	Nolte et al. (2009)
IW1314-10	*Molossus rufus*	x	x	stage 22	Nolte et al. (2009)
IW1314-11	*Molossus rufus*	-	-	birth	-
IW1272	*Mus musculus*		d		
IW1272-01	*Mus musculus*	0.9	0.9	stage 1	Theiler (1989) / http://genex.hgu.mrc.ac.uk/Databases/Anatomy/Diagrams
IW1272-02	*Mus musculus*	1.125	1-1.25	stage 2	Theiler (1989) / http://genex.hgu.mrc.ac.uk/Databases/Anatomy/Diagrams
IW1272-03	*Mus musculus*	2.25	1-3.5	stage 3	Theiler (1989) / http://genex.hgu.mrc.ac.uk/Databases/Anatomy/Diagrams
IW1272-04	*Mus musculus*	3	2-4	stage 4	Theiler (1989) / http://genex.hgu.mrc.ac.uk/Databases/Anatomy/Diagrams
IW1272-05	*Mus musculus*	4.25	3-5.5	stage 5	Theiler (1989) / http://genex.hgu.mrc.ac.uk/Databases/Anatomy/Diagrams
IW1272-06	*Mus musculus*	4.75	4-5.5	stage 6	Theiler (1989) / http://genex.hgu.mrc.ac.uk/Databases/Anatomy/Diagrams
IW1272-07	*Mus musculus*	5.25	4.5-6	stage 7	Theiler (1989) / http://genex.hgu.mrc.ac.uk/Databases/Anatomy/Diagrams
IW1272-08	*Mus musculus*	5.75	5-6.5	stage 8	Theiler (1989) / http://genex.hgu.mrc.ac.uk/Databases/Anatomy/Diagrams
IW1272-09	*Mus musculus*	5.5	6-7	stage 9	Theiler (1989) / http://genex.hgu.mrc.ac.uk/Databases/Anatomy/Diagrams
IW1272-10	*Mus musculus*	7	6.5-7.5	stage 10	Theiler (1989) / http://genex.hgu.mrc.ac.uk/Databases/Anatomy/Diagrams
IW1272-11	*Mus musculus*	7.625	7.25-8	stage 11	Theiler (1989) /

					http://genex.hgu.mrc.ac.uk/Databases/Anatomy/Diagrams
IW1272-12	*Mus musculus*	8.125	7.5-8.75	stage 12	Theiler (1989) / http://genex.hgu.mrc.ac.uk/Databases/Anatomy/Diagrams
IW1272-13	*Mus musculus*	8.625	8-9.25	stage 13	Theiler (1989) / http://genex.hgu.mrc.ac.uk/Databases/Anatomy/Diagrams
IW1272-14	*Mus musculus*	9.125	8.5-9.75	stage 14	Theiler (1989) / http://genex.hgu.mrc.ac.uk/Databases/Anatomy/Diagrams
IW1272-15	*Mus musculus*	9.625	9-10.25	stage 15	Theiler (1989) / http://genex.hgu.mrc.ac.uk/Databases/Anatomy/Diagrams
IW1272-16	*Mus musculus*	10.125	9.5-10.75	stage 16	Theiler (1989) / http://genex.hgu.mrc.ac.uk/Databases/Anatomy/Diagrams
IW1272-17	*Mus musculus*	10.625	10-11.25	stage 17	Theiler (1989) / http://genex.hgu.mrc.ac.uk/Databases/Anatomy/Diagrams
IW1272-18	*Mus musculus*	10.875	10.5-11.25	stage 18	Theiler (1989) / http://genex.hgu.mrc.ac.uk/Databases/Anatomy/Diagrams
IW1272-19	*Mus musculus*	11.625	11-12.25	stage 19	Theiler (1989) / http://genex.hgu.mrc.ac.uk/Databases/Anatomy/Diagrams
IW1272-20	*Mus musculus*	12.25	11.5-13	stage 20	Theiler (1989) / http://genex.hgu.mrc.ac.uk/Databases/Anatomy/Diagrams
IW1272-21	*Mus musculus*	13.25	12.5-14	stage 21	Theiler (1989) / http://genex.hgu.mrc.ac.uk/Databases/Anatomy/Diagrams
IW1272-22	*Mus musculus*	14.25	13.5-15	stage 22	Theiler (1989) / http://genex.hgu.mrc.ac.uk/Databases/Anatomy/Diagrams
IW1272-23	*Mus musculus*	15	15	stage 23	Theiler (1989) / http://genex.hgu.mrc.ac.uk/Databases/Anatomy/Diagrams
IW1272-24	*Mus musculus*	16	16	stage 24	Theiler (1989) / http://genex.hgu.mrc.ac.uk/Databases/Anatomy/Diagrams
IW1272-25	*Mus musculus*	17	17	stage 25	Theiler (1989) / http://genex.hgu.mrc.ac.uk/Databases/Anatomy/Diagrams
IW1272-26	*Mus musculus*	18	18	stage 26	Theiler (1989) / http://genex.hgu.mrc.ac.uk/Databases/Anatomy/Diagrams
IW1272-27	*Mus musculus*	19.5	19-20 (birth)	stage 27	Theiler (1989) / http://genex.hgu.mrc.ac.uk/Databases/Anatomy/Diagrams
IW1272-28	*Mus musculus*	22	2-3d after birth	text in stage 27	Theiler (1989) / http://genex.hgu.mrc.ac.uk/Databases/Anatomy/Diagrams
IW1272-29	*Mus musculus*	32	12-13d after birth	stage 28	Theiler (1989) / http://genex.hgu.mrc.ac.uk/Databases/Anatomy/Diagrams [2014-05-02]
IW1272-30	*Mus musculus*	41.5	22d after birth	weaning	http://genomics.senescence.info/species/entry.php?species=Mus_musculus [2014-05-02]
IW1340	*Mustela putorius*		d		
IW1340-01	*Mustela putorius*	12	14 / less than 15 days / primitive streak earliest at 12d	day 14, figure 1 / figure 1 / figures 1-10	Gulamhusein and Beck (1981) / Yeates (1911) / Hamilton (1939)
IW1340-02	*Mustela putorius*	15	15 (4-8 somites) / 17 (5-7 somites)	day 15, figure 2 / figures 11-12	Gulamhusein and Beck (1981) / Hamilton (1939)
IW1340-03	*Mustela putorius*	16	16	day 16, figure 3	Gulamhusein and Beck (1981)
IW1340-04	*Mustela putorius*	17	17	day 17, figure 4	Gulamhusein and Beck (1981)
IW1340-05	*Mustela putorius*	18	18	day 18, figure 5	Gulamhusein and Beck (1981)
IW1340-06	*Mustela putorius*	19	19	day 19, figure 6 / figure 1	Gulamhusein and Beck (1981) / Radford (1911)
IW1340-07	*Mustela putorius*	20	20	day 20, figure 7	Gulamhusein and Beck (1981)
IW1340-08	*Mustela putorius*	21	21	day 21, figure 8	Gulamhusein and Beck

					(1981)
IW1340-09	*Mustela putorius*	22	22	day 22, figure 9	Gulamhusein and Beck (1981)
IW1340-10	*Mustela putorius*	23	23	day 23, figure 10 / figure 1	Gulamhusein and Beck (1981) / Good (1912)
IW1340-11	*Mustela putorius*	24	24	day 24, figure 11	Gulamhusein and Beck (1981)
IW1340-12	*Mustela putorius*	25	25	day 25, figure 12	Gulamhusein and Beck (1981)
IW1340-13	*Mustela putorius*	26	26	day 26, figure 13	Gulamhusein and Beck (1981)
IW1340-14	*Mustela putorius*	27	27	day 27, figure 13	Gulamhusein and Beck (1981)
IW1340-15	*Mustela putorius*	28	28	day 28	Gulamhusein and Beck (1981)
IW1340-16	*Mustela putorius*	29	29	day 29, figure 13	Gulamhusein and Beck (1981)
IW1340-17	*Mustela putorius*	30	30	day 30, figure 13	Gulamhusein and Beck (1981)
IW1340-18	*Mustela putorius*	31	31	day 31, figure 13	Gulamhusein and Beck (1981)
IW1340-19	*Mustela putorius*	32	32	day 32, figure 14	Gulamhusein and Beck (1981)
IW1340-20	*Mustela putorius*	35	35	day 35, figure 14	Gulamhusein and Beck (1981, 1983)
IW1340-21	*Mustela putorius*	37	37	day 37, figure 14	Gulamhusein and Beck (1981)
IW1340-22	*Mustela putorius*	38	38	day 38, figure 15	Gulamhusein and Beck (1981)
IW1340-23	*Mustela putorius*	40	40	day 40, figure 15	Gulamhusein and Beck (1981)
IW1340-24	*Mustela putorius*	42	42	day 42, figure 15, birth	Gulamhusein and Beck (1981)
IW1340-25	*Mustela putorius*	76	34d after birth	Eye opening	http://wildpro.twycrosszoo.org/S/0MCarnivor/Mustelidae/mustela/Mustela_putorius_furo/07Mustela_putorius_furoLifePhys.htm [2014-04-23]
IW1340-26	*Mustela putorius*	105	63d after birth	weaning	http://genomics.senescence.info/species/entry.php?species=Mustela_putorius [2014-05-02]
IW1446	*Myodes glareolus ("Clethrionomys")*		d		
IW1446-01	*Myodes glareolus*	9.5	8.5-10.5	Figure 6, third level	Štěrba (1976)
IW1446-02	*Myodes glareolus*	11.75	10.5-13	Figure 7, fourth level	Štěrba (1976)
IW1446-03	*Myodes glareolus*	14.5	13-16	Figure 8, fifth level	Štěrba (1976)
IW1446-04	*Myodes glareolus*	15.5	14-17	Figure 9, sixth level	Štěrba (1976)
IW1446-05	*Myodes glareolus*	18.25	18.5-19	Figure 10, seventh level	Štěrba (1976)
IW1446-06	*Myodes glareolus*	18.75	18.5-19	Figure 11, eigth level	Štěrba (1976)
IW1446-07	*Myodes glareolus*	19	16-20	Figure 12, ninth level	Štěrba (1976)
IW1446-08	*Myodes glareolus*	20	17-21	no figure, birth	Štěrba (1976)
IW1446-09	*Myodes glareolus*	29	9d after birth	Eye opening	http://eol.org/pages/1179604/details [2014-04-23]
IW1446-10	*Myodes glareolus*	42.5	20-25d after birth	weaning	http://animaldiversity.ummz.umich.edu/accounts/Myodes_glareolus/ [2014-05-02]
IW1315	*Myotis lucifugus*				
IW1315-01	*Myotis lucifugus*	x	x	stage 1	Adams (1992)
IW1315-02	*Myotis lucifugus*	x	x	stage 2	Adams (1992)
IW1315-03	*Myotis lucifugus*	x	x	stage 3	Adams (1992)
IW1315-04	*Myotis lucifugus*	x	x	stage 4	Adams (1992)
IW1315-05	*Myotis lucifugus*	x	x	stage 5	Adams (1992)
IW1315-06	*Myotis lucifugus*	x	x	stage 6	Adams (1992)
IW1315-07	*Myotis lucifugus*	x	x	stage 7	Adams (1992)
IW1315-08	*Myotis lucifugus*	59.79	59.79 (average)	birth	http://eol.org/pages/327247/overview (2014-02-10)
IW1315-09	*Myotis lucifugus*	62.29	2-3d	Eye opening	http://kufs.ku.edu/libres/Mammals_of_Kansas/myot-lucifugus.html [2014-04-23]
IW1315-10	*Myotis lucifugus*	84.79	25d after birth	weaning	http://genomics.senescence.info/species/entry.php?species=Myotis_lucifugus [2014-05-02]
IW1451	*Myotis myotis*				
IW1451-01	*Myotis myotis*	27	27	stage 3	Štěrba (1990)

IW1451-02	*Myotis myotis*	32	32	stage 4	Štěrba (1990)
IW1451-03	*Myotis myotis*	39	39	stage 5	Štěrba (1990)
IW1451-04	*Myotis myotis*	43	43	stage 6	Štěrba (1990)
IW1451-05	*Myotis myotis*	45	45	stage 7	Štěrba (1990)
IW1451-06	*Myotis myotis*	50	50	stage 8	Štěrba (1990)
IW1451-07	*Myotis myotis*	57	57	stage 9	Štěrba (1990)
IW1451-08	*Myotis myotis*	60	60	stage H	Štěrba (1990)
IW1451-09	*Myotis myotis*	70	70	stage N, birth	Štěrba (1990)
IW1451-10	*Myotis myotis*	77	77	stage E	Štěrba (1990)
IW1451-11	*Myotis myotis*	112	42d after birth	weaning	http://genomics.senescence.info/species/entry.php?species=Myotis_myotis [2014-05-02]
IW1453	*Neomys fodiens*				
IW1453-01	*Neomys fodiens*	21	21, birth	Figure 5, listed by days	Vogel (1972)
IW1453-02	*Neomys fodiens*	22	22	Figure 5, listed by days	Vogel (1972)
IW1453-03	*Neomys fodiens*	23	23	Figure 5, listed by days	Vogel (1972)
IW1453-04	*Neomys fodiens*	24	24	Figure 5, listed by days	Vogel (1972)
IW1453-05	*Neomys fodiens*	25	25	Figure 5, listed by days	Vogel (1972)
IW1453-06	*Neomys fodiens*	26	26	Figure 5, listed by days	Vogel (1972)
IW1453-07	*Neomys fodiens*	27	27	Figure 5, listed by days	Vogel (1972)
IW1453-08	*Neomys fodiens*	28	28	Figure 5, listed by days	Vogel (1972)
IW1453-09	*Neomys fodiens*	33	22d after birth	text	Vogel (1972)
IW1453-10	*Neomys fodiens*	43	32d after birth	weaning	http://genomics.senescence.info/species/entry.php?species=Neomys_fodiens [2014-05-02]
IW1342	*Nycticebus coucang ("tardigradus")*				
IW1342-01	*Nycticebus coucang*	x	x	Plate IV, figure 1	Hubrecht and Keibel (1907)
IW1342-02	*Nycticebus coucang*	x	x	Plate IV, figure 2	Hubrecht and Keibel (1907)
IW1342-03	*Nycticebus coucang*	x	x	Plate IV, figure 3	Hubrecht and Keibel (1907)
IW1342-04	*Nycticebus coucang*	x	x	Plate IV, figure 4	Hubrecht and Keibel (1907)
IW1342-05	*Nycticebus coucang*	x	x	Plate IV, figure 5	Hubrecht and Keibel (1907)
IW1342-06	*Nycticebus coucang*	x	x	Plate IV, figure 6	Hubrecht and Keibel (1907)
IW1342-07	*Nycticebus coucang*	x	x	Plate IV, figure 7	Hubrecht and Keibel (1907)
IW1342-08	*Nycticebus coucang*	x	x	Plate IV, figure 8	Hubrecht and Keibel (1907)
IW1342-09	*Nycticebus coucang*	x	x	Plate IV, figure 9	Hubrecht and Keibel (1907)
IW1342-10	*Nycticebus coucang*	188	188	Birth (estimated eye opening)	http://animaldiversity.ummz.umich.edu/accounts/Nycticebus_coucang/ (2014-02-10)
IW1342-11	*Nycticebus coucang*	365	177 after birth	weaning	http://genomics.senescence.info/species/entry.php?species=Nycticebus_coucang [
IW1332	*Oryctolagus cuniculus* (1) *("Lepus")*				
IW1332-01	*Oryctolagus cuniculus* (1)	0.0625	1,5h after coitus	Figure 1, 13	Minot and Taylor (1905)
IW1332-02	*Oryctolagus cuniculus* (1)	6,5	6,5	Figure 2, 14	Minot and Taylor (1905)
IW1332-03	*Oryctolagus cuniculus* (1)	7,5	7,5	Figure 3, 15	Minot and Taylor (1905)
IW1332-04	*Oryctolagus cuniculus* (1)	8,24	8-6	Figure 4, 16	Minot and Taylor (1905)
IW1332-05	*Oryctolagus cuniculus* (1)	8,26	8-6	Figure 5, 17	Minot and Taylor (1905)
IW1332-06	*Oryctolagus cuniculus* (1)	8,3	8-1	Figure 6, 18	Minot and Taylor (1905)
IW1332-07	*Oryctolagus cuniculus* (1)	8,4	8,5	Figure 7, 19	Minot and Taylor (1905)
IW1332-08	*Oryctolagus cuniculus* (1)	8,6	8,5	Figure 8, 20	Minot and Taylor (1905)
IW1332-09	*Oryctolagus cuniculus* (1)	9	9	Figure 9, 21	Minot and Taylor (1905)
IW1332-10	*Oryctolagus cuniculus* (1)	9,5	9,5	Figure 10, 22	Minot and Taylor (1905)

IW1332-11	*Oryctolagus cuniculus* (1)	10	10	Figure 11, 23	Minot and Taylor (1905)
IW1332-12	*Oryctolagus cuniculus* (1)	10,5	10,5	Figure 12, 24	Minot and Taylor (1905)
IW1332-13	*Oryctolagus cuniculus* (1)	11	11	Figure 25	Minot and Taylor (1905)
IW1332-14	*Oryctolagus cuniculus* (1)	11,5	11,5	Figure 26	Minot and Taylor (1905)
IW1332-15	*Oryctolagus cuniculus* (1)	12	12	Figure 27	Minot and Taylor (1905)
IW1332-16	*Oryctolagus cuniculus* (1)	12,5	12,5	Figure 28	Minot and Taylor (1905)
IW1332-17	*Oryctolagus cuniculus* (1)	13	13	Figure 29	Minot and Taylor (1905)
IW1332-18	*Oryctolagus cuniculus* (1)	14	14	Figure 30	Minot and Taylor (1905)
IW1332-19	*Oryctolagus cuniculus* (1)	15	15	Figure 31	Minot and Taylor (1905)
IW1332-20	*Oryctolagus cuniculus* (1)	16	16	Figure 32	Minot and Taylor (1905)
IW1332-21	*Oryctolagus cuniculus* (1)	16,5	16,5	Figure 33	Minot and Taylor (1905)
IW1332-22	*Oryctolagus cuniculus* (1)	17	17	Figure 34	Minot and Taylor (1905)
IW1332-23	*Oryctolagus cuniculus* (1)	18	18	Figure 35	Minot and Taylor (1905)
IW1332-24	*Oryctolagus cuniculus* (1)	20	20	Figure 36	Minot and Taylor (1905)
IW1332-25	*Oryctolagus cuniculus* (1)	29	28-30	birth	Williams et al. (1995)
IW1332-26	*Oryctolagus cuniculus* (1)	37.5	7-10 after birth	Eye opening	http://www.feral.org.au/wp-content/uploads/2012/02/PS_rabbit_factsheet.pdf [2014-04-24]
IW1332-27	*Oryctolagus cuniculus* (1)	55	26d after birth	weaning	http://genomics.senescence.info/species/entry.php?species=Oryctolagus_cuniculus [2014-05-02]
IW1343	*Oryctolagus cuniculus* (2)				
IW1343-01	*Oryctolagus cuniculus* (2)	5	5	bilaminar embryonic disc	Butler and Juurlink (1987)
IW1343-02	*Oryctolagus cuniculus* (2)	6	6	primitive streak	Butler and Juurlink (1987)
IW1343-03	*Oryctolagus cuniculus* (2)	7.975	7.7-8.25	stage 9	Butler and Juurlink (1987)
IW1343-05	*Oryctolagus cuniculus* (2)	8.625	8.25-9	stage 10	Butler and Juurlink (1987)
IW1343-06	*Oryctolagus cuniculus* (2)	9.375	9-9.75	stage 11	Butler and Juurlink (1987)
IW1343-08	*Oryctolagus cuniculus* (2)	10.375	9.75-11	stage 12	Butler and Juurlink (1987)
IW1343-10	*Oryctolagus cuniculus* (2)	11.5	11.5	stage 13/14	Butler and Juurlink (1987)
IW1343-11	*Oryctolagus cuniculus* (2)	12.5	12.5	stage 15	Butler and Juurlink (1987)
IW1343-12	*Oryctolagus cuniculus* (2)	13.5	13.5	stage 16	Butler and Juurlink (1987)
IW1343-13	*Oryctolagus cuniculus* (2)	14.5	14.5	stage 17/18	Butler and Juurlink (1987)
IW1343-14	*Oryctolagus cuniculus* (2)	15.5	15.5	stage 19	Butler and Juurlink (1987)
IW1343-15	*Oryctolagus cuniculus* (2)	16.5	16.5	stage 20/21	Butler and Juurlink (1987)
IW1343-16	*Oryctolagus cuniculus* (2)	17.25	17-17.5	stage 22	Butler and Juurlink (1987)
IW1343-18	*Oryctolagus cuniculus* (2)	18.125	17.5-18.75	stage 23	Butler and Juurlink (1987)
IW1343-19	*Oryctolagus cuniculus* (2)	18.5	18.5	-	Beaudoin et al. (2003)
IW1343-20	*Oryctolagus cuniculus* (2)	19.5	19.5	-	Beaudoin et al. (2003)
IW1343-21	*Oryctolagus cuniculus* (2)	29	28-30	birth	Williams et al. (1995)
IW1343-22	*Oryctolagus cuniculus* (2)	37.5	7-10 after birth	Eye opening	http://www.feral.org.au/wp-content/uploads/2012/02/PS_rabbit_factsheet.pdf [2014-

					04-24]
IW1343-23	*Oryctolagus cuniculus* (2)	55	26d after birth	weaning	http://genomics.senescence.info/species/entry.php?species=Oryctolagus_cuniculus [2014-05-02]
IW1308	*Ovis aries*				
IW1308-01	*Ovis aries*	13.5	13-14 (primitive streak)	x/Fig. 3-4/S. 33	Butler and Juurlink (1987), Bryden et al. (1972), Evans and Sack (1973)
IW1308-02	*Ovis aries*	14.5	14-15 (1-3somites)	x/Fig. 8.12	Butler and Juurlink 1987 / Rüsse 1991
IW1308-03	*Ovis aries*	15.5	15-16 (4-12 somites)	/Figure 100	Butler and Juurlink 1987
IW1308-04	*Ovis aries*	16.75	17	page 33	Evans and Sack (1973)
IW1308-05	*Ovis aries*	17	17/N.N.	Abb. 8.12 / Abb. 10.11	Rüsse (1991a) / Rüsse and Sinowatz (1991)
IW1308-06	*Ovis aries*	17.25	17	Abb. 18.4D	Rüsse (1991b)
IW1308-07	*Ovis aries*	18	18	Fig. 6 = Figure 101	Bryden et al. 1972 = Butler and Juurlink (1987)
IW1308-08	*Ovis aries*	19	19	page 33	Evans and Sack (1973)
IW1308-09	*Ovis aries*	19.75	20	Fig. 7 = Figure 102	Bryden et al. 1972 = Butler and Juurlink (1987)
IW1308-10	*Ovis aries*	20.25	20	Abb. 22.13	Schnorr and Kressin (2006)
IW1308-11	*Ovis aries*	20.75	21	41487	Rüsse (1991a)
IW1308-12	*Ovis aries*	21.25	21	Abb. 18.2A	Rüsse (1991b)
IW1308-13	*Ovis aries*	22	22	Fig. 8 = Figure 103	Bryden et al. 1972 = Butler and Juurlink (1987)
IW1308-14	*Ovis aries*	23.75	24	Abb. 18.4E	Rüsse (1991b)
IW1308-15	*Ovis aries*	24	24	Fig. 9 = Figure 104	Bryden et al. 1972 = Butler and Juurlink (1987)
IW1308-16	*Ovis aries*	24.25	24	Abb. 18.2B	Rüsse (1991b)
IW1308-17	*Ovis aries*	24.75	26	Abb. 18.4F	Rüsse (1991b)
IW1308-18	*Ovis aries*	25	25	Abb. 18.2C	Rüsse (1991b)
IW1308-19	*Ovis aries*	26	26	Fig. 10 = Figure 105	Bryden et al. 1972 = Butler and Juurlink (1987)
IW1308-20	*Ovis aries*	28	28	Fig. 11 = Figure 106	Bryden et al. 1972 = Butler and Juurlink (1987)
IW1308-21	*Ovis aries*	30	30	Fig. 12 = Figure 107	Bryden et al. 1972 = Butler and Juurlink (1987)
IW1308-22	*Ovis aries*	31	31	Abb. 18.4G	Rüsse (1991b)
IW1308-23	*Ovis aries*	32	32	page 33	Evans and Sack (1973)
IW1308-24	*Ovis aries*	34	34	Fig. 13 = Figure 108	Bryden et al. 1972 = Butler and Juurlink (1987)
IW1308-25	*Ovis aries*	35	35 (5 weeks)	Abb. 13.4, 13.13	Rüsse and Sinowatz (1991)
IW1308-26	*Ovis aries*	40	40	page 33	Evans and Sack (1973)
IW1308-27	*Ovis aries*	42	42 (6 weeks)	Abb. 13.14	Rüsse and Sinowatz (1991)
IW1308-28	*Ovis aries*	43	43	Table	Evans and Sack (1973)
IW1308-29	*Ovis aries*	57	57	Table	Evans and Sack (1973)
IW1308-30	*Ovis aries*	64	64	Table	Evans and Sack (1973)
IW1308-31	*Ovis aries*	65	60-67	Table	Evans and Sack (1973)
IW1308-32	*Ovis aries*	66	66	Table	Evans and Sack (1973)
IW1308-33	*Ovis aries*	69.5	70	Table	Evans and Sack (1973)
IW1308-34	*Ovis aries*	70.5	70-77	Table	Evans and Sack (1973)
IW1308-35	*Ovis aries*	79	75-90	Table	Evans and Sack (1973)
IW1308-36	*Ovis aries*	80	80	Table	Evans and Sack (1973)
IW1308-37	*Ovis aries*	104	104	Abb. 18.4H/Table	Rüsse (1991b), Evans and Sack (1973)
IW1308-38	*Ovis aries*	116	116	Table	Evans and Sack (1973)
IW1308-39	*Ovis aries*	119	119-126	Table	Evans and Sack (1973)
IW1308-40	*Ovis aries*	120	120	Table	Evans and Sack (1973)
IW1308-41	*Ovis aries*	151	147-155	birth	Evans and Sack (1973)
IW1308-42	*Ovis aries*	333	182 after birth	weaning	http://genomics.senescence.info/species/entry.php?species=Ovis_aries [2014-05-02]
IW1345	*Papio cynocephalus*				
IW1345-01	*Papio cynocephalus*	17	16-18	stage 7	Hendrickx (1971); summarized by Butler and Juurlink (1987)
IW1345-02	*Papio cynocephalus*	23	23 +/-1	stage 9	Hendrickx (1971); summarized by Butler and Juurlink (1987)
IW1345-03	*Papio cynocephalus*	25	25 +/-1	stage 10	Hendrickx (1971); summarized by Butler and Juurlink (1987)
IW1345-04	*Papio cynocephalus*	27	27 +/-1	stage 11	Hendrickx (1971); summarized by Butler and

					Juurlink (1987)
IW1345-05	*Papio cynocephalus*	28	28 +/-1	stage 12	Hendrickx (1971); summarized by Butler and Juurlink (1987)
IW1345-06	*Papio cynocephalus*	29	29 +/-1	stage 13	Hendrickx (1971); summarized by Butler and Juurlink (1987)
IW1345-07	*Papio cynocephalus*	30	30 +/-1	stage 14	Hendrickx (1971); summarized by Butler and Juurlink (1987)
IW1345-08	*Papio cynocephalus*	30.5	31 +/-1	stage 15 (early)	Hendrickx (1971); summarized by Butler and Juurlink (1987)
IW1345-09	*Papio cynocephalus*	31.5	31 +/-1	stage 15 (late)	Hendrickx (1971); summarized by Butler and Juurlink (1987)
IW1345-10	*Papio cynocephalus*	33	33 +/-1	stage 16	Hendrickx (1971); summarized by Butler and Juurlink (1987)
IW1345-11	*Papio cynocephalus*	34.5	35 +/-1	stage 17 (early)	Hendrickx (1971); summarized by Butler and Juurlink (1987)
IW1345-12	*Papio cynocephalus*	35.5	35 +/-1	stage 17 (late)	Hendrickx (1971); summarized by Butler and Juurlink (1987)
IW1345-13	*Papio cynocephalus*	36.5	37 +/-1	stage 18 (early)	Hendrickx (1971); summarized by Butler and Juurlink (1987)
IW1345-14	*Papio cynocephalus*	37.5	37 +/-1	stage 18 (late)	Hendrickx (1971); summarized by Butler and Juurlink (1987)
IW1345-15	*Papio cynocephalus*	39	39 +/-1	stage 19	Hendrickx (1971); summarized by Butler and Juurlink (1987)
IW1345-16	*Papio cynocephalus*	41	41 +/-1	stage 20	Hendrickx (1971); summarized by Butler and Juurlink (1987)
IW1345-17	*Papio cynocephalus*	43	43 +/-1	stage 21	Hendrickx (1971); summarized by Butler and Juurlink (1987)
IW1345-18	*Papio cynocephalus*	45	45 +/-1	stage 22	Hendrickx (1971); summarized by Butler and Juurlink (1987)
IW1345-19	*Papio cynocephalus*	47	47 +/-1	stage 23	Hendrickx (1971); summarized by Butler and Juurlink (1987)
IW1345-20	*Papio cynocephalus*	180	180	Birth (estimated eye opening)	http://pin.primate.wisc.edu/factsheets/entry/ yellow_baboon (2014-01-23)
IW1345-21	*Papio cynocephalus*	540	Almost a year after birth	weaning	Rhine et al. (1985)
IW1317	*Pecari tajacu ("Dicotyles")*				
IW1317-01	*Pecari tajacu*	38	38	just text description	Smith and Sowls (1975)
IW1317-02	*Pecari tajacu*	49	49	figure 1A	Smith and Sowls (1975)
IW1317-03	*Pecari tajacu*	60	60	figure 1B	Smith and Sowls (1975)
IW1317-04	*Pecari tajacu*	80	80	figure 1C	Smith and Sowls (1975)
IW1317-05	*Pecari tajacu*	100	100	figure 1D, 2	Smith and Sowls (1975)
IW1317-06	*Pecari tajacu*	120	120	figures 1E, 2	Smith and Sowls (1975)
IW1317-07	*Pecari tajacu*	140	140	figure 1F	Smith and Sowls (1975)
IW1317-08	*Pecari tajacu*	145.5	142-149	Birth (estimated eye opening)	cited by Smith and Sowls (1975)
IW1317-09	*Pecari tajacu*	194.5	49 after birth	weaning	http://genomics.senescence.info/species/entry.php?species=Pecari_tajacu [2014-05-02]
IW1319	*Phocoena phocoena phocoena ("communis")*	30.42d = 1 month			
IW1319-01	*Phocoena phocoena phocoena*	x	x	embryo A, figure 1-3	Müller (1920)
IW1319-02	*Phocoena phocoena phocoena*	x	x	embryo B, figure 4-6, 10	Müller (1920)
IW1319-03	*Phocoena phocoena*	x	x	figure 50a / figure 4	Keibel (1906) after Guldberg (1894, 1899) / Gill (1926)

	phocoena				after Guldberg and Nansen (1894)
IW1319-04	*Phocoena phocoena phocoena*	x	x	figure 50b	Keibel (1906) after Guldberg (1894, 1899)
IW1319-05	*Phocoena phocoena phocoena*	x	x	figure 50c after plate 15, figure 1-4	Keibel (1906) after Kükenthal (1893)
IW1319-06	*Phocoena phocoena phocoena*	x	x	figure 50e after plate 14, figure 15	Keibel (1906) after Kükenthal (1893)
IW1319-07	*Phocoena phocoena phocoena*	x	x	figure 42	Kükenthal (1893)
IW1319-08	*Phocoena phocoena phocoena*	334.62	11 months	birth	http://animaldiversity.ummz.umich.edu/accounts/Phocoena_phocoena/ (2014-02-10)
IW1319-08	*Phocoena phocoena phocoena*	577.62	243 after birth	weaning	http://genomics.senescence.info/species/entry.php?species=Phocoena_phocoena [2014-05-02]
IW1320	*Pipistrellus abramus*				
IW1320-01	*Pipistrellus abramus*	x	x	stage 10, figure 2a-b	Tokita (2006)
IW1320-02	*Pipistrellus abramus*	x	x	stage 11A, figure 2c-d	Tokita (2006)
IW1320-03	*Pipistrellus abramus*	x	x	stage 11B, figure 2e-f	Tokita (2006)
IW1320-04	*Pipistrellus abramus*	x	x	stage 12, figure 2g-h	Tokita (2006)
IW1320-05	*Pipistrellus abramus*	x	x	stage 13A, figure 2i-l	Tokita (2006)
IW1320-06	*Pipistrellus abramus*	x	x	stage 13B, figure 3a-c	Tokita (2006)
IW1320-07	*Pipistrellus abramus*	x	x	stage 14, figures 3d-f, 5a-b	Tokita (2006)
IW1320-08	*Pipistrellus abramus*	x	x	stage 15, figures 3g-i, 5c-d	Tokita (2006)
IW1320-09	*Pipistrellus abramus*	x	x	stage 16, figures 3j-l, 5e-f	Tokita (2006)
IW1320-10	*Pipistrellus abramus*	x	x	stage 17, figures 4a-d, 5g-h	Tokita (2006)
IW1320-11	*Pipistrellus abramus*	x	x	stage 18, figures 4e-g, 5i-j	Tokita (2006)
IW1320-12	*Pipistrellus abramus*	x	x	stage 20, figure 4h-j	Tokita (2006)
IW1320-13	*Pipistrellus abramus*	x	x	stage 24, figure 4k-m	Tokita (2006)
IW1320-14	*Pipistrellus abramus*	x	x	birth	-
IW1320-15	*Pipistrellus abramus*	x	8-9d after birth	eye opening	Morii (1980)
IW1300	*Rangifer tarandus*				
IW1300-01	*Rangifer tarandus*	31.5	ca. 4.5 months after fertilization	Embryo B, plate 25, figure 1-6	Retzius (1900)
IW1300-02	*Rangifer tarandus*	35.3	x	Embryo D, plate 26, figures 1-5	Retzius (1900)
IW1300-03	*Rangifer tarandus*	39.2	x	Embryo E, plate 27, figures 1-2	Retzius (1900)
IW1300-04	*Rangifer tarandus*	43	x	Embryo F, plate 27, figure 3	Retzius (1900)
IW1300-05	*Rangifer tarandus*	46.8	x	Embryo G, plate 27, figures 4-5	Retzius (1900)
IW1300-06	*Rangifer tarandus*	50.6	x	Embryo H, plate 28, figures 1-2	Retzius (1900)
IW1300-07	*Rangifer tarandus*	54.5	21-25d after initial organ formation	Embryo I, plate 28, figures 3-4	Retzius (1900)
IW1300-08	*Rangifer tarandus*	280	birth, 40 weeks after fertilisation	Text (estimated time of eye opening)	Retzius (1900)
IW1300-09	*Rangifer tarandus*	400	120 after birth	weaning	http://genomics.senescence.info/species/entry.php?species=Rangifer_tarandus [2014-05-02]
IW1350	*Rattus norvegicus*	d	d-h		
IW1350-01	*Rattus norvegicus*	8.5	8.5d	page 16-17	Evans and Sack (1973)
IW1350-02	*Rattus norvegicus*	8.85	10	E 25	Henneberg (1937)

IW1350-03	*Rattus norvegicus*	9.2	10	E 35	Henneberg (1937)
IW1350-04	*Rattus norvegicus*	9.55	11	E 36	Henneberg (1937)
IW1350-05	*Rattus norvegicus*	9.9	11	E 39	Henneberg (1937)
IW1350-06	*Rattus norvegicus*	10.25	10-6	E 40	Henneberg (1937)
IW1350-07	*Rattus norvegicus*	10.32	10-10	E 45	Henneberg (1937), Evans and Sack (1973)
IW1350-08	*Rattus norvegicus*	10.39	10-10	E 46	Henneberg (1937)
IW1350-09	*Rattus norvegicus*	10.46	10-11	E 49	Henneberg (1937)
IW1350-10	*Rattus norvegicus*	10.58	10-14	E 52a	Henneberg (1937)
IW1350-11	*Rattus norvegicus*	10.75	10-18	E 53	Henneberg (1937)
IW1350-12	*Rattus norvegicus*	11	11	E 54	Henneberg (1937)
IW1350-13	*Rattus norvegicus*	11.6	11-6	E 58	Henneberg (1937)
IW1350-14	*Rattus norvegicus*	11.66	11-13	E 61	Henneberg (1937)
IW1350-15	*Rattus norvegicus*	11.72	11-13	E 62	Henneberg (1937), Evans and Sack (1973)
IW1350-16	*Rattus norvegicus*	11.79	11-19	E 68	Henneberg (1937)
IW1350-17	*Rattus norvegicus*	12.25	12-6	E 71	Henneberg (1937)
IW1350-18	*Rattus norvegicus*	12.5	12-12	E 78	Henneberg (1937)
IW1350-19	*Rattus norvegicus*	13	13	E 83	Henneberg (1937)
IW1350-20	*Rattus norvegicus*	13.5	13-12	E 85	Henneberg (1937)
IW1350-21	*Rattus norvegicus*	14	14	E 96	Henneberg (1937)
IW1350-22	*Rattus norvegicus*	14.58	14-14	E 102	Henneberg (1937)
IW1350-23	*Rattus norvegicus*	15	15	E 104	Henneberg (1937)
IW1350-24	*Rattus norvegicus*	15.54	15-13	E 109	Henneberg (1937)
IW1350-25	*Rattus norvegicus*	16.02	16	E 118	Henneberg (1937), Evans and Sack (1973)
IW1350-26	*Rattus norvegicus*	16.51	16	E 123	Henneberg (1937)
IW1350-27	*Rattus norvegicus*	17	17	E 124	Henneberg (1937)
IW1350-28	*Rattus norvegicus*	17.5	17-12	E 125	Henneberg (1937)
IW1350-29	*Rattus norvegicus*	18.5	18-12	E 126	Henneberg (1937)
IW1350-30	*Rattus norvegicus*	19.5	19-12	E 127	Henneberg (1937), Evans and Sack (1973)
IW1350-31	*Rattus norvegicus*	19.83	19-20	E 128	Henneberg (1937)
IW1350-32	*Rattus norvegicus*	22	22	birth	Evans and Sack (1973)
IW1350-33	*Rattus norvegicus*	35	35	Eye opening	Müller (1972)
IW1350-34	*Rattus norvegicus*	47	25 after birth	weaning	http://genomics.senescence.info/species/entry.php?species=Rattus_norvegicus [204-05-02]
IW1321	*Scotophilus kuhlii ("temminckii")*				
IW1321-01	*Scotophilus kuhlii temminckii*	x	x	Embryo (E) 1	Koike (1924)
IW1321-02	*Scotophilus kuhlii temminckii*	x	x	Embryo (E) 2	Koike (1924)
IW1321-03	*Scotophilus kuhlii temminckii*	x	x	Embryo (E) 3	Koike (1924)
IW1321-04	*Scotophilus kuhlii temminckii*	x	x	Embryo (E) 4	Koike (1924)
IW1321-05	*Scotophilus kuhlii temminckii*	x	x	Embryo (E) 5	Koike (1924)
IW1321-06	*Scotophilus kuhlii temminckii*	x	x	Embryo (E) 6	Koike (1924)
IW1321-07	*Scotophilus kuhlii temminckii*	x	x	Embryo (E) 7	Koike (1924)
IW1321-08	*Scotophilus kuhlii temminckii*	x	x	Embryo (E) 8	Koike (1924)
IW1321-09	*Scotophilus kuhlii temminckii*	x	x	Embryo (E) 9	Koike (1924)
IW1321-10	*Scotophilus kuhlii temminckii*	x	x	Embryo (E) 10	Koike (1924)
IW1321-11	*Scotophilus kuhlii temminckii*	x	x	Embryo (E) 11	Koike (1924)
IW1321-12	*Scotophilus kuhlii temminckii*	x	x	Embryo (E) 12	Koike (1924)
IW1321-13	*Scotophilus kuhlii temminckii*	x	x	Embryo (E) 13	Koike (1924)
IW1321-14	*Scotophilus kuhlii temminckii*	x	x	Embryo (E) 14	Koike (1924)
IW1321-15	*Scotophilus kuhlii temminckii*	x	x	Embryo (E) 15	Koike (1924)
IW1321-16	*Scotophilus kuhlii temminckii*	x	x	Embryo (E) 16	Koike (1924)
IW1321-17	*Scotophilus kuhlii temminckii*	x	x	Embryo (E) 17	Koike (1924)
IW1321-18	*Scotophilus kuhlii*	x	x	Embryo (E) 18	Koike (1924)

	temminckii				
IW1321-19	*Scotophilus kuhlii temminckii*	x	x	Embryo (E) 19	Koike (1924)
IW1321-20	*Scotophilus kuhlii temminckii*	x	x	Embryo (E) 20	Koike (1924)
IW1321-21	*Scotophilus kuhlii temminckii*	x	x	Embryo (E) 21	Koike (1924)
IW1321-22	*Scotophilus kuhlii temminckii*	x	x	Embryo (E) 22	Koike (1924)
IW1321-23	*Scotophilus kuhlii temminckii*	x	x	Embryo (E) 23	Koike (1924)
IW1321-24	*Scotophilus kuhlii temminckii*	110	110	birth	http://eol.org/pages/310390/overview (2014-02-10)
IW1452	*Sorex araneus*				
IW1452-01	*Sorex araneus*	13	13	third level	Štěrba (1977a)
IW1452-02	*Sorex araneus*	16	16	fifth level	Štěrba (1977a)
IW1452-03	*Sorex araneus*	18.5	18.5	sixth level	Štěrba (1977a)
IW1452-04	*Sorex araneus*	20	20	seventh level	Štěrba (1977a)
IW1452-05	*Sorex araneus*	21	21, birth	Figure 4, listed by day / new born	Vogel (1972) / Štěrba (1977a)
IW1452-06	*Sorex araneus*	22	22	Figure 4, listed by day	Vogel (1972)
IW1452-07	*Sorex araneus*	23	23	Figure 4, listed by day	Vogel (1972)
IW1452-08	*Sorex araneus*	24	24	Figure 4, listed by day	Vogel (1972)
IW1452-09	*Sorex araneus*	25	25	Figure 4, listed by day	Vogel (1972)
IW1452-10	*Sorex araneus*	26	26	Figure 4, listed by day	Vogel (1972)
IW1452-11	*Sorex araneus*	27	27	Figure 4, listed by day	Vogel (1972)
IW1452-12	*Sorex araneus*	28	28	Figure 4, listed by day	Vogel (1972)
IW1452-13	*Sorex araneus*	29	29	Figure 4, listed by day	Vogel (1972)
IW1452-14	*Sorex araneus*	42	21d after birth	Text, eye opening	Vogel (1972)
IW1452-15	*Sorex araneus*	46	25d after bith	weaning	http://genomics.senescence.info/species/entry.php?species=Sorex_araneus [2014-05-02]
IW1279	*Spermophilus citellus*		d		
IW1279-01	*Spermophilus citellus*	x	x	Figure 1	Völker-Brünn (1922)
IW1279-02	*Spermophilus citellus*	x	x	Figure 2	Völker-Brünn (1922)
IW1279-03	*Spermophilus citellus*	x	x	Figure 3	Völker-Brünn (1922)
IW1279-04	*Spermophilus citellus*	x	x	Figure 4	Völker-Brünn (1922)
IW1279-05	*Spermophilus citellus*	x	x	Figure 5	Völker-Brünn (1922)
IW1279-06	*Spermophilus citellus*	x	x	Figure 6	Völker-Brünn (1922)
IW1279-07	*Spermophilus citellus*	x	x	Figure 7	Völker-Brünn (1922)
IW1279-08	*Spermophilus citellus*	x	x	Figure 8	Völker-Brünn (1922)
IW1279-09	*Spermophilus citellus*	x	x	Figure 9	Völker-Brünn (1922)
IW1279-10	*Spermophilus citellus*	x	x	Figure 10	Völker-Brünn (1922)
IW1279-11	*Spermophilus citellus*	x	x	Figure 11	Völker-Brünn (1922)
IW1279-12	*Spermophilus citellus*	x	x	Figure 12	Völker-Brünn (1922)
IW1279-13	*Spermophilus citellus*	x	x	Figure 13	Völker-Brünn (1922)
IW1279-14	*Spermophilus citellus*	x	x	Figure 14	Völker-Brünn (1922)
IW1279-15	*Spermophilus citellus*	x	x	Figure 15	Völker-Brünn (1922)
IW1279-16	*Spermophilus citellus*	x	x	Figure 16	Völker-Brünn (1922)
IW1279-17	*Spermophilus citellus*	x	x	Figure 17	Völker-Brünn (1922)
IW1279-18	*Spermophilus citellus*	x	x	Figure 18	Völker-Brünn (1922)
IW1279-19	*Spermophilus citellus*	x	x	Figure 19	Völker-Brünn (1922)
IW1279-20	*Spermophilus citellus*	x	x	Figure 20	Völker-Brünn (1922)
IW1279-21	*Spermophilus citellus*	x	x	Figure 21	Völker-Brünn (1922)

IW1279-22	*Spermophilus citellus*	x	x	Figure 22	Völker-Brünn (1922)
IW1279-23	*Spermophilus citellus*	x	x	Figure 23	Völker-Brünn (1922)
IW1279-24	*Spermophilus citellus*	x	x	Figure 24	Völker-Brünn (1922)
IW1279-25	*Spermophilus citellus*	x	x	Figure 25	Völker-Brünn (1922)
IW1279-26	*Spermophilus citellus*	x	x	Figure 26	Völker-Brünn (1922)
IW1279-27	*Spermophilus citellus*	x	x	Figure 27	Völker-Brünn (1922)
IW1279-28	*Spermophilus citellus*	x	x	Figure 28	Völker-Brünn (1922)
IW1279-29	*Spermophilus citellus*	x	x	Figure 29	Völker-Brünn (1922)
IW1279-30	*Spermophilus citellus*	x	x	Figure 30	Völker-Brünn (1922)
IW1279-31	*Spermophilus citellus*	x	x	Figure 31	Völker-Brünn (1922)
IW1279-32	*Spermophilus citellus*	x	x	Figure 32	Völker-Brünn (1922)
IW1279-33	*Spermophilus citellus*	x	x	Figure 33	Völker-Brünn (1922)
IW1279-34	*Spermophilus citellus*	x	x	Figure 34	Völker-Brünn (1922)
IW1279-35	*Spermophilus citellus*	x	x	Figure 35	Völker-Brünn (1922)
IW1279-36	*Spermophilus citellus*	x	x	Figure 36	Völker-Brünn (1922)
IW1279-37	*Spermophilus citellus*	x	x	Figure 37	Völker-Brünn (1922)
IW1279-38	*Spermophilus citellus*	x	x	Figure 38	Völker-Brünn (1922)
IW1279-39	*Spermophilus citellus*	x	x	Figure 39	Völker-Brünn (1922)
IW1279-40	*Spermophilus citellus*	x	x	Figure 40	Völker-Brünn (1922)
IW1279-41	*Spermophilus citellus*	x	x	Figure 41	Völker-Brünn (1922)
IW1279-42	*Spermophilus citellus*	x	x	Figure 42	Völker-Brünn (1922)
IW1279-43	*Spermophilus citellus*	x	x	Figure 43	Völker-Brünn (1922)
IW1279-44	*Spermophilus citellus*	x	x	Figure 44	Völker-Brünn (1922)
IW1279-45	*Spermophilus citellus*	x	x	Figure 45	Völker-Brünn (1922)
IW1279-46	*Spermophilus citellus*	x	x	Figure 46	Völker-Brünn (1922)
IW1279-47	*Spermophilus citellus*	x	x	Figure 47	Völker-Brünn (1922)
IW1279-48	*Spermophilus citellus*	x	x	Figure 48	Völker-Brünn (1922)
IW1279-49	*Spermophilus citellus*	x	x	Figure 49	Völker-Brünn (1922)
IW1279-50	*Spermophilus citellus*	25.5	25-26	birth	Parker (1990)
IW1279-51	*Spermophilus citellus*	53.5	4 weeks	Eye opening	http://eol.org/pages/1041051/details
IW1279-52	*Spermophilus citellus*	59,5	34d after birth	weaning	http://genomics.senescence.info/species/entry.php?species=Spermophilus_citellus [2014-05-02]
IW1283	*Stenella attenuata*				
IW1283-01	*Stenella attenuata*	12.75	13	9/1	Thewissen and Heyning (2007) (stage 8 not coded as no time information resp. no other info than primitive disk) / Štĕrba et al. (2000); age reference = Štĕrba et al. (2000)
IW1283-02	*Stenella attenuata*	13.25	13	10/1	Thewissen and Heyning (2007) / Štĕrba et al. (2000); Štĕrba et al. (2000) = TIME REFERENCE
IW1283-03	*Stenella attenuata*	13.75	14	11/2	Thewissen and Heyning

					(2007) / Štěrba et al. (2000); age reference = Štěrba et al. (2000)
IW1283-04	*Stenella attenuata*	14	14	12/2	Thewissen and Heyning (2007) / Štěrba et al. (2000); age reference = Štěrba et al. (2000)
IW1283-05	*Stenella attenuata*	14.25	14	13/2	Thewissen and Heyning (2007) / Štěrba et al. (2000); age reference = Štěrba et al. (2000)
IW1283-06	*Stenella attenuata*	23.5	22-28, mean=24	14/3	Thewissen and Heyning (2007) / Štěrba et al. (2000); age reference = Štěrba et al. (2000)
IW1283-07	*Stenella attenuata*	24.5	22-28, mean=24	15/3	Thewissen and Heyning (2007) / Štěrba et al. (2000); age reference = Štěrba et al. (2000)
IW1283-08	*Stenella attenuata*	30	27-36, mean=30	16/4	Thewissen and Heyning (2007) / Štěrba et al. (2000); age reference = Štěrba et al. (2000)
IW1283-09	*Stenella attenuata*	38	32-42, mean=38	17/5	Thewissen and Heyning (2007) / Štěrba et al. (2000); age reference = Štěrba et al. (2000)
IW1283-10	*Stenella attenuata*	45.5	41-52, mean=46	18/6 / 1a	Thewissen and Heyning (2007) / Štěrba et al. (2000); age reference = Štěrba et al. (2000)
IW1283-11	*Stenella attenuata*	46.5	41-52, mean=46	19/6 / 1b	Thewissen and Heyning (2007) / Štěrba et al. (2000); age reference = Štěrba et al. (2000)
IW1283-12	*Stenella attenuata*	57	51-66, mean=57	20/7 / 1c	Thewissen and Heyning (2007) / Štěrba et al. (2000); age reference = Štěrba et al. (2000)
IW1283-13	*Stenella attenuata*	70	62-78, mean=70	20/8	Thewissen and Heyning (2007) / Štěrba et al. (2000); age reference = Štěrba et al. (2000)
IW1283-14	*Stenella attenuata*	89	78-100	21/9	Thewissen and Heyning (2007) / Štěrba et al. (2000); age reference = Štěrba et al. (2000)
IW1283-15	*Stenella attenuata*	105	102-108	22/12	Thewissen and Heyning (2007) / Štěrba et al. (2000); age reference = Štěrba et al. (2000)
IW1283-16	*Stenella attenuata*	140	105-175	23/10 / 1d	Thewissen and Heyning (2007) / Štěrba et al. (2000); age reference = Štěrba et al. (2000)
IW1283-17	*Stenella attenuata*	240	190-290	23/11	Thewissen and Heyning (2007) / Štěrba et al. (2000); age reference = Štěrba et al. (2000)
IW1283-18	*Stenella attenuata*	280	270-290	birth: mentioned in text: chapter 6.2	Thewissen and Heyning (2007) / Štěrba et al. (2000); age reference = Štěrba et al. (2000)
IW1283-19	*Stenella attenuata*	823	543 after birth	weaning	http://genomics.senescence.info/species/entry.php?species=Stenella_attenuata [2014-05-02]
IW1284	*Stenella longirostris*				
IW1284-01	*Stenella longirostris*	12.75	13	as for *Stenella attenuata*	Thewissen and Heyning (2007) / Štěrba et al. (2000); age reference = Štěrba et al. (2000)
IW1284-02	*Stenella longirostris*	13.25	13	as for *Stenella attenuata*	Thewissen and Heyning (2007) / Štěrba et al. (2000); age reference = Štěrba et al. (2000)

IW1284-03	*Stenella longirostris*	13.75	14	as for *Stenella attenuata*	Thewissen and Heyning (2007) / Štěrba et al. (2000); age reference = Štěrba et al. (2000)
IW1284-04	*Stenella longirostris*	14	14	as for *Stenella attenuata*	Thewissen and Heyning (2007) / Štěrba et al. (2000); age reference = Štěrba et al. (2000)
IW1284-05	*Stenella longirostris*	14.25	14	as for *Stenella attenuata*	Thewissen and Heyning (2007) / Štěrba et al. (2000); age reference = Štěrba et al. (2000)
IW1284-06	*Stenella longirostris*	23.5	22-28, mean=24	as for *Stenella attenuata*	Thewissen and Heyning (2007) / Štěrba et al. (2000); age reference = Štěrba et al. (2000)
IW1284-07	*Stenella longirostris*	24.5	22-28, mean=24	as for *Stenella attenuata*	Thewissen and Heyning (2007) / Štěrba et al. (2000); age reference = Štěrba et al. (2000)
IW1284-08	*Stenella longirostris*	30	27-36, mean=30	as for *Stenella attenuata*	Thewissen and Heyning (2007) / Štěrba et al. (2000); age reference = Štěrba et al. (2000)
IW1284-09	*Stenella longirostris*	38	32-42, mean=38	as for *Stenella attenuata*	Thewissen and Heyning (2007) / Štěrba et al. (2000); age reference = Štěrba et al. (2000)
IW1284-10	*Stenella longirostris*	45.5	41-52, mean=46	as for *Stenella attenuata*	Thewissen and Heyning (2007) / Štěrba et al. (2000); age reference = Štěrba et al. (2000)
IW1284-11	*Stenella longirostris*	46.5	41-52, mean=46	as for *Stenella attenuata*	Thewissen and Heyning (2007) / Štěrba et al. (2000); age reference = Štěrba et al. (2000)
IW1284-12	*Stenella longirostris*	57	51-66, mean=57	as for *Stenella attenuata*	Thewissen and Heyning (2007) / Štěrba et al. (2000); age reference = Štěrba et al. (2000)
IW1284-13	*Stenella longirostris*	70	62-78, mean=70	as for *Stenella attenuata*	Thewissen and Heyning (2007) / Štěrba et al. (2000); age reference = Štěrba et al. (2000)
IW1284-14	*Stenella longirostris*	89	78-100	as for *Stenella attenuata*	Thewissen and Heyning (2007) / Štěrba et al. (2000); age reference = Štěrba et al. (2000)
IW1284-15	*Stenella longirostris*	100	95-105	as for *Stenella attenuata*	Thewissen and Heyning (2007) / Štěrba et al. (2000); age reference = Štěrba et al. (2000)
IW1284-16	*Stenella longirostris*	137.5	100-175	as for *Stenella attenuata*	Thewissen and Heyning (2007) / Štěrba et al. (2000); age reference = Štěrba et al. (2000)
IW1284-17	*Stenella longirostris*	240	190-290	as for *Stenella attenuata*	Thewissen and Heyning (2007) / Štěrba et al. (2000); age reference = Štěrba et al. (2000)
IW1284-18	*Stenella longirostris*	280	270-290, birth	as for *Stenella attenuata*	Thewissen and Heyning (2007) / Štěrba et al. (2000); age reference = Štěrba et al. (2000)
IW1284-19	*Stenella longirostris*	492.9	7 months after birth	weaning	http://animaldiversity.ummz.umich.edu/accounts/Stenella_longirostris/ [2014-05-02]
IW1280	*Suncus murinus*				
IW1280-01	*Suncus murinus*	8.75	8.5-9	9	Yasui (1992)
IW1280-02	*Suncus murinus*	8.625	8.5-9.75	10A	Yasui (1992)
IW1280-03	*Suncus murinus*	9.375	9-9.75	10B	Yasui (1992)
IW1280-04	*Suncus murinus*	9.75	9.5-10	10C	Yasui (1992)
IW1280-05	*Suncus murinus*	10	10	11A	Yasui (1993)
IW1280-06	*Suncus murinus*	10.5	10-11	11B	Yasui (1993)
IW1280-07	*Suncus murinus*	11.25	11-11.5	11C	Yasui (1993)
IW1280-08	*Suncus murinus*	11.5	11-12	12A	Yasui (1993)
IW1280-09	*Suncus murinus*	11.75	11.5-12	12B	Yasui (1993)

IW1280-10	*Suncus murinus*	11.75	11.5-12	12C	Yasui (1993)
IW1280-11	*Suncus murinus*	30	30	birth	http://www.issg.org/database/species/ecology.asp?si=162 (2014-02-11)
IW1280-12	*Suncus murinus*	38.5	7-10 after birth	Eye opening	https://taxo4254.wikispaces.com/Suncus+murinus+-+Asian+House+Shrew [2014-04-24]
IW1280-13	*Suncus murinus*	49	19 after birth	weaning	http://genomics.senescence.info/species/entry.php?species=Suncus_murinus [2014-05-02]
IW1288	*Sus scrofa forma domestica*	mainly calculated after Butler and Juurlink (1987)			Butler and Juurlink (1987) only for time reference
IW1288-01	*Sus scrofa forma domestica*	8	8	blastula	Butler and Juurlink (1987): Table 12
IW1288-02	*Sus scrofa forma domestica*	9	x / 9	1	Keibel (1897) / Butler and Juurlink (1987)
IW1288-03	*Sus scrofa forma domestica*	11	x/ 10-12d	2	Keibel (1897) / Butler and Juurlink (1987)
IW1288-04	*Sus scrofa forma domestica*	12.5	x	3	Keibel (1897)
IW1288-05	*Sus scrofa forma domestica*	14	14d	4	Keibel (1897)
IW1288-06	*Sus scrofa forma domestica*	16.5	16d20h	5	Keibel (1897)
IW1288-07	*Sus scrofa forma domestica*	16.625	16d20h	6	Keibel (1897)
IW1288-08	*Sus scrofa forma domestica*	16.75	17d	7	Keibel (1897)
IW1288-09	*Sus scrofa forma domestica*	16.875	17d	8	Keibel (1897)
IW1288-10	*Sus scrofa forma domestica*	17	16d20h	9	Keibel (1897)
IW1288-11	*Sus scrofa forma domestica*	17.125	16d20h	10	Keibel (1897)
IW1288-12	*Sus scrofa forma domestica*	17.25	17d12h	11	Keibel (1897)
IW1288-13	*Sus scrofa forma domestica*	17.5	17d12h	12	Keibel (1897)
IW1288-14	*Sus scrofa forma domestica*	17.75	17d12h	13	Keibel (1897)
IW1288-15	*Sus scrofa forma domestica*	19.5	20d	14	Keibel (1897)
IW1288-16	*Sus scrofa forma domestica*	20	20d	15	Keibel (1897)
IW1288-17	*Sus scrofa forma domestica*	20.5	20d	16	Keibel (1897)
IW1288-18	*Sus scrofa forma domestica*	21	21d	17	Keibel (1897)
IW1288-19	*Sus scrofa forma domestica*	21.5	x / 21-22d	18 / Figure 116	Keibel (1897) / Butler and Juurlink (1987)
IW1288-20	*Sus scrofa forma domestica*	22	22d	19	Keibel (1897)
IW1288-21	*Sus scrofa forma domestica*	22.5	22d	20	Keibel (1897)
IW1288-22	*Sus scrofa forma domestica*	23	x / 23d	21 / Figure 117	Keibel (1897) / Butler and Juurlink (1987)
IW1288-23	*Sus scrofa forma domestica*	23.33	x	22	Keibel (1897)
IW1288-24	*Sus scrofa forma domestica*	23.66	x	23	Keibel (1897)
IW1288-25	*Sus scrofa forma domestica*	24	x / 24d	24 / Figure 118	Keibel (1897) / Butler and Juurlink (1987)
IW1288-26	*Sus scrofa forma domestica*	24.75	x	25 / Figure 119	Keibel (1897)
IW1288-27	*Sus scrofa forma domestica*	25.5	25-26	26	Keibel (1897)
IW1288-28	*Sus scrofa forma domestica*	27.25	x	27	Keibel (1897)
IW1288-29	*Sus scrofa forma domestica*	29	x / 29d	28 / Figure 120	Keibel (1897) / Butler and Juurlink (1987)
IW1288-30	*Sus scrofa forma domestica*	31.5	x	29	Keibel (1897)

IW1288-31	*Sus scrofa forma domestica*	32.5	32.5	Figure 121	Butler and Juurlink (1987)
IW1288-32	*Sus scrofa forma domestica*	36	36		Evans and Sack (1973)
IW1288-33	*Sus scrofa forma domestica*	40	40	Figure 22.23	Schnorr and Kressin (2006)
IW1288-34	*Sus scrofa forma domestica*	49	7 weeks	Figure 8.16A, B	Rüsse and Sinowatz (1991)
IW1288-35	*Sus scrofa forma domestica*	60	60	Figure 15.8	Schnorr and Kressin (2006)
IW1288-36	*Sus scrofa forma domestica*	90	90	Figure 15.12	Schnorr and Kressin (2006)
IW1288-37	*Sus scrofa forma domestica*	114	112-116d	Birth (estimated eye opening)	Evans and Sack (1973)
IW1288-37	*Sus scrofa forma domestica*	179	65 after birth	Weaning [wild boar]	http://genomics.senescence.info/species/entry.php?species=Sus_scrofa [
IW1354	*Talpa europaea*				
IW1354-01	*Talpa europaea*	1.86	x	stage D, primitive streak	Heape (1883a)
IW1354-02	*Talpa europaea*	3.71	x	stage E, Fig. 1-2	Heape (1883b)
IW1354-03	*Talpa europaea*	5.57	x	stage F, Fig. 3-5	Heape (1883b)
IW1354-04	*Talpa europaea*	7.43	x	stage G, Fig. 6	Heape (1883b)
IW1354-05	*Talpa europaea*	9.29	x	stage H, Fig. 7-8	Heape (1883b)
IW1354-06	*Talpa europaea*	11.14	x	stage J, Fig. 9-10	Heape (1883b)
IW1354-07	*Talpa europaea*	13	13	third level	Štěrba (1977)
IW1354-08	*Talpa europaea*	15	15	forth level	Štěrba (1977)
IW1354-09	*Talpa europaea*	18	18	fifth level / Fig. 1A-B; 2A-B	Štěrba (1977) / Štěrba (1980)
IW1354-10	*Talpa europaea*	20	20	sixth level / Fig. 1C, D-F; 2C, D-F	Štěrba (1977) / Štěrba (1980)
IW1354-11	*Talpa europaea*	22	22	seventh level / Fig. 1D-F, 2D-F	Štěrba (1977) / Štěrba (1980)
IW1354-12	*Talpa europaea*	25	25	eigth level	Štěrba (1977)
IW1354-13	*Talpa europaea*	26	26	ninth level	Štěrba (1977)
IW1354-14	*Talpa europaea*	29	29	birth	Štěrba (1977)
IW1354-15	*Talpa europaea*	51	51	Eye opening	Müller (1972)
IW1354-16	*Talpa europaea*	60	31d after birth	weaning	http://genomics.senescence.info/species/entry.php?species=Talpa_europaea [2014-05-02]
IW1278	*Talpa occidentalis*		d		
IW1278-01	*Talpa occidentalis*	16	14-16	s4	Barrionuevo et al. (2004)
IW1278-02	*Talpa occidentalis*	18	17-19	s5	Barrionuevo et al. (2004)
IW1278-03	*Talpa occidentalis*	20	19-21	s6	Barrionuevo et al. (2004)
IW1278-04	*Talpa occidentalis*	22	21-23	s7	Barrionuevo et al. (2004)
IW1278-05	*Talpa occidentalis*	25.5	24-27	s8	Barrionuevo et al. (2004)
IW1278-06	*Talpa occidentalis*	30	28-32 (0-3 postnatal)	s9, birth	Barrionuevo et al. (2004)
IW1278-07	*Talpa occidentalis*	34	32-36 (,3-7 postnatal)	s10	Barrionuevo et al. (2004)
IW1278-08	*Talpa occidentalis*	38.5	36-41 (,7-12 postnatal)	s11	Barrionuevo et al. (2004)
IW1278-09	*Talpa occidentalis*	43.5	41-46 (,12-17 postnatal)	s12	Barrionuevo et al. (2004)
IW1278-10	*Talpa occidentalis*	48.5	46-51(,17-22 postnatal)	s13	Barrionuevo et al. (2004)
IW1278-11	*Talpa occidentalis*	53.5	51-56 (,22-27 postnatal)	s14	Barrionuevo et al. (2004)
IW1278-12	*Talpa occidentalis*	59	56-62 (,27-33 postnatal)	s15a	Barrionuevo et al. (2004)
-	-	-	Permanently closed eyes	Eye opening	Carmona et al. (2010)
IW1278-13	*Talpa occidentalis*	60	1 month of lactation	weaning	Barrionuevo et al. (2004)
IW1355	*Tarsius tarsier ("spectrum")*				
IW1355-01	*Tarsius tarsier*	x	x	Figure 1	Hubrecht and Keibel (1907)
IW1355-02	*Tarsius tarsier*	x	x	Figure 2	Hubrecht and Keibel (1907)
IW1355-03	*Tarsius tarsier*	x	x	Figure 3	Hubrecht and Keibel (1907)
IW1355-04	*Tarsius tarsier*	x	x	Figure 4	Hubrecht and Keibel (1907)
IW1355-05	*Tarsius tarsier*	x	x	Figure 5	Hubrecht and Keibel (1907)
IW1355-06	*Tarsius tarsier*	x	x	Figure 6	Hubrecht and Keibel (1907)
IW1355-07	*Tarsius tarsier*	x	x	Figure 7	Hubrecht and Keibel (1907)
IW1355-08	*Tarsius tarsier*	x	x	Figure 8	Hubrecht and Keibel (1907)
IW1355-09	*Tarsius tarsier*	x	x	Figure 9	Hubrecht and Keibel (1907)
IW1355-10	*Tarsius tarsier*	x	x	Figure 10	Hubrecht and Keibel (1907)
IW1355-11	*Tarsius tarsier*	x	x	Figure 11	Hubrecht and Keibel (1907)
IW1355-12	*Tarsius tarsier*	x	x	Figure 12	Hubrecht and Keibel (1907)
IW1355-13	*Tarsius tarsier*	x	x	Figure 13	Hubrecht and Keibel (1907)
IW1355-14	*Tarsius tarsier*	x	x	Figure 14	Hubrecht and Keibel (1907)
IW1355-15	*Tarsius tarsier*	x	x	Figure 15	Hubrecht and Keibel (1907)
IW1355-16	*Tarsius tarsier*	x	x	Figure 16	Hubrecht and Keibel (1907)
IW1355-17	*Tarsius tarsier*	x	x	Figure 17	Hubrecht and Keibel (1907)
IW1355-18	*Tarsius tarsier*	x	x	Figure 18	Hubrecht and Keibel (1907)
IW1355-19	*Tarsius tarsier*	x	x	Figure 19	Hubrecht and Keibel (1907)
IW1355-20	*Tarsius tarsier*	x	x	Figure 20	Hubrecht and Keibel (1907)

IW1355-21	*Tarsius tarsier*	x	x	Notebook 33, image 2069	Embryological Collection Berlin: Hill collection
IW1355-22	*Tarsius tarsier*	180	6 months	Birth (estimated eye opening)	Hayssen et al. (1993)
IW1355-23	*Tarsius tarsier*	260	80 after birth	weaning	http://pin.primate.wisc.edu/factsheets/entry/tarsier/behav [2014-05-02]
IW1417	*Tupaia belangeri*				
IW1417-01	*Tupaia belangeri*	9	9	stage IV	Kuhn and Schwaier (1973); summarized by Butler and Juurlink (1987)
IW1417-02	*Tupaia belangeri*	12	12	stage V	Kuhn and Schwaier (1973); summarized by Butler and Juurlink (1987)
IW1417-03	*Tupaia belangeri*	14	14	stage VII	Kuhn and Schwaier (1973); summarized by Butler and Juurlink (1987)
IW1417-04	*Tupaia belangeri*	18	18	stage VIII	Kuhn and Schwaier (1973); summarized by Butler and Juurlink (1987)
IW1417-05	*Tupaia belangeri*	24	24	stage IX	Kuhn and Schwaier (1973); summarized by Butler and Juurlink (1987)
IW1417-06	*Tupaia belangeri*	29	29	stage X	Kuhn and Schwaier (1973); summarized by Butler and Juurlink (1987)
IW1417-07	*Tupaia belangeri*	44	42-46	birth	Kuhn and Starck (1966)
IW1417-08	*Tupaia belangeri*	56	12d after birth	young	Kuhn and Starck (1966)
IW1417-09	*Tupaia belangeri*	61.5	16-19d after birth	figure 6, open eye	Kuhn and Starck (1966)
IW1417-10	*Tupaia belangeri*	80	36d after birth	weaning	http://genomics.senescence.info/species/entry.php?species=Tupaia_belangeri [2014-05-02]
IW1281	*Tupaia javanica*				
IW1281-01	*Tupaia javanica*	x	x	1	de Lange and Nierstrasz (1932)
IW1281-02	*Tupaia javanica*	x	x	2	de Lange and Nierstrasz (1932)
IW1281-03	*Tupaia javanica*	x	x	3	de Lange and Nierstrasz (1932)
IW1281-04	*Tupaia javanica*	x	x	4	de Lange and Nierstrasz (1932)
IW1281-05	*Tupaia javanica*	x	x	5	de Lange and Nierstrasz (1932)
IW1281-06	*Tupaia javanica*	x	x	6	de Lange and Nierstrasz (1932)
IW1281-07	*Tupaia javanica*	x	x	7	de Lange and Nierstrasz (1932)
IW1281-08	*Tupaia javanica*	x	x	8	de Lange and Nierstrasz (1932)
IW1281-09	*Tupaia javanica*	x	x	9	de Lange and Nierstrasz (1932)
IW1281-10	*Tupaia javanica*	x	x	10	de Lange and Nierstrasz (1932)
IW1281-11	*Tupaia javanica*	x	x	11	de Lange and Nierstrasz (1932)
IW1281-12	*Tupaia javanica*	x	x	12	de Lange and Nierstrasz (1932)
IW1281-13	*Tupaia javanica*	x	x	13	de Lange and Nierstrasz (1932)
IW1281-14	*Tupaia javanica*	x	x	14	de Lange and Nierstrasz (1932)
IW1281-15	*Tupaia javanica*	x	x	15	de Lange and Nierstrasz (1932)
IW1281-16	*Tupaia javanica*	x	x	16	de Lange and Nierstrasz (1932)
IW1281-17	*Tupaia javanica*	x	x	17	de Lange and Nierstrasz (1932)
IW1281-18	*Tupaia javanica*	x	x	18	de Lange and Nierstrasz (1932)
IW1281-19	*Tupaia javanica*	x	x	19	de Lange and Nierstrasz (1932)
IW1281-20	*Tupaia javanica*	x	x	20	de Lange and Nierstrasz (1932)
IW1281-21	*Tupaia javanica*	x	x	21	de Lange and Nierstrasz (1932)

IW1281-22	*Tupaia javanica*	x	x	22	de Lange and Nierstrasz (1932)
IW1281-23	*Tupaia javanica*	x	x	23	de Lange and Nierstrasz (1932)
IW1281-24	*Tupaia javanica*	x	x	24	de Lange and Nierstrasz (1932)
IW1281-25	*Tupaia javanica*	50	50	birth	http://www.theanimalfiles.com/mammals/tree_shrews/horsfields_tree_shrew.html (2014-02-11)
IW1281-26	*Tupaia javanica*	70	About 20d after birth	Eye opening (general for tree shrews)	Hayssen et al. (1993)
IW1281-27	*Tupaia javanica*	x	x	weaning	x

References

Abbas, Y., 2006. Animals in the Womb, Pioneer Productions: United Kingdom.

Acker, D.A., Curran, S., Bersu, E.T., Ginther, O.J., 2001. Morphologic stages of the equine embryo proper on days 17 to 40 after ovulation. American Journal of Veterinary Research 62, 1358-1364.

Adams, R.A., 1992. Stages of development and sequence of bone formation in the little brown bat, *Myotis lucifugus*. Journal of Mammalogy 73, 160-167.

Allen, W.R., 2006. Ovulation, pregnancy, placentation and husbandry in the African elephant (*Loxodonta africana*). Phil. Trans. R. Soc. Lond. B 361, 821-834.

Bagwell, J.N., Leavitt, W.W., 1974. Prenatal size-age relationships and external morphology in the Mongolian gerbil (*Merionex unguiculatus*). American Journal of Anatomy 140, 117-128.

Barrionuevo, F.J., Zurita, F., Burgos, M., Jiménez, R., 2004. Developmental stages and growth rate of the mole *Talpa occidentalis* (Insectivora, Mammalia). Journal of Mammalogy 85, 120-125.

Beaudoin, S., Nbarbet, P., Bargy, F., 2003. Developmental stages in the rabbit embryo: guidelines to choose an appropriate experimental model. Fetal Diagnosis and Therapy 18, 422-427.

Betteridge, K.J., Eaglesome, M.D., Mitchell, D., Flood, P.F., Beriault, R., 1982. Development of horse embryos up to 22 days after ovulation - observations on fresh specimens. Journal of Anatomy 135, 191-209.

Binkerd, P.E., Hendrickx, A.G., Rice, J.M., Palmer, A.E., 1984. Embryonic development in *Erythrocebus patas*. American Journal of Primatology 6, 15-29.

Bischoff, T.L.W., 1845. Entwicklungsgeschichte des Hunde-Eies. Druck und Verlag von Friedrich Vieweg und Sohn, Braunschweig.

Bischoff, T.L.W., 1852. Entwicklungsgeschichte des Meerschweinchens. J. Ricker'sche Buchhandlung, Gießen.

Bonnet, R., 1897. Beiträge zur Embryologie des Hundes. Anatomische Hefte 9, 419-512.

Bonnet, R., 1901. Beiträge zur Embryologie des Hundes. Erste Fortsetzung. Anatomische Hefte 16, 231-332.

Bonnet, R., 1902. Beiträge zur Embryologie des Hundes. Zweite Fortsetzung. Anatomische Hefte, 323-499.

Boyer, C.C., 1953. Chronology of development for the golden hamster. Journal of Morphology 92.

Bryden, M.M., Binns, W., Evans, H.E., 1972. Embryology of sheep.1. extraembryonic membranes and development of body form. Journal of Morphology 138, 169-&.

Butler, H., 1972. The chronology of embryogenesis in the lesser galago: a preliminary account. Folia primatologica 18, 368-378.

Butler, H., Juurlink, B.H.J., 1987. An atlas for staging mammalian and chick embryos. CRC Press, Inc., Boca Raton.

Cloutier, D., Thomas, D.W., 1992. *Carollia perspicillata*. Mammalian Species 417, 1-9.

Concil, N.R., 1995. Nutrient Requirements of Laboratory Animals. National Academic Press, Washington D.C.

Cretekos, C.J., Weatherbee, S.D., Chen, C.H., Badwaik, N.K., Niswander, L., Behringer, R.R., Rasweiler, J.J.I.V., 2005. Embryonic staging system for the short-tailed fruit bat, *Carollia perspicillata*, a model organism for the mammalian order Chiroptera, based upon timed pregnancies in captive-bred animals. Developmental Dynamics 233, 721-738.

de Lange, D., Nierstrasz, H.F., 1932. Tabellarische Uebersicht der Entwicklung von *Tupaia javanica* HORSF. A. Oosthoek Verlag, Utrecht.

Dieterlen, F., 1963. Vergleichende Untersuchungen zur Ontogenese von Stachelmaus (*Acomys*) und Wanderratte (*Rattus norvegicus*). Beiträge zum Nesthocker-Nestflüchter-Problem bei Nagetieren. Zeitschrift für Säugetierkunde 28, 193-227.

Dryden, G.L., Gebczynski, M., Douglas, E., 1974. Oxygen consumption by nursling and adult musk shrews. Acta Theriol 19, 453-461.
Evans, H.E., Sack, W.O., 1973. Prenatal development of domestic and laboratory mammals: growth curves, external features and selected references. Anatomia Histologia Embryologia 2, 11-45.
Ewart, J.C., 1917. Studies on the development of the horse. I. The development during the third week. Transactions of the Royal Society of Edinburgh 51, 287-329, 210 plates.
Fernandez, M., 1915. Die Entwicklung der Mulita - La embriología de la Mulita (*Tatusia hybrida* Desm.). Revista del Museo de la Plata 21, 519.
Ferner, K., Zeller, U., Schmelting, B., Fuchs, E., 2010. Ontogenetic and lung development in *Tupaia belangeri* during the early postnatal period. Mamm. Biol. 75, 95-105.
Gaeth, A.P., Short, R.V., Renfree, M.B., 1999. The developing renal, reproductive, and respiratory systems of the African elephant suggest an aquatic ancestry. Proceedings of the National Academy of Sciences of the United States of America 96, 5555-5558.
Gemell, R.T., Nelson, J., 1992. Development of the vestibular and auditory system of the northern native cat, *Dasyurus hallucatus*. Anatomical Record 234, 136-143.
Gill, E.L., 1926. An early embryo of the blue whale. Transactions of the Royal Society of South Africa 14, 295-300.
Göbbel, L., 2002. Morphology of the external nose in *Hipposideros diadema* and *Lavia frons* with comments on its diversity and evolution among leaf-nosed Microchiroptera. Cells, tissues, organs 170, 39-60.
Good, J.P., 1912. Spina bifida in the neck region of a ferret embryo 8 mm long. Journal of Anatomy and Physiology 46, 391-399.
Gulamhusein, A.P., Beck, F., 1981. External features of the developing ferret embryo. Bibliotheca anatomica 19, 236-246.
Gulamhusein, A.P., Beck, F., 1983. Normal and abnormal appearances of the 35-day ferret fetus studied by freehand (Razor Blade) sections and gross examination. Teratology 27, 371-384.
Gursky, S., 2000. Allocare in a nocturnal primate: Data on the spectral tarsier, *Tarsius spectrum*. Folia Primatol. 71, 39-54.
Hall, M.H., 1990. Definitions used in relation to gestational age. Paediatr. Perinat. Epidemiol. 4, 123-128.
Hamilton, W.J., 1939. The early stages of the development of the ferret: the formation of the mesoblast and notochord. Transactions of the Royal Society of Edinburgh 59, 165-193, 167 plates.
Harman, M.T., Prickett Dobrovolny, M., 1933. The development of the external form of the guinea-pig (*Cavia cobaya*) between the ages of 21 days and 35 days of gestation. Journal of Morphology 54, 493-519.
Harrison, B.M., Mohn, L.A., 1932. Some stages in the development of the pharynx of the embryo horse. American Journal of Anatomy 50, 233-250.
Hayssen, V., 2009. *Bradypus tridactylus* (Pilosa: Bradypodidae). Mammalian Species 839.
Hayssen, V., van Tienhoven, A., van Tienhoven, A., 1993. Asdell's Patterns of Mammalian Reproduction: A Compendium of Species-Specific Data. Cornell University Press, Ithaca and London.
Heape, W., 1883b. The development of the mole (*Talpa europaea*). Stages E to J. Quarterly Journal of Microscopical Science 27, 123-162.
Heape, W., 1886a. The development of the mole (*Talpa europaea*), the ovarian ovum, and segmentation of the ovum. Quarterly Journal of Microscopical Science 26, 157-174.
Hendrickx, A.G., 1971. Embryology of the Baboon. University of Chicago Press, Chicago, London.
Henneberg, B., 1937. Normentafel zur Entwicklungsgeschichte der Wanderratte (*Rattus norvegicus* Erxleben). Verlag von Gustav Fischer, Jena.

Heuser, C.H., Wislocki, G.B., 1935. Early development of the sloth (*Bradypus griseus*) and its similarity to that of man. Contributions to Embryology.

Hildebrandt, T., Drews, B., Gaeth, A.P., Goeritz, F., Hermes, R., Schmitt, D., Gray, C., Rich, P., Streich, W.J., Short, R.V., Renfree, M.B., 2007. Foetal age determination and development in elephants. Proceedings of the Royal Society B-Biological Sciences 274, 323-331.

Hill, J.P., 1932. The developmental history of primates. Philosophical Transactions of the Royal Society of London. Series B 221, 45-178, 121 plates.

Hockman, D., Mason, M.K., Jacobs, D.S., Illing, N., 2009. The role of early development in mammalian limb diversification: a descriptive comparison of early limb development between the natal long-fingered bat (*Miniopterus natalensis*) and the mouse (*Mus musculus*). Developmental Dynamics 238, 965-979.

Hubrecht, A.A.W., Keibel, F., 1907. Normentafel zur Entwicklungsgeschichte des Koboldmakis (*Tarsius spectrum*) und des Plumlori (*Nycricebus tardigradus*). Verlag von Gustav Fischer, Jena.

Huisman, F.J., 1933. Tabellarische Übersicht der Entwicklung von *Manis javanica* Desm. N.V. A. Oosthoeck's Uitgevers-Maatschappij, Utrecht.

Izard, M.K., Weisenseel, K., Ange, R., 2005. Reproduction in the slow loris (*Nycticebus coucang*). Am. J. Primatol. 16, 331-339.

Jacobfeuerborn, H., 1908. Die intrauterine Ausbildung der äußeren Körperform des Igels (*Erinaceus europaeus* L.) mit Berücksichtigung der Entwicklung der wichtigeren inneren Organe. Zeitschrift für wissenschaftliche Zoologie 91, 382-420.

Keibel, F., 1897. Normentafel zur Entwicklungsgeschichte des Schweines (*Sus scrofa domesticus*). Verlag von Gustav Fischer, Jena.

Keibel, F., 1906. Die Entwickelung der äußeren Körperform der Wirbeltierembryonen, insbesondere der menschlichen Embryonen aus den ersten 2 Monaten, in: Hertwig, O. (Ed.), Handbuch der vergleichenden und experimentellen Entwicklungslehre der Wirbeltiere. Verlag von Gustav Fischer, Jena, pp. 1-174.

Keibel, F., Elze, C., 1908. Normentafel zur Entwicklungsgeschichte des Menschen. Verlag von Gustav Fischer, Jena.

Koike, K., 1924. Die Herausbildung der äußeren Körperform und der Entwicklungsgrad der Organe bei einer javanischen Kleinfledermaus (*Scotophilus temmincki*, Hoesfield). Zeitschrift für Anatomie und Entwicklungsgeschichte 72.

Kuhn, H.J., Schwaiger, A., 1973. Implanation, early placentation, and the chronology of embryogenesis in *Tupaia belangeri*. Zeitschrift für Anatomie und Entwicklungsgeschichte 142, 315-340.

Kuhn, H.J., Starck, D., 1966. Die *Tupaia*-Zucht des Dr. Senckenbergischen Anatomischen Institutes. Natur und Museum 96, 263-274.

Kükenthal, W., 1893. Vergleichend-anatomische und entwicklungsgeschichtliche Untersuchungen an Walthieren. Zweiter Theil. Verlag von Gustav Fischer, Jena.

Laska, M., 1990. Gestation period and between-birth intervals in *Carollia perspicillata* (Phyllostomatidae, Chiroptera). Journal of Zoology 222, 697-702.

Laurie, E., 1946. The reproduction of the house-mouse (*Mus musculus*) living in different environments. Proc. R. Soc. Lond., B, Biol. Sci. 133, 248-281.

Laurien-Kehnen, C., Trillmich, F., 2004. Maternal food restriction delays weaning in the guinea pig, *Cavia porcellus*. Anim. Behav. 68, 303-312.

Lim, N.T.L., Ng, P.K.L., 2007. Home range, activity cycle and natal den usage of a female Sunda pangolin *Manis javanica* (Mammalia: Pholidota) in Singapore. Endangered Species Research 3, 1-8.

Mate, K.E., Robinson, E.S., Vandeberg, J.L., Pedersen, R.A., 1994. Timetable of in vivo embryonic development in the grey short-tailed opossum (*Monodelphis domestics*). Development 365474.

McManus, J.J., 1971. Early postnatal growth and the development of temperature regulation in the Mongolian gerbil, *Meriones unguiculatus*. J. Mammal. 52, 782-792.
Meikle, D., Westberg, M., 2001. Maternal nutrition and reproduction of daughters in wild house mice (*Mus musculus*). Reproduction 122, 437-442.
Michl, E., 1920. Beitrag zur Entwicklungsgeschichte von *Bos taurus* L. Anatomischer Anzeiger 53, 193-215.
Miglino, M.A., Ambrósio, C.E., dos Santos Martins, D., Wenceslau, C.V., Pfarrer, C., Leiser, R., 2006. The carnivore pregnancy: the development of the embryo and fetal membranes. Theriogenology 66, 1699-1702.
Minot, C.S., Taylor, E., 1905. Normal plates of the development of the rabbit (*Lepus cuniculus* L.), in: Keibel, F. (Ed.), Normentafeln zur Entwicklungsgeschichte der Wirbeltiere. Gustav Fischer Verlag, Jena.
Morii, R., 1980. Postnatal development of external characters and behavior in young *Pipistrellus abramus*. Journal of the Mammalogical Society of Japan 8, 117-121.
Müller, F., 1972-1973. Zur stammesgeschichtlichen Veränderung der Eutheria-Ontogenesen. Versuch einer Übersicht aufgrund vergleichend morphologischer Studien an Marsupialia und Eutheria. 1. Teil. Zur Evolution der Geburtsgestalt: Gestaltstadien der Eutheria. / 2. Teil. Ontogenesetypus und Cerebralisation. / 3. Teil. Zeitliche Aspekte in der Evolution der Ontogenesetypen. / 4. Spezieller Teil. . Revue suisse de zoologie 79, 1-97, 501-566, 567-612, 1599-1685.
Müller, H.C., 1920. Zur Entwicklungsgeschichte von *Phocaena communis* Less. Archiv für Naturgeschichte. Abteilung A 86, 1-112.
Nolte, M.J., Hockman, D., Cretekos, C.J., Behringer, R.R., Rasweiler, J.J.I., 2009. Embryonic staging system for the black mastiff bat, *Molossus rufus* (Molossidae), correlated with structure-function relationships in the adult. The Anatomical Record 292, 155-168.
O'Rahilly, R., Müller, F., 1987. Development Stages in Human Embryos - Including a Revision of Streeter's "Horizons" and a Survey of the Carnegie Collection. Carnegie Institute of Washington, Washington DC.
Parker, S., 1990. Grzimek's Encyclopedia of Mammals. McGraw-Hill Publishing Company, New York.
Pelz, H.-J., Gemmeke, H., Hutterer, R., Jüdes, U., 1996. Jugendentwicklung der Brandmaus *Apodemus agrarius* (Mammalia: Muridae) im Vergleich zu anderen Arten der Gattung. Bonner Zoologische Beiträge 46, 233-247.
Petermann, 1907. Zur Kenntnis der frühen Entwicklungsvorgänge am Ei des Igels (*Erinaceus europaeus* L.) vor Ausbildung der Medullarrinne. Zeitschrift für wissenschaftliche Zoologie 85, 305-361.
Phillips, I.R., 1976. The Embryology of the Common Marmoset (*Callithrix jacchus*). Springer-Verlag, Berlin.
Poux, C., Madsen, O., Glos, J., de Jong, W.W., Vences, M., 2008. Molecular phylogeny and divergence times of Malagasy tenrecs: influence of data partitioning and taxon sampling on dating analyses. BMC Evolutionary Biology 8.
Radford, M., 1911. Reconstruction of the head end of an early feret embryo. Journal of Anatomy and Physiology 45, 336-346.
Raubenheimer, E.J., 2000. Early development of the tush and the tusk of the African elephant (*Loxodonta africana*). Archives of Oral Biology 45, 983-986.
Retzius, G., 1900. Zur Kentniss der Entwicklungsgeschichte des Rennthieres und des Rehes. Biologische Untersuchungen 9, 109-117.
Rhine, R.J., Norton, G.W., Wynn, G.M., Wynn, R.D., 1985. Weaning of free-ranging infant baboons (*Papio cynocephalus*) as indicated by one-zero and instantaneous sampling of feeding. International Journal of Primatology 6, 491-499.
Robinson, A., Gibson, A., 1917. Description of a reconstruction of a horse embryo twenty-one days old. Transactions of the Royal Society of Edinburgh 51, 331-346.

Ross, C., 1991. Life history patterns of New World monkeys. Int. J. Primatol. 12, 481-502.
Rüsse, I., 1991. Frühgravidität, Implanation und Plazentation, in: Rüsse, I., Sinowatz, F. (Eds.), Lehrbuch der Embryologie der Haustiere. Verlag Paul Parey, Berlin, Hamburg.
Rüsse, I., 1991. Gesicht und Körperform, in: Rüsse, I., Sinowatz, F. (Eds.), Lehrbuch der Embryologie der Haustiere. Parey, Berlin, Hamburg.
Rüsse, I., Sinowatz, F., 1991. Lehrbuch der Embryologie der Haustiere. Parey, Berlin, Hamburg.
Sakurai, T., 1906. Normentafel zur Entwicklungsgeschichte der des Rehes (*Cervus capreolus*). Verlag von Gustav Fischer, Jena.
Schnorr, B., Kressin, M., 2006. Embryologie der Haustiere. Enke Verlag, Stuttgart.
Scott, J.P., 1937. The embryology of the guinea pig I. A table of normal development. American Journal of Anatomy 60, 397-432.
Smith, K.K., 1997. Comparative patterns of craniofacial development in eutherian and metatherian mammals. Evolution 51, 1663-1678.
Smith, N.S., Sowls, K.L., 1975. Fetal development of the collared peccary. Journal of Mammalogy 56, 6199-6625.
Štěrba, O., 1976. Prenatal development of microtine rodents. Acta Sc. Nat. Brno 10, 1-41.
Štěrba, O., 1977a. Prenatal development of central europaean insectivores. Folia Zoologica 26, 27-44.
Štěrba, O., 1977b. Prenatal development of selected altricial and precocial rodents. Acta Sc. Nat. Brno 11, 1-36.
Štěrba, O., 1980. The timing of the prenatal development of the position of the limbs in the common mole, *Talpa europaea*. Folia Zoologica 29, 225-233.
Štěrba, O., 1990. Prenatal development of *Myotis myotis* and *Miniopterus schreibersi*. Folia Zoologica 39, 73-83.
Štěrba, O., Klima, M., Schildger, B., 2000. Embryology of Dolphins. Staging and Aging of Embryos and Fetuses of Some Cetaceans. Springer, Berlin.
Symonds, M.R.E., 2006. Life histories of the Insectivora: the role of phylogeny, metabolism and sex differences. J. Zool. 249, 315-337.
ten Donkelaar, H.J., Geysberts, L.G.M., Dederen, P.J.W., 1979. Stages in the prenatal development of the chinese hamster (*Cricetulus griseus*). Anatomy and Embryology 156, 1-28.
Theiler, K., 1989. The House Mouse: Atlas of Embryonic Development. Spinger Verlag, New York.
Thewissen, J.G.M., Heyning, J., 2007. Embryogenesis and development in *Stenella attenuata* and Other Cetaceans, in: Miller, D.L. (Ed.), Reproductive Biology and Phylogeny of Cetacea. Whales, Dolphins and Porpoises. Science Publisher, Enfield.
Tokita, M., 2006. Normal embryonic development of the Japanese pipistrell, *Pipistrellus abramus*. Zoology 109, 137-147.
Townend, L., 2009. In the womb: dogs, USA.
Tsang, W., Collins, P., 1985. Techniques for hand-rearing tree-shrews (*Tupaia belangeri*) from birth. Zoo Biol. 4, 23-31.
Tsukaguchi, R., 1912. Zur Entwicklungsgeschichte der Ziege (*Capra hircus*). Beiträge zur Entwicklung der Wiederkäuer. Anatomische Hefte 46.
van Oordt, G.J., 1921. Early developmental stages of *Manis javanica* DESM. Verhandelingen Koninklijke Akademie van Wetenschappen te Amsterdam. Tweede Sectie 21, 1-102.
Vaughan, T.A., Ryan, J.M., Czaplewski, N.J., 2011. Mammalogy. Jones & Bartlett Publ., USA.
Vihervaara, H., Sundell, J., Ylönen, H., 2010. Is mating alone enough to inhibit infanticide in male bank voles? Ethology 116, 888-894.
Vitums, A., 1969. Developmental transformations of the aortic arches in the equine embryos with special attention to the formation of the definitive arch of the aorta and the common

branchiocephalic trunk. Zeitschrift für Anatomie und Entwicklungsgeschichte 128, 263-270.
Vogel, P., 1972. Vergleichende Untersuchung zum Ontogenesemodus einheimischer Soriciden (Croidura russula, Sorex araneus und Neomys fodiens). Revue suisse de zoologie 79, 1201-1332.
Völker-Brünn, O., 1922. Normentafel zur Entwicklungsgeschichte des Ziesels (*Spermophilus citillus*). Verlag von Gustav Fischer, Jena.
Wang, Z., Han, N., Racey, P.A., Ru, B., He, G., 2010. A comparative study of prenatal development in *Miniopterus schreibersii fuliginosus*, *Hipposideros armiger* and *H. pratti*. BMC Developmental Biology 10, 1-17.
Watson, C., Provis, J., Herculano-Houzel, S., 2012. What determines motor neuron number? Slow scaling of facial motor neuron numbers with body mass in marsupials and primates. Anat. Rec. 295, 1683-1691.
Weber, M., 1892. Beiträge zur Anatomie und Entwickelung des Genus *Manis*, Leiden.
West, C.D., Kemper, T.L., 1976. The effect of a low protein diet on the anatomical development of the rat brain. Brain Research 107, 221-237.
Williams, K., Parer, I., Coman, B., Burley, J., Braysher, M., 1995. Managing Vertebrate Pests: Rabbits. Bureau of Resource Sciences and CSIRO Division of Wildlife and Ecology. Australian Government Publishing Service, Canberra.
Wilson, D.E., Reeder, D.M., 2005. Mammal Species of the World. Johns Hopkins University Press, Baltimore, Maryland.
Yasui, K., 1992. Embryonic development of the house shrew (*Suncus murinus*). Anatomy and Embryology 186, 49-65.
Yasui, K., 1993. Embryonic development of the house shrew (*Sucus murinus*). II. Embryos at stages 11 and 12 with 13 to 29 pairs of somites, showing limb bud formation and closed cephalic tubes. Anatomy and Embryology 187, 45-65.
Yeates, T., 1911. Studies in the embryology of the ferret. Journal of Anatomy and Physiology 45, 319-335.
